# Lifestyle Recommendations for Patients Before and After Thoracic Aortic Surgery: A Framework Analysis

**DOI:** 10.1055/s-0044-1791668

**Published:** 2024-10-29

**Authors:** Niek Koenders, Henrita van Zetten, Michelle Smulders, Hans Smeenk, Roland van Kimmenade, Tim Smith, Guillaume Geuzebroek, Thomas van Brakel, Michel Verkroost

**Affiliations:** 1 Department of Rehabilitation, Radboud University Medical Center, Nijmegen, the Netherlands; 2 Department of Cardiothoracic Surgery, Radboud University Medical Center, Nijmegen, the Netherlands; 3 Department of Cardiothoracic Surgery, St. Antonius Hospital, Nieuwegein, the Netherlands; 4 Department of Cardiology, Radboud University Medical Center, Nijmegen, the Netherlands; 5 Department of Cardiothoracic Surgery, Catharina Hospital, Eindhoven, the Netherlands

**Keywords:** lifestyle, health behavior, thoracic aorta, thoracic aortic aneurysm, cardiac surgery

## Abstract

**Background** Patients receive many different recommendations after thoracic aortic surgery. Unfortunately, there is much variation in recommendations between different surgical centers. This variation in recommendations creates uncertainty and anxiety in patients. Therefore, we aimed to provide an overview with clear lifestyle recommendations for patients before and after thoracic aortic surgery.

**Methods** Documentary research and a framework analysis were used to analyze brochures, website texts, and health care protocols. These documents consisted of lifestyle recommendations for patients before and after thoracic aortic surgery (direct information) or cardiac surgery (indirect information). An analytical framework was constructed and all lifestyle recommendations for patients before and after thoracic aortic surgery were coded through indexing, charting, and mapping by two researchers (N.K. and H.v.Z.). The first draft with lifestyle recommendations was prepared by two researchers (N.K. and H.v.Z.). Feedback from all authors involved patients and consulted health care professionals was processed in the final draft.

**Results** In total, 170 documents were analyzed. Indexing revealed 414 lifestyle recommendations, which were included in the first draft. Charting, mapping, removal of duplicates, and processing of feedback resulted in a final draft with 52 lifestyle recommendations about behavioral change, body weight, nutrition, cessation of alcohol and drug use, cessation of smoking, wound healing, sedentary behavior and physical activity, mental well-being, and family and close relatives.

**Conclusion** This study provides an overview of clear lifestyle recommendations for patients before and after thoracic aortic surgery. This overview is the first step because follow-up research is needed on which lifestyle recommendations are necessary and evidence-based. The overview of lifestyle recommendations serves as a foundation, after which individual customization can be provided.

## Introduction

Thoracic aortic diseases, such as thoracic aortic aneurysms and dissections, occur in approximately 5 to 8 per 100,000 individuals per year. 1 However, the calculation of the prevalence and incidence of thoracic aortic disease is usually biased because individuals are mostly asymptomatic. 2 Recent improvements in screening procedures increase the prevalence of thoracic aortic disease and improvements in surgical techniques increase survival. 3 4 As a result, more and more attention should be paid to long-term health and secondary prevention.

To improve long-term health, guidelines indicate the importance of lifestyle goals for patients with thoracic aortic disease. 5 6 7 Amongst other factors, regular exercise, a low-fat low-salt diet, and achieving ideal body weight help patients to effectively control their blood pressure and associated aortic wall stress. 5 Current lifestyle recommendations focus mainly on restrictions, such as avoiding heavy lifting or pushing. However, *restrictions* are not very helpful in terms of knowing what would be healthy to do, and they lead to increased distress and anxiety in patients before and after thoracic aortic surgery. 8 Health care professionals point out that lifestyle *recommendations* are important for patients to adequately cope with their illness. 9 10 However, they lack an overview of clear lifestyle recommendations, resulting in a great deal of variation in practice. This variation in practice might further increase patients' distress and anxiety. 11 Therefore, the aim of this study is to provide an overview with clear lifestyle recommendations for patients before and after thoracic aortic surgery.

## Materials and Methods

### Design

This study used documentary research 12 and framework analysis 13 to examine lifestyle recommendations for patients before and after thoracic aortic surgery in brochures, website texts, and health care protocols. Lifestyle recommendations were defined as “instructions for the way of life of people before or after thoracic aortic surgery on topics such as exercise, diet, and smoking,” in accordance with the National Prevention Policy of the Ministry of Health, Welfare and Sport. 14 Instructions for medication use were not considered lifestyle recommendations in this definition and, therefore, not included in this study. Thoracic aortic surgery concerns all cardiothoracic surgery on the aortic root (including Bentall surgery), ascending aorta, aortic arch, and/or descending thoracic aorta using open procedures. It does not involve isolated aortic valve replacement, abdominal aortic surgery, endovascular procedures, or conservative treatment.

### Participating Centers

All cardiac surgery centers in the Netherlands ( *n* = 16) were approached by email for participation in this study by a researcher (N.K.). In addition, regional cardiac rehabilitation centers in the Netherlands ( *n* = 13) were contacted for participation by email. The staff of the potential participating center was emailed with a request to nominate a contact person at the cardiac surgery department. This contact person received an information letter and digital informed consent for study participation. The contact person was then asked to share all documents for patients before and after thoracic aortic surgery until July 2021.

### Data Sources

The documents collected were divided into three major categories. The first category of documents was brochures: small booklets published by a center's communications department, often printed, intended to inform patients about all the aspects of thoracic aortic surgery. The second category was website texts: a collection of data on web pages stored or hosted on the official web page of a center. The third category was health care protocols: documents that aim to support health care professionals in performing care-related actions, indicating how, when, and what lifestyle recommendations can be provided. The documents contained information for patients before and after thoracic aortic surgery such as aortic valve-sparing operations for aortic root aneurysm (direct information) and patients facing cardiac surgery, such as aortic valve replacement or coronary artery bypass graft surgery (indirect information). The indirect information was not removed a priori, however, it was removed during the data analysis and is not the subject of this study. Video data and information on digital health applications were outside the scope of this study.

### Data Analysis

The details of the framework analysis and the thematic framework are presented in Tables 1 and 2 . Framework analysis was used to examine data from included brochures, website texts, and health care protocols. 13 The software package Atlas.ti 8.4.20 was used to support data analysis through several steps. 15

**Table 1:** Details of the framework analysis

Step	Details
1. Familiarization	Two authors (N.K. and H.v.Z.) independently familiarized themselves with relevant guidelines and performed a cursory reading of the dataset.
2. Identifying a thematic framework	A framework was constructed by two authors (N.K. and H.v.Z.) using the lifestyle recommendations modules of the third review Dutch Cardiovascular Risk Management Guideline 9 and the Revision Dutch Guideline Cardiovascular Disease Prevention 2019 19 ( Table 2 ).
3. Indexing	Two authors (N.K. and H.v.Z.) independently used the thematic framework across all brochures, website texts, and health care protocols. Lifestyle recommendations data were labeled according to the themes in the thematic framework. Only information on thoracic aortic surgery (direct information) was labeled. The thematic framework was extended with the themes “alcohol and drug use,” “wound healing,” “mental well-being,” and “family and close relatives” to include all relevant research data ( Table 2 ). The data were indexed by comparing all textual data to the themes of the framework. This step consisted of evaluating the definitions of each theme and assessing if the document had elements that would warrant it to be classified as belonging to and/or answering the characteristics of that theme. Classification of the data for each theme relied on the content, not on the intended audience or aim of a data source.
4. Charting	This step focused on data extraction. Atlas.ti provided a spreadsheet that included the name of each document in the document manager and codes with all labels in the code manager. Document notes were used to provide reasons for data exclusion, for example, when a document was not about cardiac surgery (i.e., aortic valve replacement) instead of thoracic aortic surgery.
5. Mapping	Ultimately, two authors (N.K. and H.v.Z.) discussed all labels together and reached an agreement. One memo was created for each theme in the memo manager: behavioral change, body weight, nutrition, cessation of alcohol and drug use, cessation of smoking, wound healing, sedentary behavior and physical activity, mental well-being, and family and close relatives. These memos were examined for patterns and discrepancies in the dataset, with an emphasis on creating clear lifestyle recommendations for patients with thoracic aortic surgery.
6. Checking duplicates	All memos were checked by two authors (N.K. and H.v.Z.) and duplicate lifestyle advice were removed.
7. First draft	The data in the memos without duplicates were then used by N.K. to create a first draft with lifestyle recommendations. This first draft was, subsequently, reviewed by all authors, and involved patients (Connie van Bemmel, Deborah Boeken, and Roelie Pomstra) and health care professionals (Jonny de Swart, Odette van Acker, Maud Driessen, Mirjam van der Laan-Blanken, and Bart Thoolen). The reviewers were asked to provide feedback on the fit of the lifestyle recommendations (to which extent the lifestyle recommendations fit with the raw research data) and clarity of the lifestyle recommendations (to which extent are the lifestyle recommendations clear).
8. Final draft	The feedback was, subsequently, used by N.K. and H.v.Z. to create a final draft with lifestyle advice. The final draft was translated into the English language with a standardized forward–backward procedure by a third party.

**Table 2:** Thematic framework for indexing all lifestyle recommendations for patients with thoracic aortic surgery

Theme	Explanation
Behavioral change	Instructions to act differently. Behavioral change is a complex process that involves letting go of old behaviors and learning new ones.
Body weight	Instructions to reduce, maintain, or increase your body weight. These lifestyle recommendations may be related to kilograms of body weight or body mass index.
Nutrition	Instructions for ingesting food. These lifestyle recommendations usually apply to the human behavior of eating.
Cessation of alcohol and drug use a	Instructions for the use of narcotics. This includes the use of alcohol, sleeping pills and tranquilizers, opium, morphine, heroin, and Gamma-hydroxybutyrate (GHB). Medication use falls outside the scope of this study.
Cessation of smoking	Instructions for cessation of smoking focus on the cessation itself and the prevention of relapse into old smoking behavior.
Wound healing a	Instructions for wound healing refer to activities to support the replacement of damaged tissue by newly produced tissue. Wound healing is necessary because, in thoracic aortic surgery, a wound is made on the sternum or the side of the thorax.
Sedentary behavior and physical activity	Instructions for sedentary behavior and physical activity concern patients resting, moving, acting, and performing within culturally specific spaces and contexts. These lifestyle recommendations may be related to lying in bed, sitting, walking, or exercising.
Mental well-being a	Instructions about the thoughts and feelings of patients and how they might be able to cope with the ups and downs before and after thoracic aortic surgery.
Family and close relatives a	Instructions on how to behave with members of a patient's family who are most directly related, for example, partners, parents, and siblings.

a Themes added to the thematic framework after indexing to include all relevant research data.

### Patient and Public Involvement

Patients from the Stichting Aortadissectie Nederland (the Netherlands Aortic Dissection Foundation) were involved in the conceptualization and reporting of this study. Specifically, Connie van Bemmel, Deborah Boeken, and Roelie Pomstra provided feedback on the study's conceptualization, first draft, and final draft of the lifestyle recommendations.

## Results

### Data

**Fig. 1:**
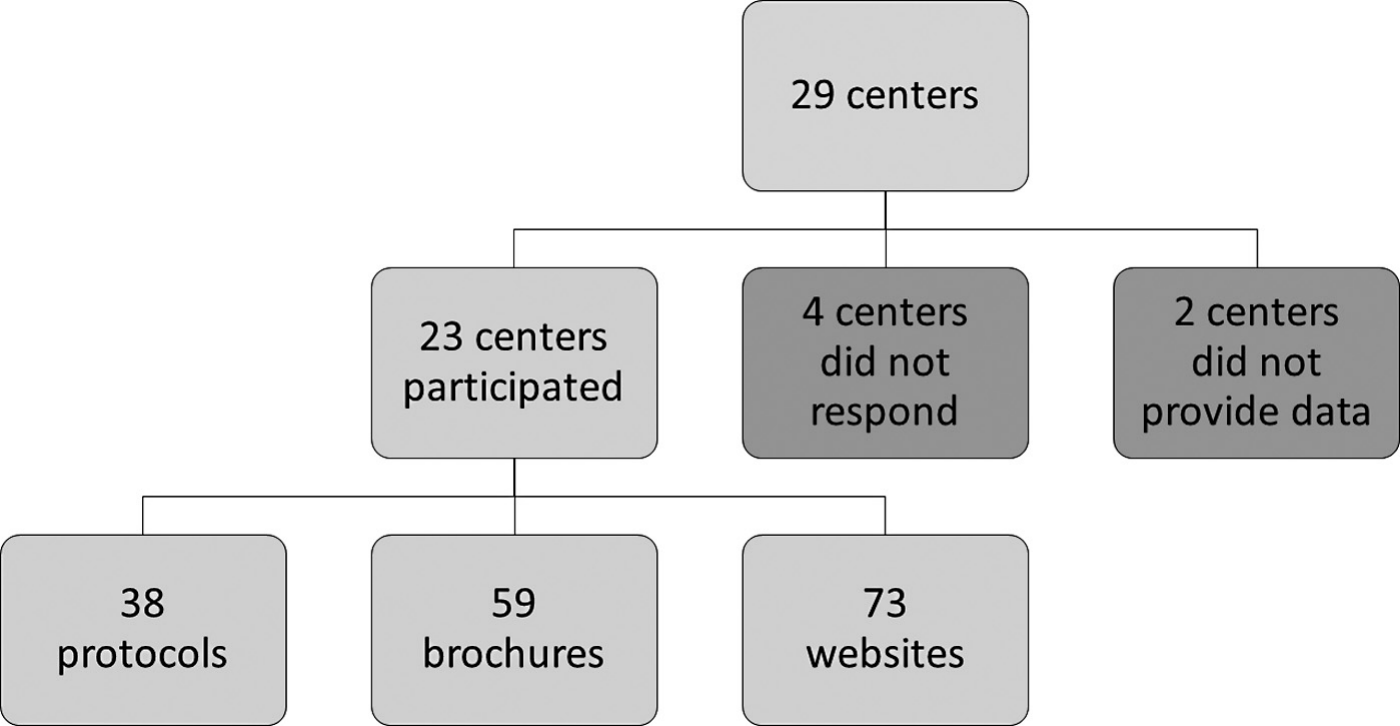
Flow diagram showing numbers of participating centers and documents obtained.

In total, 16 cardiac surgery centers (100%) and 9 cardiac rehabilitation centers (69%) responded to the study invitation ( Fig. 1 ). Four cardiac surgery centers did not respond. Two cardiac rehabilitation centers responded that they had no documents for patients before and after thoracic aortic surgery. In total, the centers shared 59 brochures, 73 website texts, and 38 health care protocols. Indexing showed that 40 brochures (68% of the brochures, 52% of the responding centers), 19 website texts (26% of the website texts, 52% of the responding centers), and 13 health care protocols (34% of the health care protocols, 20% of the responding centers) contained, in total, 414 lifestyle recommendations for patients before and after thoracic aortic surgery. Mapping revealed 31 lifestyle recommendations related to behavioral change, 12 lifestyle recommendations related to body weight, 30 lifestyle recommendations related to nutrition, 12 lifestyle recommendations related to cessation of alcohol and drug use, 16 lifestyle recommendations related to cessation of smoking, 38 lifestyle recommendations related to wound healing, 223 lifestyle recommendations related to sedentary behavior and physical activity, 33 lifestyle recommendations related to mental well-being, and 19 lifestyle recommendations related to family and close relatives. Removal of duplicates and processing of feedback resulted in a total of 52 clear lifestyle recommendations with nine themes. A total of 11 lifestyle recommendations always apply (before and after thoracic aortic surgery), 5 apply to the period before surgery, and 36 apply to the period after surgery.

### Lifestyle Recommendations

The final drafts are provided in Supplementary Table S1 (available in the online version only) and Supplementary Material S1 (available in the online version only). The first draft with lifestyle recommendations for patients after thoracic aortic surgery is provided in Supplementary Material S2 (available in the online version only). The nine themes and lifestyle recommendations are briefly described in the text below.

Regarding behavioral change , lifestyle recommendations focus on engaging in healthy behaviors, such as healthy nutrition, and stopping unhealthy behaviors, like smoking. Furthermore, there are lifestyle recommendations not to lose body weight before surgery, to weigh yourself daily after surgery, and to aspire to a healthy weight after surgery. Moreover, there are lifestyle recommendations concerning healthy nutrition : eat high-fiber, low-salt, oily fish once a week, and plenty of fruits and vegetables. In addition, it is recommended to limit or cessation of alcohol and drug use if possible. The recommendation that “alcohol should be used in moderation, because of healthy effects on the heart muscle” has been removed as a result of a disagreement between authors and feedback from health care professionals that arose in the first draft. In another theme, recommendations are constructed about cessation of smoking : do not smoke or at least quit smoking 8 weeks before surgery. The recommendations on wound healing are divided into three subthemes: *general recommendations* , specific recommendations after *sternotomy* , and specific recommendations after *lateral thoracotomy* . The lifestyle recommendations for recovery after sternotomy aim to improve bone healing, whereas the recommendations after lateral thoracotomy aim to reduce stretch on superficial surgical wounds. The recommendations on sedentary behavior and physical activity are divided into five subthemes: *before surgery* , *activities* , *resting* , *traffic* , and *holidays* . The recommendations to “stay indoors for a week after hospital discharge as your resistance is not yet optimal” and “listen to your own body to determine the number of activities” were removed as a result of feedback by patients and health care professionals on the first draft. Moreover, there are lifestyle recommendations for improved mental well-being : try to relax; seek help with persistent fear or insecurity, gloomy or sad feelings, tension, or stress; take time to restore loss of concentration, fatigue, and labile emotional functioning. Finally, the theme of family and close relatives concerns lifestyle recommendations to arrange social support for the first 10 days after surgery.

## Discussion

### Main Findings

To the best of our knowledge, this is the first study that presents a complete overview of clear lifestyle recommendations for patients before and after thoracic aortic surgery. These lifestyle recommendations can be used before surgery to inform patients and manage outcome expectations. After surgery, these lifestyle recommendations might play an important role in promoting health and secondary prevention. It is important to maintain a healthy weight, eat healthily, drink no alcohol, and cease smoking. Furthermore, it is crucial to manage the wound well, with specific recommendations for patients who have undergone a sternotomy and patients who have undergone a lateral thoracotomy. To promote physical health, it is key to follow lifestyle recommendations for physical activity and sedentary behavior. The lifestyle recommendations on mental well-being and dealing with family and close relatives are crucial to improving mental health and have not been described, to our knowledge, in earlier guidelines.

Some lifestyle recommendations led to discussions, such as sleeping on the back in the first 6 weeks after a sternotomy, not driving a car in the first 6 weeks after surgery, and not flying in the first 4 to 12 weeks after surgery. These recommendations are included in the final draft as a result of the document research, however, there is still ongoing debate about them in recent literature. 16 El-Ansary et al 17 propose comfortable sleeping in any position after a sternotomy in their evidence- and perspective based on the minimal risk of sternal complications. Driving limitations are still supported with evidence suggesting limited neurocognitive functioning in the first 4 to 12 weeks after surgery. 18 Smith et al 19 state that flying should be safe at least 10 days after surgery for patients without complications (i.e., patients without arrhythmia, pleural effusion, wound infection, anemia, rhythm disturbances, and left ventricular dysfunction). We recognize that the Dutch recommendations are somewhat restrictive compared to the American recommendations.

There is a plethora of research exploring lifestyle interventions in people with obesity, and healthy populations. 20 21 In patients with coronary heart disease, the following lifestyle themes are presented for blood pressure management: sodium reduction, weight loss, healthy nutrition, alcohol reduction, cessation of smoking, and increased physical activity. 22 Noteworthy in this study is the specific attention to sodium, where the authors recommend an intake of <2,400 mg per day. 23 When looking at the literature for patients with surgery, our lifestyle themes align with patients following stoma formation surgery where smoking, alcohol consumption, nutrition (diet), and physical activity are well-known lifestyle themes. 24

Furthermore, the involved patients and consulted health care professionals did mention some lifestyle recommendations that were missing in their opinion. For example, they advised not arranging a bed downstairs after surgery and promoted stair walking, because this contributed to improving stamina and strength. Additional nutritional recommendations consisted of sufficient calcium and vitamin D intake for patients over 50 years old (particularly women) and adequate drinking (particularly for patients over 70 years old). Furthermore, they emphasized not using drugs and avoiding stressful activities that could be stopped while doing (specifically skydiving and riding roller coasters).

The overview with 52 clear lifestyle recommendations for patients before and after thoracic aortic surgery is immediately applicable in daily practice. However, it does not seem appropriate to hand over a list of lifestyle recommendations without any support. Rehabilitation professionals are well-suited health care professionals to support patients in return-to-sport and long-term lifestyle adaptation. 25 In addition, they are able to tailor recommendations based on, amongst others, etiology (degenerative vs. hereditary disease), aortic dilatation, and sports intensity. Physical therapists and dieticians might support patients on a day-to-day basis with explicit attention to behavioral change, body weight, nutrition, sedentary behavior, and physical activity.

### Strengths and Limitations

The first strength concerns the methods of data collection. The data have been collected through official information channels (i.e., staff secretary and health care professionals) to ensure the authenticity of all documents. Second, the trustworthiness of study methods was ensured through (1) two researchers independently analyzing the data (N.K. and H.v.Z.) to enhance credibility, and (2) the use of Atlas.ti to enhance confirmability. Third, the final draft of lifestyle recommendations for patients before and after thoracic aortic surgery consists of many instructions on *what to do* (recommendations) instead of *what not to do* (restrictions). The positive nature of lifestyle recommendations might lead to better use in daily practice and less distress and anxiety for patients.

The first limitation concerns the handling of disagreements on lifestyle recommendations between different documents. Disagreements have been solved by consensus of authors and consultation of involved health care professionals. Their feedback was used to create a final draft of clear lifestyle recommendations for patients before and after thoracic aortic surgery. Another scientific method for dealing with disagreement would be a RAND Delphi study, in which a team of experts would be asked to reach a consensus. Second, data in this study were extracted from brochures, website texts, and health care protocols. Unfortunately, our sources, yielded no references to scientific literature since references would have increased the credibility of the recommendations. Therefore, we conclude that the recommendations are based on general knowledge, common sense, or surgical principles. As a next step, the recommendations should be studied and tested according to evidence-based principles. Third, little distinction is made between lifestyle recommendations for people with or without heritable thoracic aortic diseases. Different lifestyle recommendations might be expected due to the pathological mechanism and risk for secondary complaints. However, we found no specific lifestyle recommendations for this important population.

## Conclusion

We present 52 clear lifestyle recommendations for patients before and after thoracic aortic surgery. This is the first step towards creating evidence-based lifestyle recommendations because follow-up research is needed on which lifestyle recommendations are necessary and evidence-based. There is a lot of information available on this topic. However, all information has now been summarized for the first time into nine major lifestyle themes: (1) behavioral change, (2) body weight, (3) nutrition, (4) cessation of alcohol and drug use, (5) cessation of smoking, (6) wound healing, (7) sedentary behavior and physical activity, (8) mental well-being, and (9) family and close relatives. Providing clear, necessary, and effective lifestyle recommendations for patients before and after thoracic aortic surgery is difficult because lifestyle recommendations are individual to each person. However, the current overview of lifestyle recommendations provides a basis on which substantiated individual choices can be made.
