# Supplementary material for: Lifestyle Recommendations for Patients Before and After Thoracic Aortic Surgery: A Framework Analysis
Source: Aorta (Stamford). 2024 Oct 29;12(1):1–7. doi: 10.1055/s-0044-1791668 (PMC11606666; doi:10.1055/s-0044-1791668)
Supplement: Supplementary file 1 — Supplementary Material [file 10-1055-s-0044-1791668-s230012.pdf]

Supplementary Table S1 Final draft lifestyle recommendations for patients with thoracic aortic surgery (short version)

	Behavioral change	Period
1	We always recommend following lifestyle recommendations. This means that you will receive recommendations on how to adapt your life, for example: lose weight, eat healthily, limit alcohol consumption, stop smoking, exercise sufficiently, avoid stress, and interact with loved ones.	Always
2	We recommend participating in cardiac rehabilitation.	After surgery
	**Body weight**	
3	We recommend not losing weight in the period before surgery.	Before surgery
4	We recommend weighing yourself daily for the first 2 weeks after surgery.	After surgery
5	We recommend striving for a healthy body weight in the period after surgery.	After surgery
	**Nutrition**	
6	We recommend eating healthily. This helps to stay healthy or recover better.	Always
7	We recommend eating high-fiber foods. This is important for bowel movement.	Always
8	We recommend a low-salt diet. This helps to prevent fluid retention and improves blood pressure.	Always
9	We recommend eating fish once a week, preferably oily fish.	Always
10	We recommend eating two pieces of fruit and 250 g of vegetables a day.	Always
	**Cessation of alcohol and drug use**	
11	We recommend not drinking alcohol for at least 2 weeks before surgery.	Before surgery
12	We recommend not drinking alcohol. It is possible to have up to one glass of alcohol per day after surgery but preferably not every day.	After surgery
13	We recommend informing the thrombosis service if you drink two or more glasses per day.	After surgery
	**Cessation of smoking**	
14	We recommend that you stop smoking for at least 8 weeks before surgery.	Before surgery
15	We recommend quitting smoking.	Always
	**Wound healing—in general**	
16	We recommend caution if exposing the wound to sunlight.	After surgery
17	We recommend against bathing, sunbathing, swimming, and saunas for the first 6 weeks after surgery.	After surgery
18	We recommend contacting your surgeon immediately with any signs of wound infection (redness, warmth, fluid, or pus from the wound and/or fever).	After surgery
	**Wound healing—sternum**	
19	We recommend not lifting heavy things, not walking the dog, not cycling (an exercise bike is allowed), not swimming, and not doing heavy household chores in the first 6 weeks after surgery.	After surgery
20	We recommend not lifting more than 5 kg in the first 6 weeks. Always try to lift with two arms and make symmetrical movements when lifting, turning on your side, or getting out of bed.	After surgery
21	We recommend women with heavy breasts wear a non-wire bra day and night in the first 6 weeks after surgery.	After surgery
22	We recommend sleeping on your back for the first 6 weeks.	After surgery
	**Wound healing—lateral chest**	
23	We recommend gently moving your arms and shoulders, despite any pain you may experience.	After surgery
24	If stitches have not all been removed, we recommend that you have them removed by your doctor after 10 to 14 days.	After surgery
	**Sedentary behavior and physical activity—before surgery**	
25	We recommend being physically active as much as possible before heart surgery, as appropriate for your situation.	Before surgery
	**Sedentary behavior and physical activity—activities**	
26	We recommend doing more and more by yourself, such as getting out of bed, dressing, and showering.	After surgery
27	We recommend going outside, weather permitting.	After surgery

 © 2024. The Author(s).

Supplementary Table S1 (*Continued*)

	Behavioral change	Period
28	We recommend going for walks during the first 6 weeks after surgery.	After surgery
29	We recommend wearing comfortable clothing when walking or exercising and, if necessary, using a (walking) aid.	After surgery
30	We recommend doing light household chores such as doing the dishes, cooking, and dusting in the first 6 weeks after surgery.	After surgery
31	It is possible, when you and your partner are ready, to have sexual intercourse again.	After surgery
32	We recommend not doing any heavy household chores during the first 6 weeks, such as vacuuming, mopping, lifting children, cleaning windows, cleaning the toilet or bathroom, walking the dog, and lifting large grocery bags.	After surgery
33	We recommend exercising at least half an hour a day, 5 days a week, at moderate intensity from 6 weeks after surgery onward.	After surgery
34	It is possible to start playing sports after your surgery.	After surgery
35	We recommend resuming work between 3 and 6 months after surgery, depending on the progress of cardiac rehabilitation, job activities, and your condition.	After surgery
	**Sedentary behavior and physical activity—resting**	
36	We recommend developing a daily routine and building up a day and night rhythm.	Always
37	We recommend exercising and increasingly doing so but be careful not to overexert yourself.	Always
38	We recommend using the bedroom for sleep and lovemaking only.	Always
	**Sedentary behavior and physical activity—traffic**	
39	We recommend against cycling or driving a car for the first 6 weeks after surgery.	After surgery
40	We recommend slowly building up walking on a home trainer and cycling on an exercise bike (without resistance) in the first 6 weeks.	After surgery
	**Sedentary behavior and physical activity—holiday**	
41	It is possible to go on holiday as of 4 weeks after surgery, provided you are feeling well.	After surgery
42	It is possible to travel by air after your surgery. You are allowed to fly again between 4 and 12 weeks after surgery.	After surgery
43	We recommend taking your medical history and list of medications with you when you travel.	After surgery
	**Mental well-being**	
44	We recommend consciously relaxing regularly.	Always
45	It is possible that you might feel anxious or insecure after your surgery. If this persists for 6 weeks and affects your daily life, we recommend you seek help.	After surgery
46	It is possible that you might feel down and/or sad after your surgery. If this persists for 6 weeks and affects your daily life, we recommend you seek help.	After surgery
47	It is possible that you might experience tension after your surgery. If this persists for 6 weeks and affects your daily life, we recommend you seek help.	After surgery
48	It is possible that your ability to concentrate might be less than normal after your surgery. Allow yourself time to recover your ability to concentrate.	After surgery
49	It is possible that you might feel tired or emotionally unstable after surgery. We recommend not worrying too much: it is all part of the process, and it will pass.	After surgery
	**Family and close relatives**	
50	We recommend that you think about whether you will have adequate care after surgery before you are admitted.	Before surgery
51	We recommend not to be home alone during the day (most of the time) or at night for the first 10 days.	After surgery
52	We recommend that you do not receive too many visitors for the first 6 weeks after surgery.	After surgery

 © 2024. The Author(s).

Supplementary Material S1. Final draft lifestyle recommendations for patients with thoracic aortic surgery in Dutch (full version)

Gedragsverandering

We adviseren altijd leefstijladviezen op te volgen. Dit betekent dat je advies krijgt om bepaalde zaken van je leven aan te passen, zoals bijvoorbeeld: stoppen met roken, afvallen, gezond eten, het verminderen van zoutinname, voldoende bewegen, beperkt alcoholgebruik en het voorkomen van stress. Het is belangrijk dat je de adviezen goed opvolgt, om gezond te blijven en gezondheidsproblemen te voorkomen.

We adviseren deel te nemen aan hartrevalidatie. Hartrevalidatie kan je helpen om, onder begeleiding van een medisch team, een betere conditie te ontwikkelen en zelfvertrouwen op te bouwen. Tijdens een hartrevalidatieprogramma: leer je je (lichamelijke) grenzen kennen en verleggen; doe je aan sport en spel; leer je beter te ontspannen; krijg je begeleiding in de sociale en psychische kant van het leven na aortachirurgie; krijg je voorlichting over risicofactoren; en krijg je adviezen over voeding en bewegen. Je kunt het hartrevalidatieprogramma gebruiken om een plan te maken voor de toekomst en je leven gezond(er) in te richten. De hartrevalidatie start bij voorkeur vier tot zes weken na operatie in het ziekenhuis van jouw behandelend arts.

Lichaamsgewicht

We adviseren in de periode voor de operatie niet af te vallen. Je lichaam heeft na de operatie alle energie nodig om te herstellen. Een dieet om af te vallen, raden we daarom in de periode voor de operatie en in de eerste zes weken na de operatie af.

We adviseren dat je je de eerste twee weken na de operatie dagelijks weegt, omdat je lichaam na de operatie vocht kan vasthouden. Doe dit steeds op dezelfde plek, dezelfde weegschaal en hetzelfde tijdstip, bijvoorbeeld voor het ontbijt en nadat je naar het toilet bent geweest. Als je meer dan twee kilo aankomt in drie dagen, neem dan contact op met je huisarts.

We adviseren in de periode na de ziekenhuisopname een gezond lichaamsgewicht na te streven. Voor iedereen geldt dat overgewicht slecht voor de gezondheid is. Afvallen, al is het een paar kilo, levert dan winst voor je gezondheid op. Een gezond lichaamsgewicht controleer je op twee manieren: (1) een BMI <25 is gezond, een BMI tussen 25–30 is licht overgewicht en een BMI >30 is overgewicht, en (2) een middelomtrek van <88 cm voor vrouwen of <102 cm voor mannen is gezond.

Voeding

We adviseren gezond te eten, omdat gezond eten helpt om gezond te blijven of beter te herstellen. Onder gezond eten wordt verstaan dat je bewuste en gezonde keuzes maakt wat betreft de voedingsmiddelen die je eet. Als basis voor gezond eten kun je de Schijf van Vijf gebruiken die is samengesteld door het Voedingscentrum in Den Haag. Het advies is (1) eet elke dag 250 gram groente, (2) eet elke dag twee porties fruit, (3) eet bij voorkeur volkoren brood, graanproducten en aardappelen, (4) varieer met vis, peulvruchten, vlees en ei, (5) eet elke dag een handje vol noten, (6) neem elke dag zuivel, melk, yoghurt of kaas, (7) smeer en bak met onverzadigde vetten, en (8) drink dranken zonder suiker. Na de operatie hebben sommige mensen weinig tot geen trek. Juist dan is het belangrijk om goede voedingsstoffen binnen te krijgen. Als je moeite hebt met gezond eten, neem dan contact op met je huisarts of diëtist.

We adviseren vezelrijk te eten. Dit is belangrijk voor de stoelgang. Eet bij voorkeur volkorenbrood in plaats van wit brood.

We adviseren zoutarm te eten. Je lichaam heeft namelijk de neiging vocht vast te houden. Zoutarm eten kan dit tegengaan. Verder draagt zoutarm eten bij aan een betere bloeddruk. Tips voor minder zoute maaltijden zijn: (1) Gebruik vleeswaren en kaas met minder zout. (2) Voeg zo min mogelijk zout toe aan de maaltijd. (3) Neem minder kant-en-klaar producten. (4) Beperk het gebruik van zoute smaakmakers, zoals: bouillonblokjes, sojasaus, sambal en kruidenmixen. (5) Beperk het gebruik van gemarineerd, gekruid of gepaneerd vlees of vis.

We adviseren je om één keer per week vis te eten, bij voorkeur vette vis. Vooral vette vis bevat goede vetzuren. Voorbeelden van vette vis zijn: zalm, haring, makreel, sprot en heilbot. Het eten van vette vis heeft een gezonde invloed op de bloedvaten.

We adviseren je om per dag twee stuks fruit en 250 gram groente te eten. Het eten van groente en fruit eten heeft een gezonde invloed op de bloedvaten.

Alcohol en Drugs Gebruik

We adviseren vóór de operatie tenminste 2 weken te stoppen met het gebruik van alcohol. Zo voorkom je dat e klachten kunt krijgen door het stoppen. Deze kun je nog krijgen 10 tot 12 dagen na het stoppen. Stoppen met het gebruik van alcohol doe je voor jezelf, om de kans op schade aan je gezondheid zo klein mogelijk te maken.

We adviseren geen alcohol te drinken. Het is mogelijk na de operatie één glas alcohol per dag te drinken, maar bij voorkeur niet elke dag. Alcohol wordt, net als veel medicijnen, door de lever afgebroken. Alcohol kan hierdoor de werking van medicijnen beïnvloeden. Verder heeft alcohol een invloed op je denkvermogen en wekt alcoholverslaving in de hand. Daarnaast verhoogt alcohol de bloeddruk en bevat het veel calorieën. Als je alcoholhoudende drank neemt, dan is het beter bij het eten te nemen in plaats van tussendoor.

We adviseren bij gebruik van twee of meer glazen per dag dit door te geven aan de trombosedienst. Alcohol kan de bloed verdunnende werking van medicijnen namelijk versterken.

Stoppen Met Roken

We adviseren je te stoppen met roken. Roken is slecht voor je hart en bloedvaten. Door roken komen er stoffen in het bloed

 © 2024. The Author(s).

die schadelijk zijn voor de wanden van de bloedvaten. Stoppen met roken levert al na enkele weken gezondheidsvoordelen op, zoals meer lucht, betere geur en smaak, gezondere huidskleur, minder hoesten, warmere handen en voeten en je fitter voelen.

We adviseren je tenminste acht weken vóór de operatie te stoppen met roken. Mensen die vóór een operatie roken hebben meer kans op longproblemen en wondinfecties na een operatie.

Wondgenezing

We adviseren bij tekenen van een wondinfectie (roodheid, warmte, vocht of pus uit de wond en/of koorts) direct contact op te nemen met jouw behandelend arts.

We adviseren je voorzichtig te zijn met zonlicht op de wond. Smeer het litteken in met een zonnebrandmiddel met hoge beschermingsfactor of een sunblock. Littekenweefsel bevat namelijk weinig of geen pigment en kan snel verbranden.

We adviseren de eerste weken na de operatie niet in bad te gaan, niet onder de zonnebank te gaan, niet te zwemmen en niet de sauna te bezoeken. Dit heeft als doel te voorkomen dat de wond week wordt. Je mag wel elke dag douchen. Gebruik de eerste dagen geen zeep bij de wonden en dep de wond en het gebied rond de wond droog. Gebruik geen poeder of zalf op de wond. Dit maakt de kans op een infectie groter.

Wanneer je een operatie via het borstbeen hebt gehad gelden de volgende adviezen:

- We adviseren de eerste zes weken na de operatie geen zware dingen te tillen, niet de hond uit laten, niet te fietsen (hometrainer mag wel), niet te zwemmen en geen zware huishoudelijke werkzaamheden verrichten. Dit zijn handelingen waarbij het borstbeen kwetsbaar is. Het duurt gemiddeld zes weken voordat de borstbeenhelften weer stevig aan elkaar vastgegroeid zijn. Na zes weken is de genezing van het borstbeen zo ver gevorderd dat de meeste dagelijkse handelingen weer verricht kunnen worden.
- We adviseren de eerste zes weken niet meer dan vijf kilo te tillen en doe dit met twee armen tegelijk. Probeer steeds symmetrische bewegingen met beide armen te maken wanneer je tilt, op de zij draait of uit bed komt. Het duurt gemiddeld zes weken voordat de borstbeenhelften weer stevig aan elkaar vastgegroeid zijn. Na zes weken is de genezing van het borstbeen zo ver gevorderd dat je meer mag tillen en asymmetrisch kan bewegen.
- We adviseren vrouwen met zware borsten om dag en nacht een beha zonder beugels te dragen. Daardoor staat er weinig spanning op het litteken en is de wond minder pijnlijk. Ook wordt dan het litteken minder breed en dus mooier.
- We adviseren je om de eerste zes weken op je rug te slapen. Als je hierdoor niet kunt slapen, mag je wat op de zij draaien. Dit mag alleen zolang je hier geen pijn bij hebt.

Wanneer je een operatie via de zijkant van je bovenlichaam hebt gehad gelden de volgende adviezen:

- We adviseren je om rustig te bewegen met je armen en schouders, ondanks eventuele pijnklachten. De wond zal de eerste dagen na de operatie nog wat pijnlijk zijn. Rustig bewegen, een goede houding (recht overeind zitten en lopen) en voldoende pijnstilling helpen tegen de pijn.
- We adviseren je om, indien nog niet alle hechtingen verwijderd zijn, deze na 10 tot 14 dagen te laten verwijderen door de huisarts. Hierdoor herstelt de wond sneller en beter.

Sedentaire Leefstijl en Lichamelijke Activiteit

We adviseren regelmaat in je dag aan te brengen en een dag- en nachtritme op te bouwen. Dit is een goede manier om jouw lichaam de rust te geven die het nodig heeft om te herstellen. Verspreid activiteiten over de dag en neem genoeg pauzes. Het kan fijn zijn tussen de middag maximaal één uur te rusten. Blijf niet langer dan acht uur in bed liggen, ook niet in het weekend. Lang in bed liggen verstoort de biologische klok.

We adviseren om te bewegen en dit uit te breiden, maar let erop dat je niet gaat overbelasten. Overbelasten kan het herstel namelijk vertragen. Verschijnselen van overbelasting zijn: pijn of druk op de borst; kortademigheid of abnormale vermoeidheid die niet in verhouding staat tot het bewegen; toename van vocht in het gehele lichaam, armen of benen; een onregelmatige hartslag of hartkloppingen; overmatig zweten, misselijkheid of duizeligheid. Nemen deze klachten niet af na rust, dan adviseren we contact op te nemen met jouw huisarts of specialist.

We adviseren de slaapkamer alleen voor slaap en de liefde te gebruiken. Een goed bed is natuurlijk belangrijk voor goede slaap. Zorg er dus in de eerste plaats voor dat je een prettig liggend matras, een goed kussen en fijn beddengoed hebt. Daarna is het belangrijk dat de kamer lekker donker is voordat je gaat slapen. Zorg er ook voor dat je niet te veel wordt afgeleid door omgevingsgeluiden. Oordoppen kunnen helpen! Zet de wekker uit je directe zicht. Het scheelt onrust als je niet steeds kunt zien hoe laat het is. Houd de kamer en je lichaam koel en slaap in iets luchtigs.

We adviseren vóór de hartoperatie zoveel mogelijk te bewegen, passend bij jouw situatie. Bewegen is namelijk goed voor je conditie. Kies een activiteit die niet te belastend is en die je een langere tijd kunt volhouden. Denk hierbij aan wandelen of fietsen. We adviseren niet te trainen zoals rennen, basketballen of gewichtheffen, omdat hierbij de bloeddruk gevaarlijk hoog kan worden. Het is wel mogelijk om op conditie te blijven door te zwemmen, fietsen of traplopen. Let hierbij op dat je hele zinnen kan blijven praten en laat je hartslag en bloeddruk controleren. Twijfel je of je een bepaalde activiteit mag doen? Neem dan contact op met jouw arts of fysiotherapeut.

We adviseren je na de operatie steeds meer zelf te doen, zoals uit bed komen, aankleden en douchen. Je kunt je na het douchen de wond voorzichtig droogdeppen. Het kan prettig zijn om een douchekruk te gebruiken om tussendoor uit te rusten. Je kunt de eerste zes weken beter geen bad nemen, dat maakt de wondranden week.

We adviseren licht huishoudelijk werk te doen, zoals afwassen, koken en stoffen. Dit is gezond en helpt rustig in

beweging te komen. Verdeel je taken over de dag, niet alles hoeft in één keer af.

We adviseren de eerste zes weken geen zwaar huishoudelijk werk te doen, zoals stofzuigen, beddengoed verschonen, dweilen, kinderen tillen, ramen zemen, toilet of badkamer schoonmaken, hond uitlaten en de grotere boodschappen tillen. Dit omdat dit activiteiten zijn waarbij je beide armen afwisselend gebruikt. Bij deze activiteiten is de druk op de wond groot, waardoor er een kans bestaat dat de wond moeizaam herstelt.

We adviseren je de eerste zes weken na de operatie te wandelen. Wandelen is een gezonde vorm van bewegen. Probeer dagelijks twee keer te wandelen en dit steeds met een aantal minuten uit te breiden. Je legt een goede afstand af als je hele zinnen kan blijven praten bij een prettig tempo. Denk er wel aan dat je ook weer terug moet. Na maximaal een half uur rust moet je je weer fit voelen.

We adviseren je vanaf zes weken na de operatie minimaal een half uur per dag, op minstens vijf dagen van de week, matig intensief te bewegen. Dit hoeft niet meteen sporten te betekenen. Bewegen is ook wandelen (tussen de 4 en 5 kilometer per uur), fietsen (ongeveer 15 kilometer per uur), fietsen op een hometrainer of traplopen. Bewegen houdt je fit en speelt een grote rol in het voorkomen van ziekten en aandoeningen. Ook heeft bewegen een gezonde invloed op je geestelijke gezondheid. Afspreken om regelmatig samen met iemand te gaan wandelen of fietsen kan helpen om van een goed voornemen een goede gewoonte te maken.

We adviseren je naar buiten te gaan, als het weer het toelaat. Buiten bewegen verkleint direct de kans op hart-en vaatziekten en draagt bij aan een goede geestelijke gezondheid.

We adviseren je gemakkelijk zittende kleding aan te doen bij het bewegen en, indien nodig, een (loop)hulpmiddel te gebruiken. Trek schoenen aan die stevig aan jouw voeten blijven zitten en een goed profiel hebben. Als je een verminderde balans hebt is het belangrijk een hulpmiddel te gebruiken, zoals een rollator. Als je voor de eerste keer na je ontslag buiten gaat bewegen, is het verstandig om dit samen met iemand te doen. Mogelijk is je stabiliteit nog niet optimaal en de indrukken die je buiten opdoet of de ongelijke ondergrond kunnen je wellicht nog extra uit balans brengen.

We adviseren tussen drie en zes maanden na de operatie werk te hervatten, afhankelijk van de aard van de werkzaamheden en jouw conditie. Je voelt over het algemeen zelf het best wanneer je weer aan werken toe bent. Bespreek werkhervatting in een vroeg stadium met je partner, werkgever, bedrijfsarts, huisarts en cardioloog. Vaak is het verstandig te beginnen met halve dagen en dit langzaam uit te breiden.

Het is mogelijk om na je operatie te gaan sporten. Dit is afhankelijk van je persoonlijke situatie. Wat is er precies gebeurd, wat is de behandeling geweest en welke sport(en) wil je gaan beoefenen? In de meeste gevallen kun je tot bepaalde hoogte oude bezigheden weer oppakken. Sport is namelijk goed voor je conditie. Kies een activiteit die niet te belastend is en die je een langere tijd kunt volhouden. Denk hierbij aan wandelen of fietsen. We adviseren niet te rennen, spinning, judoën, boksen, basketballen, rugbyen, handballen of gewichtheffen, omdat hierbij de bloeddruk gevaarlijk hoog kan worden. Het is wel mogelijk om te zwemmen, dansen, tennissen, badmintonnen, biljarten, voetballen, volleyballen, golfen en traplopen. Let hierbij op dat je hele zinnen kan blijven praten en laat je hartslag en bloeddruk controleren. Het kan verstandig zijn op recreatief niveau te sporten in plaats van competitie niveau. Overleg dit met je arts of fysiotherapeut. Bergwandelen (boven 500 m) en diepzeeduiken zijn ook sporten die je het best kunt bespreken met je behandelaar in verband met hoge drukverschillen. Het is belangrijk om rustig op te bouwen, piekbelasting te voorkomen en overbelasting te herkennen.

Het is mogelijk, wanneer jij en je partner daar aan toe zijn, weer te vrijen. In verband met een verhoogde bloeddruk tijdens inspanning en een nog niet helemaal genezen operatiewond is het verstandig dat je de eerste zes weken voorzichtig aan doet bij het vrijen. Vrijen is ongeveer even belastend als twee trappen lopen. Als je dat zonder echte klachten kunt doen, is er geen bezwaar tegen. Het is heel normaal als je na een ziekenhuisopname geen zin in vrijen hebt. Voor de eerste keer vrijen na een ziekenhuisopname is spannend en valt soms tegen. Wees niet te snel teleurgesteld als het niet verloopt zoals je het wenst. Laat je niet weerhouden om het op een ander moment opnieuw te proberen.

Veel mensen hebben de eerste weken na een ziekenhuisopname niet zoveel belangstelling voor seks. Overleg met jouw behandelend arts als dit voor lange tijd aanblijft en onprettig aanvoelt, zodat er naar een oplossing gezocht kan worden..

Het is mogelijk om vanaf vier weken na de operatie op vakantie gaan, mits je je goed voelt. Het advies is de eerste vier weken nog niet op vakantie te gaan, zodat je bij eventuele gezondheidsproblemen direct contact kunt opnemen met je behandelend arts. We adviseren rustig aan te doen op vakantie. Of een reis naar de bergen of andere inspannende bestemming verstandig is, kun je het beste bespreken bij je huisarts, want dat is per persoon verschillend.

Het is mogelijk om te vliegen na je operatie. Het moment dat je weer mag vliegen ligt tussen de vier en twaalf weken na de operatie. Dit is afhankelijk van de grootte van de operatie, het herstel van de operatiewond en je conditie. Overleg daarom altijd met je behandelend arts.

We adviseren je om jouw ziektegeschiedenis en geneesmiddelenoverzicht mee te nemen op reis. Deze kun je bij jouw behandelend arts of huisarts opvragen. Het kan verstandig zijn eigen medicijnen en een medicijnenpaspoort mee te nemen, omdat niet alle medicijnen beschikbaar zijn in het buitenland. Als je onder controle van de trombosedienst bent, kun je daar bespreken waar je terecht kunt voor controle op het gebruik van antistollingsmiddelen

We adviseren de eerste zes weken na de operatie niet zelf te fietsen of auto te rijden. Door de aandoening, de operatie en medicijnen is het mogelijk dat je concentratievermogen de eerste zes weken verminderd is. Hierdoor zijn situaties in het verkeer soms lastig in te schatten. Daarnaast is de

 © 2024. The Author(s).

operatiewond van borstbeen of borstholte pas na zes weken voldoende genezen. Het is daarom beter om in deze periode geen fiets of auto te besturen in verband met de operatiewond op borstbeen of borstholte. Doe in de auto als bijrijder altijd een gordel om! Ook verzekeringstechnisch ben je dit verplicht.

We adviseren de eerste zes weken het wandelen en fietsen op een hometrainer (met minimale weerstand) rustig op te bouwen. Dit is goed voor je conditie.

Mentaal Welzijn

We adviseren je bewust en regelmatig te ontspannen. Ontspanning heeft een positieve invloed op de hartslag en de bloeddruk.

Het is mogelijk dat je angst en/of onzeker bent na je operatie. Angst en onzekerheid zijn natuurlijke, gezonde reacties van je lichaam. Deze reacties zorgen ervoor dat je alert kan reageren op gevaarlijke situaties. Door langdurige angst en onzekerheid kun je echter ook nerveus worden, doemdenken, slecht slapen of eindeloos piekeren. Op zulke momenten kunnen angst en onzekerheid je dagelijkse leven sterk beïnvloeden, doordat je bijvoorbeeld activiteiten gaat vermijden die wel belangrijk voor je zijn. Bij een kleinere groep mensen blijven nare herinneringen aan de operatie zich aan hen opdringen. Dit is ook een signaal van angst en/of onzekerheid. Als jij en/of je naaste(n) last hebben van angst-of onzekerheidsklachten, dan is het verstandig om daarvoor hulp te zoeken. Je behandelend arts, huisarts en praktijkondersteuner huisartsenzorg GGZ kunnen je helpen.

Het is mogelijk dat je somber en/of verdrietig bent na je operatie. Dit zijn normale reacties op nare gebeurtenissen. Door langdurige somberheid en verdriet kun je snel bezorgd en/of prikkelbaar worden, snel moe zijn, doemdenken, slecht slapen of eindeloos piekeren. Somberheidsklachten en langdurig verdriet kunnen veel invloed hebben op het leven van jou en je naaste(n). Het is daarom van belang om aandacht te besteden aan het leren omgaan met somberheid en verdriet. Je behandelend arts, huisarts en praktijkondersteuner huisartsenzorg GGZ kunnen je helpen.

Het is mogelijk dat je spanning ervaart na je operatie. Spanning is een natuurlijke, gezonde reactie van je lichaam op stress. Deze reactie zorgt ervoor dat we goed kunnen presteren en alert kunnen reageren op gevaarlijke situaties. Door langdurige spanning en stress kun je echter ook nerveus worden, moeilijk je grenzen aangeven, zaken opkroppen of eindeloos piekeren. Op zulke momenten kunnen spanning en stress je dagelijkse leven sterk beïnvloeden. Als jij en/of je naaste(n) last hebben van stress of spanning, dan is het verstandig om daarvoor hulp te zoeken. Je behandelend arts, huisarts en praktijkondersteuner huisartsenzorg GGZ kunnen je helpen.

Het is mogelijk dat je concentratievermogen minder dan normaal is na je operatie. Dit is normaal na een operatie. Het komt door het gebruik van de hart-longmachine, de narcose en alles wat je in het ziekenhuis hebt meegemaakt. Door het oppakken van je normale leven merk je dat het concentreren vanzelf beter gaat. Gun jezelf de tijd om je concentratievermogen te herstellen.

Het is mogelijk dat je je moe of labiel voelt na de operatie. Zo kun je last hebben van huilbuien of andere emotionele uitbarstingen. Het zijn ontladingen van alle spanningen rondom de operatie. Misschien voel je je er ongemakkelijk bij, maar geef er vooral aan toe. Maakt je niet teveel zorgen. Het hoort erbij en het gaat over.

Familie en Naasten

We adviseren om vóór de opname na te denken of je na de operatie voldoende opvang hebt. Hoewel je na de operatie alweer vrij snel een aantal dingen zelf kunt doen, zijn er ook dagelijkse handelingen die je in het begin nog niet zelf kunt uitvoeren. Denk aan huishoudelijk werk en het tillen van zware boodschappen. Het geeft je meer rust tijdens de ziekenhuisopname als je weet dat dit soort zaken geregeld zijn.

We adviseren de eerste tien dagen overdag (grootste deel) en ’s nachts niet alleen thuis te zijn. Wanneer je ondersteuning nodig hebt kan degene die bij je is voor je zorgen en/of contact opnemen met je behandelend arts of huisarts. Na tien dagen is dit niet meer nodig. De persoon die je opvangt en ondersteunt hoeft niet steeds dezelfde persoon te zijn. Als je na thuiskomst niet terug kunt vallen op familie of naasten, is het belangrijk dit te bespreken met je behandelend arts in het ziekenhuis. Je kunt dan eventueel hulp krijgen van thuiszorg of een revalidatieplek.

We adviseren de eerste zes weken na de operatie met mate bezoek te ontvangen. Het ontvangen van bezoek is leuk, maar vaak ook vermoeiend. Probeer het bezoek daarom goed verspreid te plannen en geef duidelijk aan wanneer je moe bent

Supplementary Material S2. First draft lifestyle recommendations for patients with thoracic aortic surgery in Dutch (full version)

Gedragsverandering

0535

De behandeling bestaat altijd uit het opvolgen van leefstijladviezen. Dit betekent dat u advies krijgt om uw leven aan te passen bijvoorbeeld: stoppen met roken, afvallen, gezond eten, het verminderen van zoutinname, voldoende bewegen, beperkt alcoholgebruik, het voorkomen van stress. Het is belangrijk dat u de adviezen goed opvolgt, om hart-en vaatziekten te voorkomen. Als u dit moeilijk vindt, bespreek dit dan met uw arts.

2312

We raden aan algemene leefregels op te volgen.

Een aantal risicofactoren verhoogt de kans op het krijgen van hart- en vaatklachten. Door gezond te leven vermindert u dit risico. Gezond leven houdt in: zorgen voor voldoende beweging, gezonde voeding, goede stressbalans en niet

 © 2024. The Author(s).

roken. Risicofactoren versterken elkaar: hoe meer risicofactoren, hoe groter de kans op hart- en vaatziekten.

Een gezonde leefstijl helpt. Dat betekent voldoende bewegen, niet roken, matig alcoholgebruik, gezonde voeding en ontspanning. Dat is echter makkelijker gezegd dan gedaan. Veel mensen hebben er moeite mee om hun leefgewoonten te veranderen. Toch is het erg belangrijk dat u zich bewust bent van uw leefstijl en deze waar nodig verandert. Een gezonde leefstijl draagt altijd bij aan een betere gezondheid.

2491
Behalve lichamelijke training is het ook belangrijk dat u goed geïnformeerd bent over uw eigen hartziekte. In het ziekenhuis is daar niet altijd voldoende tijd voor, omdat patiënten tegenwoordig snel naar huis gaan. Bovendien blijkt dat mensen zo kort na een hartinfarct, dotterbehandeling of hartoperatie veel te verwerken hebben. De verstrekte informatie wordt dan vaak onvoldoende onthouden. De vragen komen pas als u uit het ziekenhuis bent ontslagen en u uw leven weer gaat oppakken. Voor deze vragen is ruimte binnen het hartrevalidatieprogramma.

Daarnaast kunt u begeleiding krijgen bij bijvoorbeeld stoppen met roken, afvallen, stresshantering en werkhervatting. Uw hartaandoening kan leiden tot zorgen, onzekerheden of vragen bij uw partner. Tijdens het hartrevalidatieprogramma wordt ook hier aandacht aan besteed.

3748
Ongezonde gedragingen en leefgewoonten zoals roken, ongezond eten, te veel alcohol drinken, te weinig bewegen zijn vaak moeilijk te veranderen. De Leefstijlmodule biedt intensieve begeleiding en ondersteuning.

3912
Tijdens een hartrevalidatieprogramma: leert u uw (lichamelijke) grenzen kennen en verleggen; doet u aan sport en spel; leert u beter te ontspannen; krijgt u begeleiding in de sociale en psychische kant van het leven na een hartinfarct; krijgt u voorlichting over risicofactoren en adviezen over eten en bewegen. U kunt het hartrevalidatieprogramma gebruiken om een plan te maken voor de toekomst en uw leven gezond (er) in te richten.

4078
Het doel van het hartrevalidatieprogramma is om u in een zo goed mogelijke fysieke, sociale en psychische conditie te brengen. Daarnaast begeleiden diverse professionals u in het ontwikkelen en/of behouden van een gezonde leefstijl om het risico op een nieuw hartincident te verkleinen.

Na het intake gesprek met de cardiovasculair verpleegkundige wordt een individueel behandelprogramma opgesteld. De behandeldoelen kunnen zijn: lichamelijk - opbouwen van conditie, grenzen leren kennen; psychisch - omgaan met angst, spanning en depressie; sociaal - werkhervatting, hobby's, dagindeling, relaties; voorlichting over voeding en leefwijze.

4210
Uw leefstijl kan een rol spelen bij het krijgen van een hartaandoening. Belangrijke risicofactoren zijn: roken, ongezond eten, te weinig bewegen en stress. Daarom is werken aan een gezonde leefstijl een belangrijk onderdeel van hartrevalidatie. Hiermee leert u de risicofactoren zo laag mogelijk te houden.

5892
Tijdens het hartrevalidatieprogramma krijgt u informatie over verschillende onderwerpen: stoppen met roken; gezonde voedingsgewoonten; omgaan met stress; (opnieuw) beginnen met werken; sport en vrije tijd; seksualiteit; omgaan met het stellen van grenzen, omgaan met angstige en sombere gevoelens.

7037
Het aanpassen van uw leefstijl is een van de doelen van hartrevalidatie. In het programma leert u uw lichamelijke grenzen kennen en verleggen. Er is aandacht voor sport en spel, maar ook voor de sociale en psychische kant van het leven na een hartklepoperatie. Bijvoorbeeld voor het leren omgaan met uw hartklepaandoening en angsten, seksualiteit, het terugvinden van uw zelfvertrouwen, het hervatten van werk, huishoudelijke taken of hobby's.

Door gezond te leven wordt de kans op hart- en vaatziekten verkleind. Als u wilt omschakelen naar een gezonde leefstijl komt daar veel bij kijken. U moet u écht committeren aan een gezonde levensstijl. Gezondheid is voor 90% een kwestie van discipline om gezonder te leven. Onder gezond leven wordt verstaan: Gezond, maar met name minder eten; Voldoende beweging; Gezond lichaamsgewicht; Niet roken; Bepekt alcohol gebruik; Stress vermijden.

9636
Vaatziekten zijn vaak het gevolg van meerdere gezondheidsproblemen zoals hoge bloedruk, overgewicht en suikerziekte. Uw behandelaars kijken daarom ook altijd naar de oorzaak van de ziekte. In veel gevallen kan een aanpassing van uw leefstijl uw klachten verminderen of de uitkomsten van uw behandeling verbeteren. Adviezen over levenswijze vormen een belangrijk onderdeel van uw behandeling; denk bijvoorbeeld aan voedingsadviezen en hulp bij stoppen met roken.

Volhouden van lichamelijke activiteiten. De meeste mensen hebben positieve ervaringen met bewegen en voelen zich er beter door. Hieronder een aantal tips om het wellicht gemakkelijker te maken om het bewegen vol te houden. Als het moeilijk is om het bewegen vol te houden vanwege tijdgebrek, plan de activiteit dan van tevoren en probeer er een gewoonte van te maken. Maak het onderdeel van uw dagelijkse routine. Probeer een activiteit waaraan u plezier beleeft. Breng variatie aan. Wissel bijvoorbeeld lopen af met fietsen. Stel doelen voor uzelf op (bijv. naar de winkel lopen). Probeer uw motivatie vast te houden: schrijf de redenen op die u heeft om te starten met bewegen en lees deze regelmatig nog eens door. Herken belemmeringen die u ervaart bij

 © 2024. The Author(s).

het volhouden van het bewegen. Probeer oplossingen te vinden voor deze barrières. Vraag iemand anders om met u mee te gaan, bijvoorbeeld een familielid, een vriend of collega. Of kijk op www.beweegmaatje.nl Beloon uzelf. Wees niet te streng voor uzelf.

Lichaamsgewicht

2312
Verder geldt voor iedereen dat overgewicht slecht voor de gezondheid is. Afvallen, al is het een paar kilo, levert dan winst voor de gezondheid op. Mocht u meer informatie over voeding of begeleiding bij een dieet willen, kunt u het beste bij een diëtiste terecht.

Overgewicht is een van de belangrijkste volksgezondheidsproblemen van dit moment. Van de volwassenen heeft ongeveer 50% overgewicht. De hoofdoorzaak van overgewicht is een verstoorde energiebalans: te weinig bewegen ten opzichte van de hoeveelheid eten die genuttigd wordt. Overgewicht kan erfelijk bepaald zijn, maar ook psychologische aspecten en de invloed van de omgeving (zowel lichamelijk als sociaal) op het voedings- en beweeggedrag kunnen invloed hebben. Voor mensen met overgewicht is afvallen gunstig voor de gezondheid. Het cholesterolgehalte in het bloed daalt en ook de bloeddruk wordt lager. Daarmee wordt de kans op hart-en vaatziekten en op slaapapneu kleiner. Voor meer informatie kunt u contact opnemen met de hartrevalidatieverpleegkundige of bij de huisarts een verwijzing vragen voor een diëtiste.

Voor veel mensen is een gewichtstoename een ongewenste bijwerking van het stoppen met roken. Wees hierop voorbereid, maar laat u er niet door ontmoedigen. De gewichtstoename kan beperkt blijven, wanneer u van een goed voedingsplan gebruik maakt. Bespreek dit vooraf met uw arts/verpleegkundige en laat u eventueel verwijzen naar een diëtist.

3912
Uw lichaam heeft na de operatie alle energie nodig om te herstellen. Een dieet om af te vallen, raden we daarom in de periode voor de operatie af.

4078
Na de operatie kan uw lichaam vocht vasthouden. Het lichaamsgewicht kan snel toenemen. Om uw gewicht in de gaten te houden is het belangrijk dat u zich de eerste twee weken dagelijks weegt tot uw controle bij de cardiovasculair verpleegkundige. Doe dit steeds op hetzelfde tijdstip, bijvoorbeeld voor het ontbijt en nadat u naar het toilet bent geweest. Omdat zout vocht vasthoudt, mag u niet teveel zout gebruiken. Als u meer dan twee kilo aankomt in drie dagen, neem dan contact op met uw huisarts.

7037
Als u overgewicht heeft, zijn voedingsmiddelen met relatief weinig calorieën de basis voor uw voeding. U moet dan zo veel mogelijk kiezen voor de ondergrens van de hoeveelheden voedingsmiddelen en de "bij voorkeur" producten. U gebruikt dan een voeding van ca. 1300 kcal. Probeer alleen verantwoorde tussendoortjes te eten, met zo min mogelijk calorieën.

9042
Zorg voor een goed gewicht! Meetinstrumenten hiervoor zijn: De Body Mass Index >25 kg/m^2 (gewicht in kg/lengte in m^2). Een BM' 25 tot 30: licht overgewicht. Een BM' >30: overgewicht.

De middelomtrek. Vrouwen: >88 cm. Mannen: >102 cm. Met afvallen wordt bedoeld lichaamsvet verbranden. Lichaamsvet verbrandt u door minder energie te gebruiken en meer te bewegen. Van alleen water drinken valt u dus niet af. Een paar kilogram gewichtsvermindering geeft al gezondheidswinst!

De praktijk van het afvallen: Eet 3 hoofdmaaltijden; Sla geen maaltijd over; Wees zuinig met vet, suiker en alcohol; Neem tussen de hoofdmaaltijden door een calorie-arme snack om de honger te stillen, spreid de voeding goed over de dag; Motivatie en planning zijn onmisbaar; Zorg voor minimaal 30 minuten lichaamsbeweging per dag.

Voeding

0535
Voeding speelt een grote rol bij uw herstel. Het kan voorkomen dat u na de operatie langere tijd een slechte eetlust hebt, waardoor uw gewicht fors kan afnemen. De diëtiste komt bij u langs om uw voeding met u te bespreken. Ook als uw eetlust stabiel blijft, is het belangrijk dat u gezonde voeding gebruikt (zie folder).

Tijdens de revalidatie krijgt u voorlichting over gezond eten. Eerder voorgeschreven diëten kunt u hervatten. Overleg met de arts indien u voor de operatie een zoutbeprekend dieet volgde. Zoutbeperking is na de operatie vaak niet meer nodig.

2312
In de Schijf van Vijf vindt u alleen producten die gezond voor u zijn. Kies elke dag genoeg uit elk vak en varieer. Eet 2 x dag groenten en fruit en kies voor volkoren brood. Gebruik onverzadigde vetten op de boterham en wissel vlees, kip, vis en vegetarische gerechten regelmatig af. Eet minimaal 1 × week vis en drink voldoende.

Voeding: Eet gezond en niet te veel. Wees matig met vet en voorzichtig met zout. Indien er sprake is van overgewicht is het verstandig om af te vallen. U kunt dit bespreken met uw huisarts of met een diëtiste.

Een dieet is meestal niet nodig. Maar gezond en matig eten is voor iedereen belangrijk. De meeste hartpatiënten krijgen cholesterolverlagende medicijnen voorgeschreven. Daarnaast kunnen mensen profijt hebben van een cholesterolverlagend dieet. Voor patiënten met een verhoogde bloeddruk is het verstandig om een zoutbeperkt dieet te volgen.

3912
We adviseren u natuurlijk wel om gezond te eten.

 © 2024. The Author(s).

4078
Over het algemeen hoeft u geen dieet te volgen. Het is vooral belangrijk dat u gezond eet en voldoende beweegt. Als u bijvoorbeeld overgewicht of een ongezond voedingspatroon heeft, dan is er op de polikliniek Hartrevalidatie een programma om u hierbij te ondersteunen. De cardiovasculair verpleegkundige kan u hierover meer informatie geven. Voor meer informatie verwijzen wij u naar de folder "Eten naar hartenlust" van de Nederlandse Hartstichting en www.voedingscentrum.nl.

4210
Probeer gezond te (blijven) eten. Heeft u overgewicht, blijf hier dan aan werken. Heeft u na de revalidatie nog vragen? Neem dan contact met ons op. Onze diëtiste kan dan met u bespreken wat u verder kunt doen.

5892
Kijk eens kritisch naar uw eetpatroon. Te vet, te veel, te zout? Voldoende vezels, groente en fruit? Voldoende variatie?

5949
Uw lichaam heeft de neiging om vocht vast te houden. Zoutarm eten kan dit tegengaan. We raden u daarom aan om vooral de eerste weken zuinig te zijn met zout.

Voeding is een belangrijk onderdeel van het herstel na de operatie. Het kan voorkomen dat u na de operatie langere tijd een slechte eetlust hebt, waardoor uw gewicht fors afneemt. Samen met een diëtiste bekijkt u hoe uw voedingstoestand verbeterd kan worden. Daarnaast is een vezelrijk dieet belangrijk voor een goede stoelgang. Informatie over een gezonde voeding vindt u in het boekje "Een hart voor goede voeding" dat u na uw operatie krijgt van de voedingsconsulenten.

Het kan voorkomen dat u door de bedrust een slechte eetlust hebt. In dat geval is het raadzaam een diëtist in te schakelen om uw voedingstoestand te verbeteren. Daarnaast is een vezelrijk dieet belangrijk voor een goede stoelgang. Informatie over een gezonde voeding vindt u in het boekje "Een hart voor goede voeding." De voedingsconsulente geeft u graag een exemplaar.

Na de operatie heeft uw lichaam de neiging vocht vast te houden. Omdat zout ook vocht vasthoudt, raden we u aan om vooral de eerste weken zuinig te zijn met het gebruik van zout.

Het is niet altijd nodig om met een cholesterolverlagend dieet te beginnen. Als dat voor u wel nodig is, dan mag u daar pas na de eerste controle bij uw eigen cardioloog mee beginnen. De eerste 3 weken dat u thuis bent, hoeft u dus in ieder geval niet cholesterolarm te eten.

Na de operatie heeft uw lichaam de neiging vocht vast te houden. Zoutarm eten kan dit tegengaan; daarom raden we u aan om met name de eerste weken zuinig te zijn met het gebruik van zout.

7037
Onder gezond eten wordt verstaan dat u bewuste en gezonde keuzes maakt wat betreft de voedingsmiddelen die u eet. Als basis voor gezond eten kunt U de Schijf van Vijf gebruiken die is samengesteld door het Voedingscentrum te Den Haag. In de Schijf van Vijf staan vijf regels centraal. Deze regels staan voor "gezond eten" en kunnen u helpen om richting te geven aan in uw voedingspatroon.

De vijf regels zijn (1) Eet gevarieerd. (2) Eet niet te veel. (3) Gebruik minder verzadigd vet. (4) Eet veel groente en fruit. Eet voldoende brood. (5) Ga veilig en verstandig met het voedsel om.

De vijf vakken in de schijf beelden de eerste regel "Eet gevarieerd" uit. Als u uw voeding kiest uit deze vijf vakken, dan bent u al op de goede weg naar een gezond leven. Het levert de voedingsstoffen op die nodig zijn om het lichaam gezond te houden en het risico op chronische ziekten (waaronder hart-en vaatziekten) te verkleinen.

Een goed voedingspatroon bestaat niet alleen uit variatie maar ook uit bepaalde hoeveelheden. In onderstaand schema staat aangegeven hoeveel u van welke voedingsmiddelen per dag kunt gebruiken. U krijgt dan voldoende voedingstoffen (bouwstoffen, vitamines en mineralen) binnen. De richtlijnen zijn per leeftijdscategorie aangegeven. De precieze hoeveelheid per voedingsmiddel is afhankelijk of u man of vrouw bent. Voor de vrouwen gelden de kleinste hoeveelheden. Naast deze hoeveelheden is er enige ruimte voor extra tussendoortjes per dag voordat u te veel energie (calorieën) binnen krijgt (denk hierbij aan een handje nootjes, een plakje ontbijtkoek of een mueslireep).

Verzadigd vet is het soort vet dat een zeer hoge calorische waarde bevat. Je hoeft hier maar weinig van te eten, om makkelijk dik van te worden. Overgewicht en met name de bijbehorende insuline resistentie (voor loper voor suikerziekte) vergroot het risico op hart- en vaatziekten. Verzadigd vet komt vooral voor in pakjes harde margarine en bak- en braadvet in een wikkel. Veel voedingsmiddelen bevatten verzadigd vet zonder dat dat meteen zichtbaar is. Het vet is als het ware in het product "verborgen." Het gaat dan bijvoorbeeld om volvette kaas (48 +), worst, vet vlees, volle melkproducten, koek, gebak, snacks en zoutjes. Aangezien we hier over het algemeen dezelfde hoeveelheid van eten als van magere producten, kun je zomaar een 2 tot wel 4 maal grotere hoeveelheid calorieën mee binnen krijgen, waardoor je ongewenst in gewicht aankomt.

In onderstaande tabel zijn voedingsmiddelen ingedeeld in drie categorieën namelijk, "bij voorkeur," "middenweg," en "bij uitzondering." "Bij voorkeur" betekent dat in deze productgroep het genoemde product de meest optimale combinatie bevat van zo min mogelijk verzadigd vet en dus ook calorieën en zo veel mogelijk vezels. Deze indeling geeft een gezonde richting aan voor uw eetgedrag. Probeer zo min mogelijk producten genoemd " bij uitzonderingsproducten" te gebruiken. Of compenseer het met een "bij voorkeur product" zodat u een goede balans krijgt tussen "gezonde" en "minder gezonde" keuzes. Probeer kant en klare producten te vermijden. Deze producten bevatten vaak onnodig veel calorieën die het risico op hart-en vaatziekten kunnen vergroten.

8022
Zorg voor goede voeding, dan herstelt u beter. Omdat uw lichaam nu de neiging heeft om vocht vast te houden, is

 © 2024. The Author(s).

zoutarm eten verstandig. Wees vooral de eerste tijd zuinig met zout.

8858
Voeding speelt een belangrijke rol in het voorkomen van hart- en vaatziekten. Gezonde voeding draagt bij aan een goed gewicht, normale bloeddruk en gezonde cholesterolwaarden. Voor een gezond voedingspatroon zijn de volgende zaken van belang:

Gebruik vooral meervoudig onverzadigde vetten.Eet bij voorkeur twee maal per week (vette) vis. Eet dagelijks 200 gram groente en 2 stuks fruit. Beperk het gebruik van zout. Beperk het gebruik van alcohol. Voor meer informatie over een gezond voedingspatroon kunt u terecht bij het Voedingscentrum.

9042
Schijf van vijf: Veel groente en fruit; Vooral volkoren; Minder vlees en meer plantaardig; Genoeg zuivel; Zachte of vloeibare smeer- en bereidingsvetten; Voldoende vocht.

Extra adviezen: Meer peulvruchten; Dagelijks noten; Dagelijks thee; Gefilterde koffie; Minder vruchtensap; Liever geen alcohol

Om te bakken en te braden kunt u het beste vloeibaar bak- en braadvet gebruiken i.p.v. olijfolie. Het klopt dat alle soorten olie en vloeibaar bak- en braad rijk zijn aan onverzadigde vetten, maar vloeibaar bak- en braad bevat ook nog vitamine A en D.

De leeftijd van de kaas zegt niets over het vetgehalte van de kaas. Om het vetgehalte aan te geven worden de termen volvet of het percentage vet gebruikt:48 + , 40 + , 30 + , etc.

Vette vis: (Bijv. zalm, makreel, sprot, bokking, haring.) Het advies is 1 x per week vette vis te eten. Eet niet meer dan 4 porties vette vis per week (maximaal 600 gram) i.v.m. dioxines. Bij onbeperkt gebruik van vette vis is de kans groot dat u teveel vet binnen krijgt.

Eieren, orgaanvlees, paling en garnalen: Bij een te hoog cholesterolgehalte is het vooral van belang matig te zijn met verzadigd vet. Verder heeft het cholesterol in voedingsmiddelen, zoals in eieren, orgaanvlees, paling en garnalen, een klein effect op het cholesterolgehalte. Het advies is maximaal 2-3 eieren per week en circa 100 gram garnalen, lever of niertjes per 2 weken te gebruiken.

Tips voor een beter vetgebruik: Bak in vloeibaar bak- en braadvet of olie; Smeer halvarine of margarine met weinig verzadigd vet op het brood; Kies magere vleessoorten en 20+ of 30+ (smeer)kaas; Eet 1 keer per week (vette) vis; Gebruik ook eens vis als broodbeleg; Neem in plaats van een blokje kaas of plakje worst liever wat nootjes of een toastje met vis

Snacks: Voor een keertje kan een vette snack geen kwaad. Zoals voor alles geldt: uitzonderingen bevestigen de regel. Bedenk wel hoe vaak "voor een keertje" is.

Tips voor minder zoute maaltijden: Gebruik vleeswaren en kaas met minder zout; Voeg zo min mogelijk zout toe aan de maaltijd; Neem minder kant-en-klaar producten; Beperk het gebruik van zoute smaakmakers, zoals: maggi, aromat, bouillonblokjes, sojasaus, sambal en kruidenmixen; Beperk het gebruik van gemarineerd, gekruid of gepaneerd vlees of vis

9909
Wij adviseren u om minstens één keer, maar bij voorkeur twee keer per week vis te eten. In landen waar veel vis wordt gegeten komen hart- en vaatziekten minder voor. Vooral vette vis bevat goede vetzuren. Voorbeelden van vette vis zijn: zalm, haring, makreel, sprot en heilbot.

We adviseren u om per dag twee stuks fruit en 200 gram groente te eten. Eet bij voorkeur volkorenbrood in plaats van wit brood. Mensen die veel groente en fruit eten hebben minder kans op een hartinfarct of een herseninfarct.

Als uw bloeddruk te hoog is, dan kunt u uw gezondheidsrisico verminderen door af te vallen, meer te bewegen, eventueel minder alcohol te gebruiken en meer groente en fruit te eten. Maar ook minder zout gebruiken helpt om uw bloeddruk te verlagen. Denk ook aan hartige snacks en kant-en klare soepen en sauzen: die bevatten veel toegevoegd zout. Wilt u minder zout gebruiken? Probeer dan een deel van het zout bij het bereiden van de maaltijd weg te laten of te vervangen door verse kruiden.

Alcohol en drugsgebruik

2312
U mag één à twee glazen alcohol per dag drinken, maar bij voorkeur niet elke dag. Alcohol wordt, net als veel medicijnen, door de lever afgebroken. Dit kan maar in beperkte mate. Daarnaast verhoogt alcohol de bloeddruk en bevat het veel calorieën.

3912
Stop tenminste 2 weken vóór de operatie met het gebruik van alcohol. Zo voorkomt u dat u klachten kunt krijgen door het stoppen. Deze kunt u nog krijgen 10 tot 12 dagen na het stoppen. Stoppen met het gebruik van alcohol doet u voor uzelf, om de kans op schade aan uw gezondheid zo klein mogelijk te maken! Heeft u hulp nodig bij het bij het stoppen met het gebruik van alcohol? Wij adviseren u om te informeren bij uw huisarts ofuw cardioloog.

5892
Wees matig met alcohol. Alcohol kan de werking van medicijnen versterken.

5949
Matig alcoholgebruik is na een aortadissectie type A en/of type B geen probleem. Maar gebruik liever niet meer dan twee glazen per dag. Gebruikt u Sintromitis of Marcoumar® én gebruikt u meer dan twee glazen alcohol per dag? Geef dit dan door aan de trombosedienst. Alcohol kan de bloedverdunnende werking van Sintromitis namelijk versterken

7037
Beperk uw alcohol gebruik naar maximaal 1-2 Eh alcohol per dag (voor vrouwen maximaal 1 Eh per dag). Niet alleen bevat

alcohol erg veel calorieën, die uw gewicht weer nadelig kunnen beïnvloeden, alcohol heeft ook zijn weerslag op uw cognitieve vermogens en wekt verslaving in de hand.

7041
U mag 1-2 glazen alcohol per dag gebruiken. Als u bloedverdunners gebruikt, geef dan uw alcoholgebruik door aan de trombosedienst.

8022
U mag matig alcohol gebruiken na de aortabehandeling. Het advies is om maximaal 1 glas alcoholhoudende drank per dag te drinken.

9909
Gebruik alcohol met mate. Dit betekent voor vrouwen niet meer dan twee en voor mannen niet meer dan drie glazen per dag. Gebruikt u alcohol met mate dan hebt u een kleinere kans op (nieuwe) hart- en vaatziekten dan mensen die nooit alcohol gebruiken. Verder is het beter om een alcoholhoudende drank te nemen bij het eten dan tussendoor.

Stoppen met roken

2312
Stop met roken. Indien u weer begint met roken heeft u min of meer dezelfde levensverwachting als wanneer u niet geopereerd zou zijn. Als u moeite heeft met stoppen en u gaat revalideren in X kunt u daar een cursus "Stoppen met roken" volgen.

Roken is slecht voor uw hart en bloedvaten. Wij raden u dan ook af om te roken.

Omdat roken een van de grootste risicofactoren is voor het krijgen van hart- en vaatziekten, maar ook voor een aantal andere ziekten, is het verstandig om te stoppen met roken. Voor meer informatie over advies en begeleiding kunt u contact opnemen met de hartrevalidatieverpleegkundige, of met de praktijkondersteuner van uw huisarts. www.rookvrijookjij.nl

We hoeven u vast niet te vertellen dat roken slecht is voor uw gezondheid. De nicotine in tabak zorgt ervoor dat bloedvaten smaller worden en dat ze beschadigd raken. De bloedvaten worden uiteindelijk steeds nauwer, wat kan leiden tot een hartinfarct. Daarnaast zorgt nicotine ervoor dat het hart sneller gaat kloppen. Ook krijgt het hart minder zuurstof. Meer informatie vindt u op onze aparte pagina:

En roken heeft nóg meer nadelige effecten op uw gezondheid dan u denkt! Naast het verhoogde risico op hart- en vaatziekten, longziekten en kanker, kan roken onder andere ook leiden tot meer hoesten, slechtere conditie, koude handen en voeten en huidverslechtering. Denk ook eens aan alle andere gevolgen van roken, zoals de hoge kosten van een pakje sigaretten en de kosten voor het vaker moeten verven van uw huis. Stoppen met roken levert al na enkele weken gezondheidsvoordelen op, zoals meer lucht, betere geur en smaak, gezondere huidskleur, minder hoesten, warmere handen en voeten en zich fitter voelen. Stoppen met roken levert van alle genoemde leefstijlfactoren de grootste vermindering van het risico.

Het is voor veel mensen niet eenvoudig om te stoppen met roken. Steun uit de omgeving kan helpen. Dat kan steun zijn van familieleden of vrienden, maar ook steun van lotgenoten die u tegenkomt op een cursus Stoppen met roken." Bespreek met uw arts/verpleegkundige wat voor u een goede ondersteuning zou kunnen zijn.

3912
Patiënten die roken hebben meer kans op longproblemen en wondinfecties na een operatie. Rookt u? Dan adviseren wij u: Stop met roken tenminste 8 weken vóór de operatie. Rook na de operatie helemaal niet meer.

Stoppen met roken doet u voor uzelf, om de kans op schade aan uw gezondheid zo klein mogelijk te maken! Maar stoppen met roken is meestal makkelijker gezegd dan gedaan. Extra hulp kan het stoppen makkelijker maken.

5892
Patiënten die roken hebben meer kans op longproblemen en wondinfecties na een operatie.

7037
Door roken raakt de binnenwand van de slagaders beschadigd. Als u nog rookt, dan is dit dé gelegenheid om ermee op te houden. Sommige hartcentra eisen zelfs dat u zes weken vóór de operatie niet rookt, om zo de risico's na de operatie te verkleinen.

Om de kans op hart- en vaatziekten te verminderen is het belangrijk om te stoppen met roken. Als u stopt met roken heeft dit een positieve invloed op uw gezondheid. Het schadelijke effect van de nicotine op de bloedvaten en op de werking van het hart verdwijnt na ongeveer 5 jaar als u stopt met roken. Veel huisartsen hebben een praktijkbegeleider die u kan helpen met het stoppen. Voor meer informatie hierover kunt u met uw eigen huisarts contact opnemen.

8022
Dat roken slecht is voor hart- en bloedvaten weet u vast wel. Rondom een operatie verhoogt roken de kans op luchtweg- en wondinfecties. Voor alle patiënten geldt dan ook een strikt rookverbod. Heeft u hulp nodig bij stoppen met roken, dan kunt u contact opnemen met uw huisarts.

8858
Roken vergroot de kans op hartklachten. De kans dat u opnieuw hartklachten krijgt, is veel kleiner als u stopt met roken. Als u advies en begeleiding nodig hebt bij het stoppen, dan kunt u terecht bij de polikliniek Stoppen-met-roken.

Door roken komen er stoffen in het bloed die schadelijk zijn voor de wanden van de bloedvaten. Deze stoffen beschadigen de vaatwand. Dit kan het begin zijn van het proces van slagaderverkalking (atherosclerose). Het bloed kan hierdoor minder goed doorstromen. Uiteindelijk kan dit leiden tot hart- en vaatziekten. Stoppen met roken is de meest

 © 2024. The Author(s).

effectieve maatregel om het proces van slagaderverkalking af te remmen. Dit geldt ook op oudere leeftijd.

Voor informatie over en hulp bij het stoppen met roken kunt u bijvoorbeeld terecht op de website thuisarts.-nl/stoppen-met-roken of bij de Hartstichting/stoppen-met-roken. Ook kunt u via uw huisarts of behandelend specialist een afspraak maken op de polikliniek Stoppen-met-roken

9909

Stoppen met roken is vaak niet eenvoudig, maar levert wel een enorme vermindering op van uw risico op hart- en vaatziekten. Zoek steun in uw omgeving of praat hierover met uw arts.

Wondgenezing

0535

Zonnebaden. Zodra de wond is genezen, kunt u de huid weer blootstellen aan de zon of onder de zonnebank gaan. Smeer het litteken wel in met een zonnebrandmiddel met hoge beschermingsfactor of een sunblock. Littekenweefsel bevat namelijk weinig of geen pigment en kan snel verbranden.

Sauna. In de eerste weken na de operatie kunt u beter niet naar de sauna gaan. U kunt de sauna weer bezoeken na overleg met uw cardioloog.

2312

Indien u een openhartoperatie hebt gehad is het borstbeen tijdens de operatie in de lengte doormidden gezaagd. Aan het einde van de operatie zijn beide helften weer stevig aan elkaar bevestigd met roestvrijstalen draden. In principe worden deze staaldraden niet verwijderd. De volledige genezing duurt ongeveer drie maanden.

De eerste 6 weken na de operatie mag u: Geen zware dingen tillen; Niet de hond uit laten; Niet te fietsen (hometrainer mag wel); Niet te zwemmen; Niet auto te rijden ; Geen zware huishoudelijke werkzaamheden verrichten

Na zes weken is de genezing van het borstbeen zo ver gevorderd dat de meeste dagelijkse handelingen weer verricht kunnen worden.

Na de operatie kan het gevoel van de borstkas anders zijn dan voor de operatie. Er kan sprake zijn van een doof gevoel of juist een scherpe pijn. Dit komt doordat kleine huidzenuwen enkele maanden nodig hebben om te herstellen.

De operatiewonden zijn over het algemeen dicht als u naar huis gaat. U kunt dan elke dag douchen. Gebruik de eerste dagen geen zeep bij de wonden en dep de wond en het gebied rond de wond droog. Om de voorkomen dat de wond week wordt, raden wij het af om de eerste weken na de operatie een bad te nemen, te zwemmen of de sauna te bezoeken.

Indien zich onder aan de borstwond nog hechtingen bevinden bij ontslag mogen deze 10 tot 14 dagen na de operatie verwijderd worden door de huisarts.

Neem bij tekenen van een wondinfectie (roodheid, warmte, pus uit de wond en/of koorts) direct contact op met de cardio-thoracale chirurgie in het GECODEERD. De telefoonnummers vindt u onderaan dit blad.

Steunkousen. Voor het maken van de omleidingen wordt vaak gebruik gemaakt van een ader uit het been. Het duurt even voordat de functie van deze ader wordt overgenomen door de resterende aders. Dit kan leiden tot vochtophopingen in het geopereerde been. Om dit te voorkomen/behandelen is het belangrijk dat u de steunkous aantrekt voordat u uit bed stapt. Het is de bedoeling dat u deze kous zes weken draagt. De kousen krijgt u van het ziekenhuis en kunt u thuis gewoon wassen.

Indien u een openhartoperatie hebt gehad is het borstbeen tijdens de operatie in de lengte doormidden gezaagd. Aan het einde van de operatie zijn beide helften weer stevig aan elkaar bevestigd met roestvrijstalen draden. In principe worden deze staaldraden niet verwijderd. De volledige genezing duurt ongeveer drie maanden.

Na zes weken is de genezing van het borstbeen zo ver gevorderd dat de meeste dagelijkse handelingen weer verricht kunnen worden. Na de operatie kan het gevoel van de borstkas anders zijn dan voor de operatie. Er kan sprake zijn van een doof gevoel of juist een scherpe pijn. Dit komt doordat kleine huidzenuwtakjes zijn doorgesneden. Dit zal enkele maanden nodig hebben om te herstellen. De operatiewonden zijn over het algemeen dicht als u naar huis gaat. U kunt gewoon elke dag douchen. Gebruik de eerste dagen geen zeep bij de wonden en droog de wond en het gebied rond de wond deppend af. Om te voorkomen dat de wond week wordt, raden wij het af om de eerste weken na de operatie een bad te nemen.

Als zich onder aan de borstwond nog hechtingen bevinden bij ontslag, mogen deze 10 tot 14 dagen na de operatie verwijderd worden door de huisarts.

Bij tekenen van wondinfectie (roodheid, warmte, pus uit de wond en/of koorts) moet u altijd contact op te nemen met het GECODEERD en een spoedafspraak op de wondpoli CTC te laten maken.

De operatiewonden zijn over het algemeen dicht als u naar huis gaat. U kunt dan elke dag douchen. Gebruik de eerste dagen geen zeep bij de wonden en dep de wond en het gebied rond de wond droog. Om te voorkomen dat de wond week wordt, raden wij het af om de eerste weken na de operatie een bad te nemen, te zwemmen of de sauna te bezoeken.

Indien zich onder aan de borstwond nog hechtingen bevinden bij ontslag, mogen deze 10 tot 14 dagen na de operatie verwijderd worden door de huisarts. Neem bij tekenen van een wondinfectie (roodheid, warmte, pus uit de wond en/of koorts) direct contact op met de Polikliniek Hart + Vaat Centrum of buiten openingstijden met verpleegafdeling D4. De telefoonnummers vindt u onderaan dit blad.

4078

Na uw ontslag uit het ziekenhuis is het verstandig om de eerste zeven dagen geen drukke plaatsen te bezoeken, zoals winkels, vanwege infectiegevaar en kwetsbaarheid van de wond.

Het borstbeen is open gezaagd en met draden gehecht. Het herstel van het borstbeen gaat langzaam en duurt gemiddeld zes weken. Na die zes weken wordt de

botstructuur nog sterker doordat het borstbeen weer belast wordt. De helften van het borstbeen kunnen ten opzichte van elkaar niet verschuiven. Til niet meer dan vijf kilo en doe dit met twee armen tegelijk. Rug-, buik- of zijligging is toegestaan, zolang het niet teveel pijn doet. Probeer wel steeds symmetrische bewegingen met beide armen te maken wanneer u tilt, op de zij draait of uit bed komt. Het is mogelijk dat de borstwond nog wat "trekt." Probeer zoveel mogelijk rechtop en ontspannen te lopen en normaal te bewegen.

Het volledige herstel van het borstbeen duurt ongeveer drie maanden. Na zes weken is het borstbeen zover hersteld dat u de meeste dagelijkse dingen weer kunt doen. Hoesten, niezen en persen kunnen de eerste weken gevoelig zijn.

Als u naar huis gaat, is de borstwond meestal dicht. In dat geval kunt u gewoon douchen. U kunt beter geen bad nemen, omdat de wondranden nog week kunnen worden en de wond hierdoor open kan gaan. Gebruik geen poeder of zalf op de wond. Dit maakt de kans op een infectie groter.

De borstwond kan aan de bovenkant wat dik zijn. Dit trekt de komende weken (grotendeels) weg. Als een hechting zichtbaar wordt, laat deze dan zitten. De hechtingen lossen vanzelf op in ongeveer zes weken.

Wij raden vrouwen met zware borsten aan om dag en nacht een beha zonder beugels te dragen. Daardoor staat er weinig spanning op het litteken en is de wond minder pijnlijk. Ook wordt dan het litteken minder breed en dus mooier.

Als u een kleine cupmaat heeft, hoeft u 's nachts geen beha te dragen.

4210

Om ervoor te zorgen dat uw wond goed geneest, moet u zich aan de volgende leefregels houden: U mag 6 weken niet in bad, zwemmen of in de sauna; U mag wel douchen. Zeep mag ook langs de wond stromen; U mag, zolang de wond niet volledig genezen is, geen zalf op de wond smeren. De wond kan dan namelijk gaan ontsteken.

Wanneer u weer thuis bent na de operatie mag u veel, maar sommige dingen niet. De eerste 6 weken zijn nodig om het borstbeen te laten genezen en daarom mag u deze niet te zwaar belasten.

Om het borstbeen rust te geven is het belangrijk dat u goed de kracht in uw beide armen gelijk gebruikt en niet boven schouderhoogte werkt. Probeer met 2 armen tegelijk te werken, dit voorkomt ongelijke krachten op uw borstbeen. De eerste 6 weken mag u niet meer dan ongeveer 2 kilo tillen.

Na 6 weken is het borstbeen voldoende hersteld en mag u uw armen weer volledig gebruiken. Wanneer dit teveel pijnklachten geeft, wacht dan tot een week later en probeer het voorzichtig nogmaals.

Tot 12 weken na de operatie is "normaal" dat het borstbeen nog gevoelig is. Wanneer deze klachten niet overgaan of klachten toenemen, neemt u contact op met uw huisarts.

Heeft u twijfels over de genezing van uw borstbeen? Of heeft u het gevoel dat deze beweegt of knarst? Neem dan contact met ons op.

5949

Als u naar huis gaat is de wond dicht, maar nog wel kwetsbaar. U hoeft de wond thuis niet te verzorgen. Er hoeft ook geen verband meer op. Doe ook geen zalf of poeder op de wond: dit verhoogt de kans op een infectie (ontsteking). Bij een infectie is de wond rood, opgezet en pijnlijk. Soms ook komt er vocht uit de wond en/of hebt u koorts. Vermoedt u dat de wond ontstoken is? Neem dan altijd contact op met de verpleegkundig specialist Hart-Long Chirurgie. Is de wond bij uw ontslag nog niet dicht? Dan krijgt u van de verpleegkundige instructies mee voor de verzorging thuis.

Douchen en in bad. U mag gewoon douchen. Dep de (eventuele) wond na het douchen met een zachte handdoek goed droog. U kunt met een operatiewond de eerste 6 weken beter geen bad nemen. Dit maakt de wond week. Hierdoor geneest de wond minder goed.

Omdat de nieuwe huid snel verbrandt, mag u een jaar lang niet met de wond in de zon liggen: dek de wond af met een pleister. U mag de eerste 6 weken nog niet zwemmen: de wond wordt dan te week. Als u naar huis gaat, is de wond dicht, maar deze is nog wel kwetsbaar. Houd hier rekening mee. Volg daarom de volgende instructies goed op: U kunt gewoon douchen, maar neem de eerste 6 weken nog geen bad. Dit om te voorkomen dat het litteken week wordt. Gebruik geen zeep, poeder of zalf op de wond omdat dit de kans op infectie verhoogt. Is er een hechting zichtbaar? Trek er dan niet aan. De hechtingen lossen vanzelf op in ongeveer 6 weken. Lekt er vocht langs de hechtingen? Neem dan contact met ons op. Vrouwen met een wat grotere borstomvang raden we aan om dag en nacht een BH te dragen (zonder beugels). Dit verlaagt de spanning op het litteken waardoor deze minder pijn zal doen. Ook wordt dan het litteken minder breed en dus mooier. De borstwond kan aan de bovenzijde wat dik zijn. Dit trekt binnen een paar weken vanzelf weg. Vrouwen met een kleinere borstomvang mogen 's nachts de BH afdoen.

Bij sommige operaties (CABG, aorta chirurgie) wordt uw borstkas bij het borstbeen geopend. Aan het einde van de operatie zijn de beide helften van het borstbeen dan weer stevig aan elkaar bevestigd met draden van chirurgisch staal. De beide helften van het borstbeen kunnen daardoor niet ten opzichte van elkaar verschuiven. Is uw borstkas bij uw borstbeen geopend? Houd dan rekening met het volgende: Vanaf 4 dagen na de operatie kunt u zonder probleem op uw zij of buik gaan liggen. Maak steeds symmetrische bewegingen met beide armen wanneer u tilt, op uw zij draait of uit bed komt. Dus houd niet een arm hoog en de andere laag als u de beweging uitvoert, maar maak dezelfde beweging met beide armen. Hoesten, niezen en persen blijven de eerste weken nog gevoelig. Hoort of voelt u bij het hoesten, niezen of uitademen steeds een klik, neem dan contact op met uw behandelend arts. Het duurt ongeveer 3 maanden voordat het borstbeen weer helemaal genezen is. Na 6 weken is de genezing al wel zo ver dat u de meeste dagelijkse dingen weer kunt doen

Draag Steunkousen. Het kan zijn dat er tijdens de operatie een of meer aders uit uw been of beide benen gehaald zijn.

 © 2024. The Author(s).

Daardoor is het terugstromen van het bloed uit het been naar het lichaam tijdelijk verstoord. Als gevolg daarvan kan zich vocht gaan ophopen in uw been of benen. Als dat gebeurt, ziet u dat uw been dikker wordt. U kunt dit voorkomen door de eerste 6 weken na de operatie steunkousen te dragen. Deze draagt u alleen overdag.

Trek de steunkousen niet zelf aan We raden u aan om de steunkousen niet zelf aan of uit te trekken. Dit geeft namelijk teveel druk op uw borstbeen. Hierdoor herstelt het borstbeen minder snel of kan het zelfs losraken (als uw borstbeen tijdens de operatie is geopend). Laat dit daarom iemand anders doen, bijvoorbeeld uw partner of een familielid. Is er niemand in uw omgeving die dit kan doen? Geef dit dan tijdig aan bij de verpleegkundige. Zij vraagt dan thuiszorg voor u aan. Er komt dan iedere dag iemand bij u langs om uw steunkousen aan en uit te trekken. Na verloop van tijd nemen andere bloedvaten vanzelf de functie van het verwijderde bloedvat over en verdwijnen de klachten.

Het afwachten van uw dokters advies is belangrijk, omdat uw operatie wel wat consequenties heeft. Zo mag u bijvoorbeeld de eerste 6 weken: Niet zonnen: uw operatiewond is erg teer. Uw nieuwe huid verbrandt door de zon erg snel! U mag ook niet onder de zonnebank! Niet zwemmen: uw wond (o.a. borstbeen) moet nog genezen.

Houd er rekening mee dat u bij thuiskomst niet meteen alles zomaar weer kunt doen. Eerst moet uw borstbeen nog genezen

Douchen en baden. Douchen is toegestaan. Dep na afloop de wond met een zachte handdoek goed droog. De eerste 6 weken kunt u beter geen bad nemen. Dit maakt de wond week en kan de genezing belemmeren.

7037

Het duurt zes weken voordat de borstbeenhelften weer stevig aan elkaar vastgegroeid zijn. Daarom mag u gedurende de eerste zes weken na de operatie nog niet alles doen. Uw cardioloog zal u meestal adviseren om niet zelf auto te rijden; u mag wel meerijden. U mag niet fietsen op straat (thuis fietsen op een hometrainer mag wel) en u mag geen zware dingen tillen. Voor die eerste weken is de beste manier van revalideren: wandelen. Meestal gaat het herstel in de eerste twee weken vrij snel. Maar dit zet niet altijd meteen door. Het is niet gek dat u na een hartklepoperatie nog maandenlang last heeft van snel optredende moeheid. Het hele lichaam moet nog wennen en zich aanpassen aan de nieuwe toestand. Praat hier ook over met de huisarts en cardioloog en tijdens de revalidatie.

Uw operatiewond is meestal dicht als u naar huis gaat. Als de wondranden nog niet gesloten zijn of als er wat vocht uitkomt, legt de verpleegkundige u uit hoe u de wond moet verzorgen. U krijgt een recept voor de benodigde wondzorgmaterialen mee naar huis. Zo nodig wordt er thuiszorg ingeschakeld voor de wondverzorging. Gebruik geen poeder en zalf op de wond; Soms kan een doorzichtig draadje van een hechting zichtbaar zijn. Trek er dan niet aan. De hechting lost vanzelf op; De hechtingen van de drainwondjes zijn veelal groen van kleur, deze mogen 10 dagen na verwijdering van de drain verwijderd worden door de huisarts of de huisartsassistenten; Felle zon op de wond kan verkleuring van het litteken geven. Daarom is het beter de wond te beschermen tegen de zon; Zijn er problemen met de wond? Dan adviseren wij u contact op te nemen de verpleegafdeling. Afhankelijk van dit gesprek kunnen wij u eventueel vragen om langs te komen; We adviseren vrouwen om een beugelloze BH te dragen. Dit geeft extra steun aan de wond. Vooral bij de knie en de enkel kan het litteken van de wond de eerste weken licht steken. Dit gaat vanzelf over. Doet de wond continu pijn, wordt deze rood en dik en produceert de wond vocht? Dan raden wij u aan om contact op te nemen met de verpleegafdeling.

7096

Bij de meeste hartoperaties is het borstbeen tijdens de operatie in de lengte opengemaakt. Aan het einde van de operatie worden de beide helften van het borstbeen weer stevig aan elkaar bevestigd met roestvrijstalen draden. In principe worden deze hechtingen niet verwijderd.

Het duurt tenminste zes weken voordat het bot volledig aan elkaar vast gegroeid is. Om het borstbeen te ontzien, is het verstandig de eerste zes weken na de operatie de volgende dingen achterwege te laten: zware dingen tillen; een grote hond uitlaten; zware huishoudelijke werkzaamheden, zoals stofzuigen en ramen lappen. Hoesten, niezen, lachen, hikken en persen is de eerste weken soms gevoelig.

Meestal wordt een slagader uit de borstwand gebruikt als omleiding voor een vernauwde kransslagader. De plek waar deze borstslagader is verwijderd, kan doof aanvoelen of overgevoelig zijn. De pijn is duidelijk anders dan de pijn op de borst die u vóór de operatie voelde en is van tijdelijke aard.

Een andere klacht die vaak voorkomt, is dat u uw hart nadrukkelijker in de borstkas voelt bonzen. Vooral in rust ervaart u dit dan sterk. Dit komt doordat het weefsel van en rondom het hart is geprikkeld door de operatie. Ook dit verschijnsel is doorgaans onschuldig en tijdelijk van aard.

Zon en zonnebank. Het is, met het oog op de wondgenezing, niet verstandig om de eerste maanden na de operatie de littekens bloot te stellen aan felle zon of de zonnebank. Een gezichtsbruiner of het gebruik van sun block crèmes op de littekens is wel toegestaan. PI-004089.versie 6.0 / 09-07-2018

8022

Wassen. U mag gewoon douchen, maar liever nog niet baden.

9042

Week 8: Houdt u van zwemmen? Dan kunt u dit na 8 weken weer oppakken, mits de wond droog is. Anders is er kans op infecties.

Sedentaire leefstijl en lichamelijke activiteit – Voor de operatie

0535

Bewegingsadviezen. Blijf vóór de hartoperatie zoveel mogelijk bewegen. Als u zich goed genoeg voelt, probeer dan door te gaan met bijvoorbeeld wandelen, fietsen en traplopen.

Twijfelt u of u een bepaalde activiteit mag doen? Neem dan contact op met uw arts of fysiotherapeut.

2491
Het is belangrijk om in de aanloop naar de operatie te blijven bewegen binnen uw mogelijkheden. Het is verstandig om te kiezen voor een activiteit die niet te belastend is en die u langere tijd vol kunt houden. Denk hierbij aan wandelen of fietsen.

9636
Voor uw opname. Over het algemeen geldt: hoe beter u een ziekenhuis conditioneel in gaat, hoe beter u er uit komt. Overleg met uw cardioloog hoe u uw conditie in de periode voorafgaand aan de operatie kunt opbouwen.

Sedentaire Leefstijl en Lichamelijke Activiteit – Verzorgen

0535
Na de operatie kunt u zichzelf wel weer verzorgen.

Dagelijkse Verzorging. Na enkele dagen zult u weer steeds meer zelf gaan doen, zoals uit bed komen, wassen en aankleden. De verpleegkundigen zullen u hierbij begeleiden en waar nodig helpen.

Lichamelijke verzorging. Neem rustig de tijd voor uw persoonlijke verzorging, zoals wassen, douchen en scheren.

5949
Na thuiskomst kunt u wel al rustig aan in beweging komen. U mag: licht huishoudelijk werk doen, zoals afwassen, koken of stoffen; (als het weer het toelaat) wandelen en een boodschapje doen; douchen.

7041
Douchen: U mag gewoon douchen. Als u geopereerd bent kunt u na het douchen de wond voorzichtig droogdeppen. U kunt de eerste zes weken beter geen bad nemen, dat maakt de wondranden week.

Sedentaire Leefstijl en Lichamelijke Activiteit – Huishouden

0535
U mag nog geen huishoudelijk zwaar werk doen

Week 1: Een kleine afwas of het maken van een kleine maaltijd is prima.
Week 2: U kunt lichte huishoudelijke taken zoals afstoffen en afwassen uitvoeren.
Week 3-4: U kunt huishoudelijke en sociale activiteiten rustig verder uitbreiden.

2312
geen zware huishoudelijke werkzaamheden verrichten

4078
Huishoudelijk werk. Het doen van huishoudelijke werk is geen probleem. Houdt u er rekening mee dat u bij thuiskomst nog niet alles kunt. Licht huishoudelijk werk, zoals afwassen, koken en stoffen, is geen probleem. Zwaarder huishoudelijk werk, zoals stofzuigen, dweilen, kinderen tillen, hond uitlaten en de grotere boodschappen, moet u de eerste zes weken aan anderen overlaten.

Niet stofzuigen of de hond uitlaten. Dit omdat dit asynchrone activiteiten zijn. Bij deze activiteiten is de druk op beide helften van het borstbeen niet gelijk, waardoor er een kans bestaat dat het borstbeen los laat.

5892
Huishoudelijke werkzaamheden. De eerste weken na uw ontslag uit het ziekenhuis moet u inspannende werkzaamheden (zoals stofzuigen, ramen zemen, toilet en douche schoonmaken) vermijden. Probeer het werk zoveel mogelijk te spreiden over de dag en de week. Loop bijvoorbeeld liever twee keer met lichte boodschappentas dan één keer met een te zware.

Tuinieren. De eerste weken na uw ontslag uit het ziekenhuis mag u niet grasmaaien of de tuin omspitten. Doe niet te lang achter elkaar te zwaar werk.

5949
Huishoudelijk werk. Houd er rekening mee dat u bij thuiskomst nog niet alles kunt. De eerste 6 weken mag u alleen licht huishoudelijk werk doen. Denkt u aan afwassen, koken, stoffen, een boodschapje doen en wandelen. Na 6 weken mag u zwaarder huishoudelijk werk doen, zoals stofzuigen, dweilen, kinderen tillen, de hond uitlaten en grotere boodschappen doen.

Na thuiskomst kunt u wel al rustig aan in beweging komen. U mag: licht huishoudelijk werk doen, zoals afwassen, koken of stoffen; (als het weer het toelaat) wandelen en een boodschapje doen; douchen.

Eerste zes weken NIET: zwaarder huishoudelijk werk, zoals stofzuigen, dweilen of zware boodschappen dragen.

Dagelijkse activiteiten. Houd er rekening mee dat u bij thuiskomst nog niet alles kunt. De eerste 6 weken mag u alleen licht huishoudelijk werk doen, zoals afwassen, koken, stoffen, een boodschapje doen en wandelen. Na 6 weken mag u zwaarder huishoudelijk werk doen, zoals stofzuigen, dweilen, kinderen tillen, de hond uitlaten en de grotere boodschappen doen.

7041
Dagelijkse activiteiten: U kunt als u thuis bent nog niet direct alles doen. De eerste zes weken mag u alleen licht huishoudelijk werk doen. Na zes weken mag u weer zwaar huishoudelijk werk uitvoeren.

8022
Activiteiten. Direct na thuiskomst kunt u nog niet een heel huishouden aan. De eerste zes weken mag u alleen lichte activiteiten verrichten. Zoals wandelen, koken, afwassen, stof afnemen en een kleine hoeveelheid boodschappen doen. Na zes weken kunt u dit uitbreiden met bijvoorbeeld de hond uitlaten, kinderen tillen, stofzuigen, dweilen en grotere hoeveelheden boodschappen halen.

 © 2024. The Author(s).

8935
Huishoudelijk: Op geleide van de klachten, denk aan tillen tot 5 kilogram en de eerste 6 weken zo veel mogelijk symmetrisch bewegen.

9042
Week 1-2: U kunt rustig beginnen met wat lichte bezigheden in huis, bijvoorbeeld licht huishoudelijk werk zoals afwassen en stof afnemen. Verdeel uw taken over de dag, niet alles hoeft in één keer af

9627
Huishoudelijk werk. U mag de eerste 6 weken alleen licht huishoudelijk werk doen zoals koken, afwassen, stoffen of boodschappen doen, maar u mag niet zwaar tillen.

Sedentaire Leefstijl en Lichamelijke Activiteit - Activiteiten

0535
Houding. Het is mogelijk dat de operatiewond nog wat trekt. Probeer zoveel mogelijk rechtop en ontspannen te lopen en normaal te bewegen.

Beweging. Lichaamsbeweging zal u goed doen. In het ziekenhuis heeft u reeds een start gemaakt met het leveren van inspanning bij uw lichamelijke verzorging, bij wandelen en bij traplopen. Thuis gaat u hiermee verder. In het algemeen mag u eigenlijk alles, maar wel met mate.

Inspanning. Voor inspanning geldt dat u het beste kalm kunt beginnen. Voer alles in een rustig tempo uit. Luister daarbij naar uw lichaam; wat u doet, moet goed aanvoelen. Als u moe bent, neem dan een pauze. U hoeft niets te bewijzen.

Wandelen. Wandelen is een gezonde vorm van bewegen. Begin rustig en breidt de wandeling iedere dag een stukje uit. Denk er wel aan dat u ook weer terug moet. Alleen gaan wandelen kan zonder bezwaar.

Traplopen. Pas uw tempo bij het traplopen aan. Neem eventueel tussendoor een pauze.

Tillen. Om wondgenezing te bevorderen, moet u geen zware lasten tillen in de eerste zes weken na de operatie. Gebruik altijd twee handen bij het tillen.

Hobby's en sport. Het hervatten van sport en andere hobby's kunt u na drie weken bespreken tijdens de controleafspraak bij uw cardioloog. De cardioloog zal aangeven of u nog moet wachten tot na de revalidatieperiode.

Om tijdens de operatie bij het hart te kunnen komen is het borstbeen doorgenomen en na de operatie gehecht met staaldraden. Het borstbeen heeft 6 tot 8 weken nodig om volledig te herstellen. Voor een goed herstel is het belangrijk dat u zich aan een aantal leefregels houdt:

Niet duwen of trekken met uw armen; Voorkom ongelijke krachten op het borstbeen; Beperkt tillen (tot 2 kilogram)

Deze adviezen zijn slechts richtlijnen. Iedere patiënt heeft een eigen hersteltempo en –niveau. Gebruik uw gezonde verstand en gun uzelf de beste kans op een vlot herstel. In de eerste fase van het herstel kunt u zich vermoeider voelen dan normaal, maar gaat voorbij als u meer conditie en vertrouwen krijgt. Het is goed om te bewegen en dit uit te breiden, maar let erop dat u niet gaat overbelasten.

Verschijnselen van overbelasting zijn: Pijn of druk op de borst. Kortademigheid of abnormale vermoeidheid die niet in verhouding staat tot de geleverde inspanning. Toename van vocht in het gehele lichaam, armen of benen. Onregelmatige hartslag of hartkloppingen. Overmatig zweten, misselijkheid of duizeligheid.

Als u voor de eerste keer thuis gaat lopen, probeer dan een vergelijkbare afstand en tempo aan te houden als tijdens uw wandeling(en) in het ziekenhuis. U legt een goede afstand af als u enige kortademigheid ervaart bij een prettig tempo. Houd er rekening mee dat u ook nog terug moet lopen. Probeer dagelijks 2 keer te wandelen en dit steeds met een aantal minuten uit te breiden.

U mag met uw kinderen of kleinkinderen spelen, maar vermijd optillen of dragen. Pak lichte hobby's weer op, zoals schilderen, puzzelen en het doen van gezelschapsspelletjes. Vermijd zwaar huishoudelijk werk zoals schoonmaken, stofzuigen of het koken van een grote maaltijd.

Let er op dat u geen zware objecten tilt of verplaatst.

Probeer actiever in en rondom het huis te worden. Let er op dat u geen zware objecten tilt of verplaatst. Loop gerust naar de lokale supermarkt of winkels als deze dichtbij zijn. Ga door met dagelijkse, lichte wandelingen buitenshuis en bouw deze geleidelijk op.

Ga gerust naar de supermarkt, maar duw de winkelwagen nog niet, vooral niet wanneer deze vol is. U mag gaan winkelen in het dorp/de stad, maar draag daarbij geen zware tassen.

Het is goed om in deze periode de meeste dagelijkse bezigheden die u voor de operatie deed weer op te pakken. Daarnaast kunt u ongeveer 5 km wandelen. Voer bezigheden eventueel in stappen uit.

Stofzuigen is mogelijk. U mag een winkelwagen voortduwen, zelfs als deze vol is. Probeer alle activiteiten geleidelijk op te bouwen en actief te blijven. Bespreek specifieke activiteiten zoals fietsen en zwemmen met uw fysiotherapeut.

PROTOCOL.

Leefregels doornemen. Aangeven dat er vaak enkele maanden nodig zijn om weer volledig aan te sterken. Bij problemen en koorts tot een week na ontslag contact opnemen met de afdeling, Bij problemen later dan de week na ontslag dient er contact opgenomen te worden met de huisarts. Tenminste 6 weken geen deelname aan verkeer, niet fietsen, niet autorijden. Voorkomen van asymmetrische belasting van de thorax. Belang benadrukken van langzaam uitbreiden lichaamsbeweging. Voor uitgebreidere informatie over leefregels verwijzen naar digitale voorlichting.

2312
Bewegen: Maak iedere dag een wandeling en breidt deze langzamerhand steeds uit.

De eerste 6 weken na de operatie mag u: geen zware dingen tillen; niet de hond uit laten; niet fietsen (hometrainer mag wel); niet zwemmen; geen auto rijden; geen zware huishoudelijke werkzaamheden verrichten.

 © 2024. The Author(s).

Na zes weken is de genezing van het borstbeen zo ver gevorderd dat de meeste dagelijkse handelingen weer verricht kunnen worden.

Bewegen. De algemene richtlijn voor bewegen is dat iedereen minimaal een half uur per dag, op minstens vijf dagen van de week, matig intensief beweegt. Dit hoeft niet perse sporten te betekenen. Bewegen is ook wandelen, zwemmen of fietsen. Het is belangrijk de beweging rustig op te bouwen en goed naar uw lichaam te luisteren. U mag bij het bewegen gerust voelen dat u het warm krijgt, uw hart sneller klopt en u dieper moet ademhalen. U gaat te ver als u geen gesprek meer kunt voeren, druk op de borst krijgt, kortademig wordt of een lange tijd nodig heeft om weer op adem te komen. Na maximaal een half uurtje rust moet u zich weer fit voelen. Voor meer informatie kunt u contact opnemen met de hartrevalidatieverpleegkundige.

Sport. Of en wanneer u weer kunt gaan sporten hangt van uw persoonlijke situatie af. Wat is er precies gebeurd, wat is de behandeling geweest en welke sport(en) wilt u gaan beoefenen. In de meeste gevallen kunnen patiënten tot bepaalde hoogte hun oude bezigheden weer oppakken. De cardioloog ziet u liever op recreatief niveau sporten, dan op competitie niveau. Bergwandelen en diepzee duiken zijn ook sporten, die u het best kunt bespreken met uw behandelaar. Het is belangrijk om langzaam op te bouwen, piekbelasting te voorkomen en goed naar uw lichaam te luisteren. Bent u niet zeker wat u in uw geval kunt doen, overleg dan met de hartrevalidatieverpleegkundige, uw huisarts of cardioloog. www.hartstichting.nl/hartziekten/angina-pectoris

Sauna. Bent u gewend om regelmatig naar de sauna te gaan, is het meestal geen probleem om dat te blijven doen. Belangrijk is wel dat u uw lichaam weer de kans geeft om eraan te wennen. Dit houdt in dat u het beste kunt beginnen in een sauna van 60 of 70 graden. De afkoeling kan beter meer geleidelijk worden bewerkstelligd. Eerst naar buiten, of onder de lauwe douche, die eventueel kouder gedraaid kan worden. Snelle, extreme afkoeling wordt afgeraden. Bij twijfel kunt u het navragen bij uw behandelaar.

Voldoende bewegen. We brengen steeds meer tijd van ons leven zittend door: voor de tv, achter de computer, in de auto en op kantoor. Om allerlei redenen lijkt het sporten er bij veel mensen telkens niet van te komen. Toch weten we vrijwel allemaal dat bewegen erg goed is voor de gezondheid .Het houdt ons fit en het speelt een grote rol in het voorkomen van ziekten en aandoeningen, waaronder hart- en vaatziekten, diabetes en kanker. Ook hee bewegen een positief e ect op uw geestelijke gezondheid. Door te bewegen zit u lekkerder in uw vel en daarnaast zijn sociale contacten goed voor u. De Nederlandse Norm voor Gezond Bewegen (NNGB) adviseert volwassenen om per dag minimaal 30 minuten matig intensief te bewegen. Doe dit op minstens 5, maar bij voorkeur 7 dagen per week. Wat "matig intensief" bewegen is verschilt per leeftijdsgroep. Voor volwassenen onder de 55 jaar is dit bijvoorbeeld wandelen met een snelheid van 5 km per uur of fietsen met een snelheid van 15 km per uur. Voor 55-plussers is wandelen met een snelheid van 4 km per uur al "matig intensief." De NNGB moet gezien worden als een ondergrens. Met meer inspanning kan meer gezondheidswinst worden behaald. Voor wie zijn fitheid wil verbeteren is een dagelijks half uur matig intensief bewegen onvoldoende. Voor mensen die willen afvallen of die na een afvalpoging op gewicht willen blijven wordt aanbevolen om minimaal 45-60 minuten per dag matig intensief te bewegen. Overigens hoeven die minuten niet per se in één keer gehaald te worden: met 2×30 of 3×20 minuten kom je ook op 60 minuten uit. Afspreken om regelmatig samen met iemand te gaan wandelen of fietsen kan helpen om van een goed voornemen een goede gewoonte te maken.

2491

Het is belangrijk om in de aanloop naar de operatie te blijven bewegen binnen uw mogelijkheden. Het is verstandig om te kiezen voor een activiteit die niet te belastend is en die u langere tijd vol kunt houden. Denk hierbij aan wandelen of fietsen.

Conditie verbeteren. Voor het behoud van uw conditie is het streven ten minste 30 minuten per dag gedurende vijf dagen in de week te bewegen, waarbij uw hartslag toeneemt en uw ademhaling iets verdiept. In het beweegadvies leest u hoe u uw conditie kunt opbouwen. Wandelen en fietsen op de hometrainer zijn geschikte activiteiten voor de eerste zes weken van uw herstel.

Wandelen Uw wandeltempo wordt bepaald door uw ademhaling. Zorg ervoor dat u tijdens het wandelen nog kunt praten (max. BORG 13). Als dit niet lukt, neem dan regelmatig een korte pauze. De afstand/duur wordt bepaald door de vermoeidheidsgrens. Kunt u per keer minder dan 100 meter lopen, probeer dan de frequentie op te bouwen tot 4-6 keer per dag. Kunt u ongeveer 10 minuten lang wandelen? Dan doet u dat driemaal per dag. Kunt u langer dan 15 minuten wandelen? Dan is tweemaal per dag voldoende. Probeer 2- 6 maal per dag te lopen, afhankelijk van de afstand en de duur.

Fietsen of fietsen op de hometrainer (Na een hartoperatie mag u de eerste zes weken alleen op een hometrainer fietsen). De weerstand, snelheid en duur worden bepaald door de ademhaling. Zorg ervoor dat u tijdens het fietsen nog kunt praten (max. BORG 13). Kies een weerstand waarop u ontspannen kunt trappen (stand 1-3 of 20 tot 60 Watt). Snelheid: Tussen 10-20 km per uur/50–60 omwentelingen per minuut. Als dit goed gaat, kunt u de snelheid verhogen naar 20 of 25 km/uur. Duur 5 minuten, als dit goed gaat, kunt u dit uitbreiden naar 10 of 15 minuten. Frequentie: 2 tot 3 keer per dag afhankelijk van de vermoeidheid en de duur.

3366

Ga niet naar een sportschool en neem geen deel aan andere sportactiviteiten totdat de hartrevalidatie is afgelopen.. Vermijd zwaar belastende activiteiten zoals de trap oprennen, sprinten of fietsen. Til nog niet. In de loop van het beweegprogramma leert u wanneer u dit weer kunt.

Patiënt heeft een voorlopig beweegadvies meegekregen voor thuis: Ontdek wat een goed moment op de dag is om actief te zijn. Verspreid activiteiten over de dag en neem genoeg pauzes. Beweeg in je eigen tempo, kies een tempo

 © 2024. The Author(s).

waarbij u kan blijven praten, dan weet u zeker dat de beweging een lage intensiteit heeft. Doe GEEN intensieve activiteiten, dit kunt u merken aan uw ademhaling. U mag van de activiteit zeker niet buiten adem raken, maar moet kunnen blijven praten tijdens de inspanning. Luister naar uw lichaam (vermoeidheid, klachten); pas eventueel uw activiteiten en/of tempo aan. Stop als het niet goed gaat en ga wat langer door als je je nog prima voelt. Wissel regelmatig (liefst elke 20min.) van activiteit zitten/staan/lopen. Probeer 2x daags een stukje te wandelen. Beweeg met uw partner of een vriend/kennis, als u dat prettig vindt. Bij klachten volgt u het meegekregen advies van uw cardioloog op

Over het algemeen is het goed om een sport te kiezen waarbij je ieder moment het tempo kunt aanpassen, zoals wandelen. Als je dit dagelijks 10 minuten tot een half uur doet, verbetert je conditie al flink.

Patiënt heeft een voorlopig beweegadvies meegekregen voor thuis: Ontdek wat een goed moment op de dag is om actief te zijn. Verspreid activiteiten over de dag en neem genoeg pauzes. Neem tijdig rust en ontspan; wissel rust en inspanning af. Beweeg in je eigen tempo. Luister naar uw lichaam (vermoeidheid, klachten); pas eventueel uw activiteiten en/of tempo aan. Tijdens inspanning is het belangrijk dat u een gesprek kunt blijven voeren, dan weet u dat de intensiteit laag is. Beweeg met uw partner of een vriend/kennis, als u dat prettig vindt. Start met korte afstanden wandelen, ga bijvoorbeeld een blokje om. Breid het wandelen uit: eerst de frequentie (ga vaker wandelen), daarna de loopafstand. U mag voelen dat u bij inspanning wat dieper gaat ademen, maar u mag niet buiten adem raken. Stel zware (huishoudelijke) activiteiten de eerste 6 weken nog uit. Denk daarbij aan stofzuigen, de tuin spitten en de auto wassen. Begin na 2 weken kalm aan met buiten fietsen als u geen klachten heeft. Overleg met uw cardioloog of casemanager hartrevalidatie/fysiotherapeut als u twijfelt of nog klachten heeft. Breid het fietsen rustig uit; eerst de frequentie (ga vaker fietsen), daarna de fietsafstand. Bij klachten volgt u het meegekregen advies van uw cardioloog op.

Over het algemeen is het goed om een sport te kiezen waarbij je ieder moment het tempo kunt aanpassen, zoals wandelen. Als je dit dagelijks 10 minuten tot een half uur doet, verbetert je conditie al flink.

Beweegadvies voor thuis: Ontdek wat een goed moment op de dag is om actief te zijn. Beweeg in je eigen tempo, kies een tempo waarbij u kan blijven praten, dan weet u zeker dat de beweging een lage intensiteit heeft. Doe GEEN intensieve activiteiten, dit kunt u merken aan uw ademhaling. U mag van de activiteit zeker niet buiten adem raken, maar moet kunnen blijven praten tijdens de inspanning.

Wissel regelmatig (liefst elke 20min.) van activiteit zitten/staan/lopen. Probeer 2x daags een stukje te wandelen. Beweeg met uw partner of een vriend/kennis, als u dat prettig vindt

4078

Lichamelijke inspanning. Het doen van lichamelijke inspanning beschadigt uw hart niet. Het verrichten van (huishoudelijke) werkzaamheden is geen probleem. Wel moet u taken waar u vermoeid of kortademigheid van wordt aanpassen, zodat dat u hier niet te veel last van heeft.

Fietsen. Na ontslag uit het ziekenhuis kunt u langzaam uw activiteiten gaan opbouwen. Wij adviseren u om eerst te starten met het wandelen van korte afstanden (bijvoorbeeld een blokje om). Breid dit wandelen eerst vaker uit, daarna mag u de afstand uitbreiden. Als dit goed gaat mag u het fietsen, net als wandelen, rustig opbouwen op vlak terrein. Als u een hartoperatie heeft gehad mag u pas na de fietstest van de hartrevalidatie weer gaan fietsen. U mag wel onbelast fietsen op de home trainer.

Sportactiviteiten. Tijdens het hartrevalidatietraject geeft de fysiotherapeut u gericht advies hoe u uw inspanning kunt opbouwen en wat uw grenzen zijn met bewegen en sporten. Ook wordt er een fietstest gedaan om de belastbaarheid van uw hart te meten. Meestal kunt u uw sportactiviteiten aan het einde van uw hartrevalidatietraject gewoon weer oppakken.

Ook adviseren wij u bij slecht en guur weer binnen te blijven. Lichamelijke inspanning beschadigt uw hart niet

Wandelen. Wandelen is een gezonde vorm van bewegen. Wij adviseren u om te starten met korte afstanden te wandelen (een blokje om). Dit mag u steeds verder uitbreiden, eerst vaker per dag en daarna langere afstanden. Denk er wel aan dat u ook weer terug moet.

Fietsen/ sportactiviteiten. Na een hartoperatie mag u na de fietstest van de afdeling Hartrevalidatie weer fietsen. U mag ook licht trainen op de hometrainer. Tijdens het hartrevalidatietraject geeft de fysiotherapeut u adviezen hoe u uw activiteit kunt opbouwen en wat uw grenzen zijn bij het bewegen en sporten. Ook wordt een fietstest afgenomen om te meten hoever u uw hart kunt belasten. Meestal kunt u aan het einde van uw hartrevalidatietraject uw sportactiviteiten gewoon weer oppakken.

Sauna. U mag gewoon de sauna bezoeken. Wel is het belangrijk dat de afkoeling langzaam gebeurt, door bijvoorbeeld eerst lauw te douchen en het water langzaam steeds kouder te laten worden. Snelle afkoeling raden we af.

Instructies voor dagelijks bewegen

Hoe lang en intensief u een activiteit doet, bepaalt de mate van inspanning. Meestal wordt de activiteit intensiever als u sneller beweegt. Om uw conditie op te bouwen, voert u de activiteiten op. Dat betekent dat u de oefeningen langer en intensiever gaat doen.

Het is belangrijk dat u ernaar streeft dat elke training vermoeiend aanvoelt (matig intensief is). Het beschrijven van uw gevoel van vermoeidheid wordt uitgelegd in de borgscore (zie Beweegrichtlijn en borgscore); u streeft tijdens het bewegen naar een Borgscore 12 tot 13. Met deze intensiteit van bewegen kunt u nog een gesprek voeren tijdens het bewegen, zonder dat u hierbij hoeft te hijgen. Zo leert u tijdens het traject voelen hoe intensief u moet bewegen, ook zonder de hartslagmeter.

Naast wandelen en fietsen kunt u eventueel ook andere bewegingsvormen kiezen om u matig intensief in te spannen. Bijvoorbeeld: traplopen (langzaam); hond uitlaten; ramen

lappen; rustig zwemmen; cardiofitness; paardrijden; roeien; volleybal; badminton. Voor een goede begeleiding is het meten van de hartfrequentie tijdens een activiteit of training nodig. Matig intensief bewegen komt over het algemeen overeen met een hartfrequentie van 55-70% van uw maximale hartfrequentie (HFmax). Deze waardes vertaalt de fysiotherapeut naar uw hartfrequentie-trainingszone. Uw hartfrequentie wordt tijdens alle activiteiten gemeten door een sensor op uw onderarm.

Wat u de eerste 6 weken niet mag is: zwaar tillen, stofzuigen, tuinieren, timmeren, zwaar sporten, zwemmen, ramen zemen en dergelijke

Wees voorzichtig bij alle handelingen die u verricht waarbij u eenzijdig (onverwachts) kracht zet, bijvoorbeeld het uitlaten van een hond, vasthouden aan een stang in openbaar vervoer en dergelijke

Wel is het belangrijk om te werken aan uw conditioneel herstel. U kunt dit doen door dagelijks een wandeling te maken met een afstand die u goed kunt overzien. Breidt dit langzaam uit, en ga de eerste keren niet alleen op stap. Verder mag u gebruik maken van een hometrainer en traplopen.

Na 6 weken is het borstbeen voldoende hersteld en mag u uw armen weer volledig gebruiken. Wanneer dit teveel pijnklachten geeft, wacht dan tot een week later en probeer het voorzichtig nogmaals.

Is uw gehele borstbeen geopend tijdens de operatie? Dan moet u zich houden aan onderstaande leefregels. Het duurt ongeveer 4 tot 6 weken voordat het bot volledig aan elkaar is vastgegroeid. Daarom moet u zich de eerste 6 weken na uw operatie houden aan de volgende regels:

U mag niet fietsen of zelf autorijden. U mag niet zwaarder tillen dan 2 kilo. U mag alleen op uw rug slapen. Alles wat u tilt, moet u synchroon tillen. Dit betekent dat u met beide armen evenveel gewicht tilt, zodat er op beide helften van het borstbeen gelijke druk kom te staan. Zo voorkomt u dat uw borstbeen mogelijk loslaat. Niet stofzuigen of de hond uitlaten. Dit omdat dit asynchrone activiteiten zijn. Bij deze activiteiten is de druk op beide helften van het borstbeen niet gelijk, waardoor er een kans bestaat dat het borstbeen los laat.

Het is belangrijk om aan uw conditie te blijven werken en te blijven bewegen. We raden u aan om elke dag een stukje te wandelen. Bouw dit rustig op en luister goed naar uw lichaam. Neem op tijd rust. Dit kan betekenen dat u overdag even slaapt als uw lichaam dat nodig heeft.

Tijdens uw herstel zult u goede en slechte dagen hebben. Het is niet erg als het herstel op de ene dag wat beter gaat dan de andere dag. Het belangrijkste is dat u over een langere tijd verbetering ziet.

4210

PROTOCOL 1.Leefregels sternotomie. Eerste 6 weken na de operatie: Niet teveel asymmetrische anteflexie (teveel bovenwaarts grijpen naar voorwerpen). Symmetrisch anteflecteren mag wel (niet met de handen achter het hoofd zitten) Geen extreme romprotaties. Geen plotselinge versnelde bewegingen in thoraxregio. Dus geen ramen zemen, stofzuigen etc. Niet meer dan 5 kilogram tillen. Bij uitzonderingen (opstaan uit de stoel, touwtje aan bed om uit bed te komen): symmetrisch kracht verdelen. Patiënten mogen op hun zij slapen (als de drains eruit zijn + geen broos sternum hebben (dit is te lezen bij OK-verslag)) als zij niet op hun rug kunnen slapen. Via HNP methode op hun zij laten komen. Als zij niet benauwd worden / geen pijn hebben, mogen ze op hun zij blijven. Anders toch terug.

5892

Hiervoor heeft u een drietal "houvast-punten": Praattempo ⍰ Tijdens het bewegen nog kunnen praten, 5-7 dagen per week 30 minuten lang. MET's ⍰ Metabole Equivalent (maat voor de hoeveelheid energie die wordt verbruikt, zie tabel andere zijde). Norm: - jonger dan 55 jaar tussen de 4 - 6,5 MET's - ouder dan 55 jaar tussen de 3 - 5 MET's. U voldoet aan de Nationale Norm voor Gezond Bewegen wanneer u een activiteit kiest die voldoet aan de normwaarde en deze activiteit 5-7 keer in de week 30 minuten doet. BORG ⍰ De BORG-score is een goede manier om aan te geven hoe zwaar u een activiteit of de inspanning vindt. Het getal dat overeenkomt met een matig intensieve inspanning is BORG 11–12. Op basis van deze drie "houvast-punten" blijkt dus dat u al vrij snel voldoet aan de Nationale Norm voor Gezond Bewegen en dat u daarmee één van de risicofactoren voor Hart- en Vaatziekten zelf onder controle kunt houden.

Voor het verbeteren van de conditie zult u het lichaam een extra prikkel moeten geven door middel van zwaardere intensieve lichaamsbeweging: 3x per week 20 minuten aaneengesloten met een BORG van 13-15. Niet goed meer kunnen praten vanwege hoge ademfrequentie (snelle ademhaling).

Sport (bewegen) in het algemeen. U kunt beter nog niet sporten, zeker niet in wedstrijdverband. Langzaam de conditie weer gaan opbouwen. (Het beweegprogramma volgt mogelijk nog). Heeft u zich ingespannen en blijft u de hele dag moe, dan heeft u teveel gedaan.

Lopen. Probeer elke dag minstens één keer naar buiten te gaan. Probeer steeds wat verder te lopen. Houd rekening met kou en harde wind. Vraag of iemand met u mee wil lopen als u zich onzeker voelt.

5949

Naar buiten. Als het weer dit toelaat, mag u na uw ontslag gewoon naar buiten.

U mag de eerste zes weken NIET meer dan 1 kg tillen (u mag dus ook geen kinderen tillen); de hond uitlaten; in bad; zwemmen en zonnen.

Sport. Wacht met sporten tot na de hartrevalidatie. Vraag zo nodig advies aan de fysiotherapeut.

Binnen blijven. Na uw ontslag uit het ziekenhuis, mag u naar buiten als de weersomstandigheden dit toelaten.

6048

De patiënt mag de eerste week na de operatie niet zwaar tillen, zwaar huishoudelijk werk verrichten, sporten, fietsen en autorijden. Verder dient de patiënt zo min mogelijk trap te lopen.

 © 2024. The Author(s).

Adviezen voor thuis. Wanneer u thuis bent zult u zich langzaam aan wat beter voelen. De ene dag gaat het wat beter dan de andere dag. Doe het thuis eerst rustig aan. Het is belangrijk om uw krachten langzaam op te bouwen.

Beperk u in eerste instantie tot wandelen. Span u 10 minuten in. Bijvoorbeeld wandelen of licht huishoudelijk werk. Neem daarna 30 minuten rust. Nadien moet u hersteld zijn. Gaat dit goed, dan kunt u de inspanning telkens met 5 minuten uitbreiden. Het is belangrijk om tussendoor voldoende rust te nemen.

7037

Langzaam opbouwen. Enkele weken na de operatie gaat u zich geleidelijk aan beter voelen. Dat herstel gaat op en neer: de ene dag voelt u zich heel goed, de volgende wat minder. Wees zuinig op uw energie, ga niet tot het uiterste. Uw lichaam geeft vanzelf aan wanneer het genoeg is. Bouw uw krachten langzaam op, bijvoorbeeld door steeds iets verder te wandelen en te fietsen. Zo merkt u dat u steeds sterker wordt en weer op uw lichaam kunt vertrouwen. Waarschijnlijk gaat u zich hier ook geestelijk beter door voelen.

Meer bewegen. Om het risico te verminderen op ziekten zoals hart- en vaatziekten is het belangrijk dat u voldoende beweegt. Het is belangrijk dat u dit op zijn minst 5 dagen per week doet. Maar nog beter is het wanneer u elke dag voldoende in beweging bent.

Voldoende beweging houdt in dat u ten minste 5 dagen in de week 30 minuten per dag met diverse activiteiten lichamelijk actief bent. U kunt bijvoorbeeld wandelen, fietsen of zwemmen. Maar ook zwaarder huishoudelijk werk, zoals ramen lappen, stofzuigen en tuinieren vallen onder actief bewegen. Voldoende beweging verkleint niet alleen de kans op ziekten, maar houdt ook uw conditie op peil.

De eerste 6 weken tot aan de hartrevalidatie is het van belang dat u in beweging blijft. Na uw verblijf in het ziekenhuis komt u thuis en is het belangrijk dat u de inspanning langzaam uitbereid. Het is hierbij van belang dat u goed naar uw eigen lichaam luistert. Denk hierbij aan de vermoeidheid, duizeligheid en kortademigheid die kan optreden. Dit kunt u onder andere doen aan de hand van de borgschaal.

Wat kunt u zelf doen? Trainen van het uithoudingsvermogen door te wandelen of hometrainen.

Wandelen (buiten of op een loopband). Wandel de eerste paar dagen na thuiskomst uit het ziekenhuis elke dag 5 tot 10 minuten. Luister hierbij goed naar u lichaam en kijk hoe “zwaar” u de inspanning vond. Hierbij is een borgschaal tussen 11-13 geadviseerd. Breid het wandelen elke dag uit met 1 à 2 minuten.

Probeer toe te werken naar 1x daags 30 minuten te wandelen. Zo voldoet u aan de Nederlandse Norm Gezond Bewegen.

Hometrainer. Indien u over een hometrainer beschikt mag u hierop fietsen. Belangrijk is dat u op een lage weestand gaat fietsen. Start met 2×3 minuten op een lage weerstand. Bereid dit elke dag met of de duur of de zwaarte te verhogen. Luister hierbij goed naar uw lichaam en kijk hoe “zwaar” u de inspanning vond. Hierbij is een borgschaal tussen 11-13 geadviseerd.

Oefeningen ter versterking van beenspieren. Deze oefeningen zorgen ervoor dat uw spierkracht behouden of zelfs sterker wordt in de grote spieren van de benen. Ook zorgt het ervoor dat u minder stijf wordt en weer makkelijker kan lopen. Deze oefeningen kunnen ervoor zorgen dat u zich wat duizelig of kortademigheid voelt, wees hierop alert en pak voldoende rust tussen de oefeningen. Als u merkt dat de oefeningen lichter aanvoelen kunt u het aantal herhalingen verhogen

Oefeningen ter ontspanning van nek-schouder-rompspieren. Door deze oefeningen regelmatig te doen kunt u de (spier)pijnklachten rondom het borstbeen, nek- en schouderregio gunstig beïnvloeden. Ze hebben ook invloed op het herstel van de longen. Deze oefeningen kunnen wat rek op uw borstbeen(wond) geven, maar het mag geen pijn doen. Let tijdens het zitten, staan en lopen op een rechte houding. Probeer rustig door te blijven ademen tijdens de oefeningen. Voer de oefeningen uit in zit op de bedrand of in de stoel. Zorg dat u ontspannen zit en rechtop zit.

7041

Binnen blijven: Na ontslag is het belangrijk om nog een week binnen te blijven, aangezien uw weerstand nog niet op peil is.

Sporten: In overleg met uw cardioloog en fysiotherapeut.

Probeer iedere week minimaal 2,5 uur matig intensief te bewegen of te sporten.

7096

Wandelen. Het maken van een wandeling is toegestaan en raden wij u zelfs aan. Begin bijvoorbeeld met dagelijks een aantal keren een korte wandeling (tien minuten) en breidt dit langzaam uit. Let er wel op dat als u met een (grote) hond gaat wandelen, deze niet met ú uitgaat in plaats van u met de hond. Plotseling trekken aan de riem kan extra druk op het nog niet genezen borstbeen geven. Vraag in dat geval vooraf aan de fysiotherapeut wanneer u weer mag proberen uw hond uit te laten. Ook het lopen met één stok of één kruk is de eerste vier weken niet toegestaan, omdat de beide helften van het borstbeen ten opzichte van elkaar kunnen gaan verschuiven.

Sporten. In verband met de genezing van het borstbeen mag u de eerste zes weken na de operatie nog niet sporten. Opnieuw starten met sporten is afhankelijk van uw herstel en conditie van vóór de operatie. Overleg tijdens de controleafspraak met uw cardioloog wanneer u kunt beginnen met sporten. Vooral als u wilt zwemmen, moet u dit eerst met hem bespreken. Over het algemeen kunt u uw conditie langzaam weer opbouwen naar uw sportniveau van vóór de operatie. Er zijn mogelijkheden om dit via een hartrevalidatieprogramma te doen, bespreek dit met uw cardioloog.

8022

Binnen blijven. Het is verstandig om na uw ontslag nog zeven dagen binnen te blijven. Zo kan uw weerstand weer op peil komen. Na die tijd kunt u – bij goed weer – even naar buiten en dit langzaam uitbouwen

8858
Voldoende bewegen verkleint direct de kans op hart- en vaatziekten. Door meer te bewegen wordt de bloeddruk lager en neemt het gewicht af. Het zorgt voor een verbeterde stofwisseling en dat zorgt weer een betere balans in de bloedsuikers. Om de kans op hart- en vaatziekten te verkleinen is het voldoende om wekelijks tweeenhalf uur matige tot redelijke inspanning te leveren. Wat matige tot redelijke inspanning is, verschilt van persoon tot persoon. Wel gelden voor iedereen de volgende vuistregels:

De ademhaling versnelt, maar u raakt niet buiten adem. Het hart gaat er sneller van kloppen. U bent binnen korte tijd weer op uw normale ademhaling- en hartslagniveau terug.

8935
Bewegen. Regelmatig bewegen tijdens en uw behandeling kan een belangrijke bijdrage leveren aan uw conditie en uw herstel. Vraag uw behandelaar welke activiteiten voor u geschikt zijn.

Activiteiten (onderstaande adviezen op geleide van de pijn). Opstaan vanuit de stoel met gebruik armleuning: toegestaan. Verplaatsen in bed, opdrukken met de armen achterwaarts: toegestaan. Verplaatsen in bed, opdrukken met de armen voorwaarts: toegestaan. Komen tot zit op rand bed met ondersteuning armen: toegestaan. Tillen: gelijkmatige belasting van de borstbeen, tot 5kilogram.

Krachttraining bovenste extremiteit: na 6 weken gedoseerd opbouwen op geleide van de pijn, na 3 maanden volledig toegestaan. Unilaterale belasting: eenzijdig belasten (bijvoorbeeld steunen op één arm)

Leefregels belasten borstbeen na sternotomie. Restricties gelden voor de eerste 6 weken na sternotomie. Na 6 weken mag de belasting en mobilisatie op geleide van klachten uitgebreid worden.

Algemene opmerkingen 1. Voorkom asymmetrische belasting. 2. Een in elkaar gezakte houding geeft extra drukbelasting op het borstbeen. 3. Géén bovenhandse trek- en drukkrachten. Bovenhands = hoger dan 90 graden heffen van de armen)

Hulpmiddelen. Stok: niet toegestaan tot zes week na operatie (i.v.m. unilaterale belasting*). Vierpoot: niet toegestaan tot zes week na operatie (i.v.m. unilaterale belasting*). Rollator: toegestaan, mits voldoende beenspierfunctie, beoordeeld door de fysiotherapeut

9042
Plotselinge overgang van warme lucht naar koude lucht kan een onprettig gevoel geven. Dus kleedt u zich bij koud weer goed aan (sjaal om) en neem de tijd om te acclimatiseren. Daarom wordt sauna bezoek ook de eerste tijd afgeraden.

De eerste 6 weken staan in het teken van de herstelperiode. Dit is geen periode om intensief te trainen. Het is belangrijk dat u weer zo snel mogelijk in uw eigen dagritme komt. Dat lukt niet direct. Bouw uw activiteiten rustig op, bijvoorbeeld: Week 1 en 2: U mag beginnen met 2 keer per dag buiten te wandelen. Probeer zelf te voelen hoe lang dit goed is voor u. Voor de één is dit maximaal 5 minuten. Voor een ander kan dit een half uur zijn. Neem iemand mee als u voor het eerst gaat wandelen. Neem ook uw mobiele telefoon mee. Dit kan u wat meer zelfvertrouwen geven.

U mag de eerste 6 weken alleen met beide handen tillen. De eerste week mag het gewicht dat u tilt maximaal 2 kilo zijn. Het gewicht mag elke week met 2 kilo worden verhoogd.

Hef uw armen de eerste 6 weken niet boven uw schouders. Houd rekening met hoge keukenkastjes of een kledingkast. Na de 6 weken kunt u dit opvoeren en mag u wel boven schouderhoogte bewegen. Voorkom dat het schoudergewricht vast gaat zitten. U kunt wel zelf uw haren wassen of een T-shirt aantrekken.

Als u in de eerste 2 weken weinig moeite hebt gehad met de genoemde activiteiten, dan kunt u wat meer gaan doen. U kunt uw wandeling naar eigen inzicht uitbreiden naar 45-60 minuten en verder. Blijf ondertussen naar uw lichaam luisteren. Na uw controleafspraak bij de cardioloog mag u beginnen met een stukje fietsen. Bouw ook dit langzaam op. Begin met fietsen als het goed weer is en op een zo rustig mogelijke weg. U mag, na toestemming van de cardioloog, na 6 weken ook weer zelf autorijden. Na 6 weken en in overleg met uw cardioloog geldt de algemene Nederlandse Norm Gezond Bewegen. Zie hiervoor het einde van deze folder. Blijf ondertussen goed letten op uw lichamelijke mogelijk- en onmogelijkheden.

Omdat het borstbeen nog niet genezen is, mag u de eerste 6 weken geen zwaar huishoudelijk werk doen zoals, stofzuigen, ramen zemen, boodschappen tillen, grasmaaien et cetera. Tevens raden wij u af om honden aan de riem uit te laten en om kinderen op te tillen. Probeer ook om de eerste 6 weken op uw rug te slapen. Als u hierdoor niet kunt slapen, mag u wat op de zij draaien. Dit mag alleen zolang u hier geen pijn bij heeft.

PROTOCOL Fase V post-operatief

Mobiliseren uitbreiden met traplopen op basis van de belastbaarheid van de patiënt en onder controle van de saturatie en hartslag en klinische blik.

Tevens instructie geven voor thuis: leefregels ten aanzien van sternumbelasting en het opbouwen van de belasting (zie brief Activiteiten na ontslag na een open hartoperatie). Het gaat om de volgende leefregels: U dient de eerste 6 weken na de operatie middels de teugel uit bed te komen zoals u dat geleerd heeft in het ziekenhuis. U dient de eerste 6 weken na de operatie niet op uw handen te steunen bij bijvoorbeeld het opstaan van een stoel of uit bed. Het tiladvies voor de eerste 6 weken luidt: symmetrisch tillen (met beide handen). De belasting mag per week opgevoerd worden met 2 kg. Gedurende de eerste 6 weken dient u op uw rug te slapen. Desgewenst kunt u in een hoek van 30 graden liggen, mits goed gesteund. U mag alleen op de zij slapen als dit geen pijn geeft aan het borstbeen. Bouw uw activiteiten rustig aan op. De eerste 6 weken mag u geen zwaar huishoudelijk werk doen. Breidt uw loopafstand rustig uit. U mag thuis op de hometrainer zonder belasting. Hef uw armen gedurende de eerste 6 weken niet boven schouderhoogte.

 © 2024. The Author(s).

9627
Wandelen. Wandelen mag en is goed voor uw herstel. U mag dit langzaam opbouwen.

Tillen. Til geen zware voorwerpen. Let op bij het tillen van voorwerpen dat u gelijkmatig uw borstbeen belast. Draag een tas dus niet in één hand terwijl de andere hand leeg is.

Sporten. De eerste 6 weken mag u nog niet sporten. Tijdens de hartrevalidatie wordt weer een begin gemaakt met sport- en speloefeningen. Daarna mag u het sporten weer langzaam opbouwen.

Hobby's. Lichamelijke belastende hobby's mag u weer oppakken maar luister hierbij naar uw lichaam en klachten.

Op uw zij liggen. U mag op uw zij liggen wanneer dit mogelijk is. U moet comfortabel kunnen liggen.

Aandachtspunten voor het sporten: Zorg ervoor dat u gegeten hebt voordat u komt sporten. Zorg ervoor dat u uw medicijnen ingenomen hebt voordat u komt sporten. Bij koorts mag u niet komen sporten. Voordat u begint met sporten en als u klaar bent, moet u uw handen wassen. Dit in verband met uw hygiëne en die van andere patiënten.

In de eerste zes weken: Activiteiten mogen verricht worden, zolang de handelingen geen pijn geven en de bovenarmen zoveel mogelijk langs het lichaam (in de buis) worden gehouden. U mag op uw zij slapen, zolang u hierbij geen pijn ervaart. U mag voorwerpen tillen zonder gewichtsbeperking, zolang u dit altijd met 2 handen tegelijk doet en hierbij geen pijn ervaart. U mag niet vaker dan 3 keer per dag de trap op- en aflopen. U mag niet trainen.

9636
Conditie opbouwen de eerste 6 weken. Na ontslag uit het ziekenhuis hoeft u nog niet naar een fysiotherapeut om verder te trainen. Eerst moet het borstbeen herstellen. Daarom start de periode van hartrevalidatie pas 6 weken na de operatie.

Bij het opbouwen van de conditie na ontslag uit het ziekenhuis is het verstandig om met kleine stappen te beginnen.

Kies een matig intensieve activiteit, dit is een lichte vorm van bewegen. Denk hierbij aan wandelen of fietsen op een hometrainer.

Start bijvoorbeeld met vier tot zes keer per dag met een korte wandeling (5-10 minuten). Bouw 1 van deze afstanden uit tot maximaal 30 minuten (aan één stuk lopen) in de eerste 6 weken.

Algemene tips bij bewegen. Let erop dat u zich comfortabel kleedt. Dit betekent dat u gemakkelijk zittende kleding aantrekt. Trek schoenen aan die stevig aan uw voeten blijven zitten en een goed profiel hebben (ook tijdens opname!). Als u een vermindert evenwicht heeft is het van belang om de een hulpmiddel te gebruiken, zoals een rollator. Als u voor de eerste keer na uw ontslag buiten gaat bewegen, is het verstandig om dit samen met iemand te doen. Mogelijk is uw stabiliteit nog niet optimaal en de indrukken die u buiten opdoet of de ongelijke ondergrond kunnen u wellicht nog extra uit balans brengen. Rust is belangrijk, beweeg bijvoorbeeld een half uur en doe daarna een half uur rustig aan.

Hoe intensief beweegt u? Met een borgschaal (zie tabel op de volgende pagina) kunt u bepalen hoe u de inspanning ervaart. Het is een handig hulpmiddel waarbij u een cijfer geeft aan de zwaarte van uw inspanning. Zoek het getal op de schaal dat het best weergeeft hoe u zich voelt direct na uw activiteiten. Vindt u de inspanning redelijk zwaar, dan ervaart u dat als een 13. Als u het minder zwaar vindt dan redelijk zwaar (13), maar wel zwaarder dan tamelijk licht (11) dan geeft u de inspanning het cijfer 12. Probeer te starten met een inspanningsniveau van 12-13 en dit geleidelijk uit te breiden naar 14. Hoger dan 14 is over het algemeen niet nodig.

9909
Voldoende lichaamsbeweging. De Nederlandse Norm voor Gezond Bewegen (NNGB) adviseert volwassenen om per dag minimaal 30 minuten matig intensief te bewegen. Wat "matig intensief" bewegen is, verschilt per leeftijdsgroep. Voor volwassenen onder de 55 jaar is dit bijvoorbeeld wandelen met een snelheid van 5 km per uur of fietsen met een snelheid van 15 km per uur. Voor 55-plussers is wandelen met een snelheid van 4 km per uur al "matig intensief."

Sedentaire Leefstijl en Lichamelijke Activiteit – Rusten

0535
Vermoeidheid. U zult merken dat u zich thuis sneller moe voelt en meer behoefte heeft aan slaap. Het is verstandig ook 's middags even te rusten. Toegeven aan vermoeidheid is raadzaam. Probeer wat regelmaat in uw dag aan te brengen en een dag- en nachtritme op te bouwen.

Week 1: Het is de bedoeling dat u binnen 5 à 10 minuten herstelt van uw inspanning. Pas uw activiteiten aan als dit niet gebeurt. Doe het thuis rustig aan en zorg dat u op tijd rust neemt. Ga de trap rustig op en af. Het is verstandig om halverwege te stoppen om te rusten als u kortademig wordt of ongemak ervaart.

Week 2: Rust weer op tijd uit.
Week 3-4: Probeer rust te pakken wanneer dit nodig is.
Week 5-6: neem tussendoor pauzes als u dit nodig heeft

Deze adviezen zijn slechts richtlijnen. Iedere patiënt heeft een eigen hersteltempo en –niveau. Gebruik uw gezonde verstand en gun uzelf de beste kans op een vlot herstel. In de eerste fase van het herstel kunt u zich vermoeider voelen dan normaal, maar gaat voorbij als u meer conditie en vertrouwen krijgt. Het is goed om te bewegen en dit uit te breiden, maar let erop dat u niet gaat overbelasten.

Verschijnselen van overbelasting zijn: Pijn of druk op de borst. Kortademigheid of abnormale vermoeidheid die niet in verhouding staat tot de geleverde inspanning. Toename van vocht in het gehele lichaam, armen of benen. Onregelmatige hartslag of hartkloppingen. Overmatig zweten, misselijkheid of duizeligheid.

2312
Rust: Tussen de middag kunt u een uurtje rusten indien gewenst. Voer geleidelijk uw lichamelijke en sociale activiteiten op.

 © 2024. The Author(s).

Rust. Na een opname is het niet nodig om uw slaappatroon te veranderen. Een extra middagdutje van maximaal 1 uur, indien nodig, is een goede manier om uw lichaam de rust te geven, die het nodig heeft om te herstellen. Voer uw lichamelijke en sociale activiteiten geleidelijk op, zoals uw lichaam dit aan kan.

2491
Tegelijkertijd is het verstandig om binnen uw inspanningsgrens te blijven. Hierbij kunt u letten op tekenen van overbelasting.

Overbelasting. Indien u tijdens inspanning onderstaande verschijnselen ervaart, heeft u de inspanningsgrens bereikt. U dient dan even rust te nemen. Nemen deze klachten niet af na rust, dan raden wij u aan contact op te nemen met uw huisarts of specialist. Overmatige kortademigheid niet passend bij de geleverde inspanning; Overmatige vermoeidheid niet passend bij de geleverde inspanning; Overmatige transpiratie en bleekheid niet passend bij de geleverde inspanning; Duizeligheid; Pijn op de borst klachten

3366
Verspreid activiteiten over de dag en neem genoeg pauzes

Luister naar uw lichaam (vermoeidheid, klachten); pas eventueel uw activiteiten en/of tempo aan.

Stop als het niet goed gaat en ga wat langer door als je je nog prima voelt

4078
Het is belangrijk dat u het de eerste tijd rustig aan doet. Luister goed naar uw lichaam en houd in de gaten wat u wel en niet kunt. Neem op tijd even rust. Na zeven dagen kunt u langzaam weer wat meer doen. Zorg ervoor dat u niet teveel visite krijgt. Wel moet u taken waarvan u moe of kortademig wordt zo aanpassen dat u hier niet teveel last van heeft.

Ontspannen. Naast voldoende beweging is het ook belangrijk dat uw lichaam tussendoor rust krijgt om te kunnen herstellen. Zo gaan veel mensen in de eerste week thuis vaak halverwege de dag een half uurtje rusten.

4210
Het mobiliseren en het uitbreiden hiervan doet u op geleide van uw klachten zoals pijn, benauwdheid, vermoeidheid en/of duizelingen. Forceer uw lichaam niet, maar luister naar de signalen die uw lichaam geeft en neem tijdig uw rust. Voor een goed herstel van de operatie is het van belang de juiste balans te vinden tussen inspanning en rust.

4794
"Misverstanden" over ontspanning

Ontspannen is gemakkelijk te leren. Dit is zeker niet waar, het kan een lange tijd duren om ontspannen eigen te maken. Naast de instructie in groepsverband, is het belangrijk om ook thuis te oefenen. Het is net als bij het leren van een vreemde taal. Alleen door te praten, te lezen, te oefenen krijgt u de taal onder de knie. Niet door het uit een boekje te leren.

U kunt denken: "Wanneer ik niets voel van het lichaam, dan is het goed." Dit is niet waar, zowel prettige als niet prettige signalen van het lichaam zijn belangrijk om te voelen. Zij geven feedback over hoe het ermee gaat. Deze feedback heeft u o.a. nodig om grenzen te kunnen bepalen, om te ervaren wanneer er spanning in uw lichaam is, maar ook om te weten wanneer u zich prettig voelt of rustig.

Ontspannen moet u niet alleen doen als u spanning voelt. Juist wanneer u toch al uitgerust bent, lukt het het beste. U leert dan gemakkelijker voor u zelf te ervaren hoe ontspanning voelt, zodat u het ook kunt gaan toepassen op momenten dat u wel gespannen bent.

Het primaire doel van ontspanningsoefeningen is niet om tijdens de sessie in slaap te vallen. Het kan u wel helpen om gemakkelijker in slaap te vallen als u slaapproblemen hebt.

5892
Belasting – belastbaarheid (overbelasting). U kunt er uiteraard voor kiezen meer te bewegen dan dat de Nationale Norm voor Gezond Bewegen voorschrijft. Wat dan belangrijk is, is dat u waakt voor overbelasting. Wij hanteren daarbij het model belasting – belastbaarheid.

In dit model staat belasting voor datgene wat u doet en belastbaarheid voor datgene wat u aankan. Idealiter moet dit in evenwicht zijn. Bij overbelasting is dit evenwicht verstoord, de belasting is groter dan de belastbaarheid, oftewel u doet meer dan dat het lichaam eigenlijk aankan.

Overbelasting treedt ongemerkt sneller op dan u denkt. Door uw recente verleden met hartklachten is uw belastbaarheid, mogelijk afgenomen. U zult uw belasting daarop aan moeten passen om het evenwicht te behouden. Het is gebleken dat veel mensen (te) snel hun "oude leventje" weer op willen pakken en daarmee het lichaam te snel teveel belasten in verhouding tot de belastbaarheid. Bij een juiste opbouw van de "conditie" neemt u voldoende rust na een inspanning om het lichaam goed te laten herstellen. U komt dan op een hoger niveau uit dan dat u bent gestart, uw prestatievermogen neemt toe (lijn a). Overbelasting wordt weergegeven met lijn b. U neemt onvoldoende rust na een inspanning en het lichaam herstelt onvoldoende. U komt dan op een lager niveau uit dan dat u bent gestart. Uw prestatievermogen neemt langzaam af, u voelt zich toenemend moe worden, soms al voordat u iets gedaan heeft. Als u dit bij uzelf merkt, neem dan langere tijd rust en bouw uw inspanningen langzamer op.

5949
Rustig aan. Het is belangrijk dat u de eerste tijd rustig aan doet. De oefeningen die u in het ziekenhuis deed met de fysiotherapeut, kunt u wel doen. Luister daarbij vooral goed naar uw lichaam en houd in de gaten wat u wel en niet aankunt. Neem op tijd rust en ga 's middags een uurtje op bed liggen. Na 7 tot 10 dagen kunt u uw activiteiten geleidelijk uitbreiden.

Doe rustig aan. Het is van belang dat u het de eerste tijd nog rustig aandoet. Uw wond (borstbeen bij CABG en aorta operatie) is namelijk nog niet goed genezen. U kunt wel de

 © 2024. The Author(s).

oefeningen doen die u tijdens uw ziekenhuisopname met de fysiotherapeut deed. Luister vooral goed naar uw lichaam en houd in de gaten wat u wel en niet aankunt. Neem op tijd even rust. Ga ‘s middags een uurtje op bed rusten. Na 7 dagen kunt u uw activiteiten weer geleidelijk uitbreiden.

TYPE B DISSECTIE: Bedrust. U mag u zo min mogelijk inspannen. Zo voorkomt u dat u een hoge bloeddruk krijgt en dat de druk in uw borstkas verhoogt. De aorta moet zoveel mogelijk worden ontzien zodat de wand van de slagader weer steviger kan worden. U moet daarom één week bedrust houden. Na deze week maken we een CT-scan om te kijken of de bedrust nog nodig is. Uw behandelend arts stelt vast hoe lang u bedrust nodig hebt.

Lichamelijke conditie. Bij thuiskomst zult u zich nog niet optimaal voelen. Waarschijnlijk is uw concentratievermogen minder dan normaal en is uw lichamelijke conditie nog lang niet op peil. Deze klachten komen vaak voor; ze worden veroorzaakt door de lange operatie, de narcose, het gebruik van de hart-longmachine en alles wat u in het ziekenhuis hebt meegemaakt. Goede voeding, voldoende rust en geduld zijn absolute voorwaarden om uw conditie weer op te bouwen.

Rust. Het is van belang dat u het de eerste tijd nog rustig aandoet, omdat uw wond nog niet goed genezen is. De oefeningen die u tijdens uw ziekenhuisopname met de fysiotherapeut deed, kunt u wel uitvoeren. Luister vooral goed naar uw lichaam en houd in de gaten wat u wel en niet aankunt. Neem op tijd rust en ga ‘s middags een uurtje op bed liggen. Na 7 tot 10 dagen kunt u uw activiteiten geleidelijk uitbreiden.

Spierpijn. Als gevolg van de operatie hebben de meeste patiënten last van spierpijn in de nek, de rug, de ribben en rond de schouder. De spierpijn kan wel 6 tot 8 weken aanhouden en verdwijnt dan geleidelijk.

7037
Langzaam opbouwen. Enkele weken na de operatie gaat u zich geleidelijk aan beter voelen. Dat herstel gaat op en neer: de ene dag voelt u zich heel goed, de volgende wat minder. Wees zuinig op uw energie, ga niet tot het uiterste. Uw lichaam geeft vanzelf aan wanneer het genoeg is. Bouw uw krachten langzaam op, bijvoorbeeld door steeds iets verder te wandelen en te fietsen. Zo merkt u dat u steeds sterker wordt en weer op uw lichaam kunt vertrouwen. Waarschijnlijk gaat u zich hier ook geestelijk beter door voelen.

7041
Rust: Het is belangrijk dat u het de eerste tijd nog rustig aan doet. Luister goed naar uw lichaam, houd in de gaten wat u wel en niet aankunt. Na een week kunt u uw activiteiten wat uit gaan breiden. Wel is het van belang dat u tot de controle bij de cardioloog rustig aan doet.

Een vast ritme is belangrijk voor onze slaap: elke dag op hetzelfde tijdstip naar bed gaan en op hetzelfde tijdstip opstaan. Blijf niet langer dan 8 uur in bed liggen, ook niet in het weekend. Daardoor creëer je een soort jetlag. Lang in bed liggen verstoort de biologische klok. Dit wordt veroorzaakt door het natuurlijke ritme van de biologische klok. Deze is bij de meeste mensen net iets langer dan 24 uur. Licht in de ochtend activeert de biologische klok en zet de klok in “waakstand.” Dit zorgt ervoor dat het ritme 24 uur blijft en niet doorschuift. Zoek daarom in de ochtend lekker veel daglicht op. In de avond is het juist slim om het licht te dimmen, zodat de biologische klok niet in de “waakstand” blijft. Het gebruik van schermen met blauw licht voor het slapengaan kan ertoe leiden dat je lastiger in slaap komt.

Wil je lekker en goed slapen? Creëer dan een goede omgeving. Daarbij is regel nummer 1: gebruik de slaapkamer alleen voor slaap en de liefde. Een goed bed is natuurlijk essentieel voor goede slaap. Zorg er dus in de eerste plaats voor dat je een prettig liggend matras, een goed kussen en fijn beddengoed hebt. Daarna is het belangrijk dat de kamer lekker donker is voordat je gaat slapen. Zorg er ook voor dat je niet teveel wordt afgeleid door omgevingsgeluiden. Oordoppen kunnen een uitkomst bieden! Zet de wekker uit je directe zicht. Het scheelt onrust als je niet steeds kunt zien hoe laat het is. Houd de kamer en je lichaam koel en slaap in iets luchtigs.

Mensen met een goede conditie slapen beter. Probeer iedere week minimaal 2,5 uur matig intensief te bewegen of te sporten. Doe dat liever niet laat op de avond, want dan val je lastiger in slaap. Houd vaste eetmomenten aan. Ontbijt heeft een belangrijke functie voor de biologische klok. Door de darmen in de ochtend van voedsel te voorzien, zet je je lijf in de waakstand. Heb je in de avond nog trek? Dan kan je best nog een lichte snack eten. Bijvoorbeeld een banaan, dadels of melk. Laat de vette snacks liever staan en eet niet meer na 22.00 uur. Ook alcohol, cafeïne en sigaretten kun je beter vermijden voor het slapengaan. Alcohol kan soms helpen om sneller in slaap te komen, maar zorgt over het algemeen voor een onrustige slaap. Cafeïne en nicotine hebben een activerende werking en houden je zenuwstelsel actief; iets wat je liever niet wilt voor het slapengaan.

7096
Spierpijn. Na de operatie is spierpijn in de nek en rug, tussen de ribben en rondom de schouders een normaal verschijnsel. Dit wordt veroorzaakt doordat de randen van het borstbeen tijdens de operatie uit elkaar getrokken zijn om bij het hart te komen. De spierpijn wordt geleidelijk minder, maar kan wel zes tot acht weken aanhouden.

8022
Ontspannen. De eerste tijd heeft u veel rust nodig. De oefeningen die u leerde van de fysiotherapeut kunt u gewoon doen. Luister daarbij wel naar uw lichaam en ga niet te ver. De eerste zeven tot tien dagen is het prima om ’s middags een uurtje op bed te liggen. Daarna pakt u rustig aan meer activiteiten op.

9042
De dag van uw ontslag uit het ziekenhuis is voor u een prettige, maar ook vermoeiende dag. Houd er rekening mee dat u de eerste dagen thuis niet te veel bezoek ontvangt.

 © 2024. The Author(s).

Bij alles wat u doet, kunt u vermoeid raken. Dat is logisch en ook goed. Zorg ervoor dat u niet oververmoeid raakt. Oververmoeidheid kan juist een averechts effect hebben. Dit kunt u direct na inspanning merken, maar soms pas de dag erna. Zie uw lichaam als een batterij. Deze moet u niet helemaal leegtrekken, anders laadt hij minder goed op. Er moet dagelijks een reserve aan energie overblijven. Daarom is het goed om inspanning af te wisselen met het nemen van voldoende rust. Een goede nachtrust is belangrijk voor uw herstel.

Verschijnselen van overbelasting van het hart kunnen zijn: kortademigheid; abnormale vermoeidheid (niet gelijk aan de geleverde inspanning); duizeligheid; flauwvallen; hartkloppingen; snelle hartslag (niet gelijk aan de geleverde inspanning); overmatig transpireren

Als u een van deze klachten hebt, neem dan even rust en wacht af of het minder wordt. Als het niet vermindert, neem dan contact op met het ziekenhuis. Uw herstel gaat niet in een rechte lijn bergopwaarts; een tijdelijke terugval van 1 of meerdere dagen zo nu en dan is heel gewoon.

Week 2: Verdeel uw taken over de dag, niet alles hoeft in één keer af.

9627

Rustig opbouwen. Verlang niet van uzelf dat u elke dag meer moet presteren dan de vorige dag!!! Luister naar uw lichaam en pas uw activiteiten aan. U mag niet meer pijn gaan voelen en niet uitgeput zijn na het doen van een activiteit.

Spierpijn. Na de operatie kunt u spierpijn hebben (nek, rug, ribben en in de schoudergordel). Dit is normaal en kan een lange tijd aanhouden. U mag warmtepakkingen gebruiken zoals een kersenpittenzakje. Dit kunt u combineren met oefeningen voor nek en schouders.

Ontspanning. Als u goed kunt ontspannen, heeft dit positieve effecten voor het herstel op de lange duur. Tijdens de trainingsmodule zult u kennismaken met verschillende vormen van ontspanningsoefeningen. U krijgt ook oefeningen aangeleerd die u thuis zelf kunt doen. Op specifieke indicatie en in overleg met de fysiotherapeut bestaat er de mogelijkheid om een individueel plan van aanpak voor ontspanningstherapie te maken of aan te sluiten bij de ontspanningsoefeningen van de longrevalidanten op vrijdagochtend van 11:00-12:00.

9636

Het is belangrijk dat u goed let op de signalen die het lichaam geeft. Bij inspanning gaat uw lichaamstemperatuur iets omhoog, kunt u gaan zweten, krijgt u een hogere hartslag en gaat u sneller ademhalen. Dit is normaal. Bij de volgende signalen moet u echter wel stoppen met de inspanning: Bij pijn op de borst en pijn in de arm. Als u meer dan normale pijn heeft in het geopereerde gebied. Als uw wond meer vocht produceert dan normaal. Wanneer u last krijgt van abnormale vermoeidheid. Als u kortademigheid heeft die niet in verhouding staat tot de inspanning. Wanneer u duizelig wordt. Als u overmatig zweet. Als u misselijk wordt. Pijn in de uren na het bewegen (napijn) kan een graadmeter zijn voor de intensiteit waarmee bewogen is. Mocht u meer dan 2 uur napijn hebben, dan moet uw inspanning de volgende keer minder zwaar zijn. U mag wel voelen dat u een inspanning heeft geleverd, maar u mag niet overmatig vermoeid zijn.

Sedentaire Leefstijl en Lichamelijke Activiteit – Verkeer

0535

Deelnemen aan verkeer. Afhankelijk van uw herstel kunt u vier tot zes weken na de operatie weer deelnemen aan het verkeer (fietsen of auto). Indien u twijfelt, overleg dan met de cardioloog. Wel kunt u op een hometrainer fietsen. Doe dit zonder of met een minimale weerstand en voer de fietstijd langzaam op van vijf tot maximaal vijftien minuten.

Week 2: Probeer openbaar vervoer te vermijden, maar u mag wel als bijrijder meegaan in de auto (doe altijd een gordel om).

Week 5-6: U mag weer gebruik maken van het openbaar vervoer.

2312

Fietsen en autorijden: Heeft u met wandelen voldoende conditie opgebouwd, kunt u voorzichtig beginnen met fietsen. Gezien de genezing van het borstbeen wordt geadviseerd om pas na zes weken te gaan fietsen. Hetzelfde geldt voor autorijden.

De eerste zes weken mag u geen auto rijden; niet fietsen (hometrainer mag wel);

Fietsen en autorijden: heeft u met wandelen voldoende conditie opgebouwd, dan kunt u voorzichtig beginnen met fietsen. Gezien de genezing van het borstbeen wordt geadviseerd om pas na zes weken te gaan fietsen. Hetzelfde geldt voor autorijden.

Autorijden: Na een bypass- of hartklepoperatie mag u pas na 6 weken weer autorijden, mits er geen complicaties zijn opgetreden die uw rijvaardigheid beïnvloeden. Na een hartoperatie via de zijkant van de borstkas, of via de lies mag u 4-6 weken niet autorijden, mits er geen complicaties zijn opgetreden die uw rijvaardigheid beïnvloeden. Indien u nog regelmatig last hebt van hartklachten, kan uw cardioloog bepalen of u mag autorijden.

Vliegen. Ongeveer vier weken na ontslag uit het ziekenhuis mag u weer vliegen. Mits het geen lange inspannende reis wordt. Daar kunt u beter mee wachten tot uw conditie weer voldoende is. Bij twijfel: informeer eerst bij uw behandelaar.

2491

Autorijden. U mag de eerste vier weken na een infarct en de eerste zes weken na een hartoperatie niet zelf autorijden

3366

Neem nog geen deel aan het verkeer. U mag wel wandelen. Na een hartinfarct of een hartoperatie mag u tenminste vier weken niet autorijden.

4078

Autorijden. Als er geen problemen ontstaan en u kunt zich weer goed concentreren, dan mag u na vier weken weer autorijden. Dit geldt voor alle motorrijtuigen.

 © 2024. The Author(s).

4210

De eerste zes weken: Verder mag u niet zelf autorijden, fietsen en op een (snor)scooter rijden of achterop meerijden; Doe in de auto altijd een gordel om! Ook verzekeringstechnisch bent u dit verplicht.

De eerste zes weken: U mag niet fietsen of zelf autorijden

Fietsen. Blijf de eerste tijd dicht bij huis. Houd rekening met kou en harde wind. 2 weken na een infarct en 6 weken na een hartoperatie mag u weer fietsen. Vraag of iemand met u mee wil fietsen als u zich onzeker voelt.

Autorijden. 4 weken na een hartinfarct en 6 weken na een hartoperatie mag u weer autorijden. (Als u zich zeker genoeg voelt.) Probeer in het begin druk verkeer te vermijden. Rijd in het begin niet te lang achter elkaar. Autorijden is inspannender dan u denkt.

5949

Fietsen en autorijden. U kunt zich de eerste tijd na een aortadissectie type A en/of type B minder goed concentreren. We adviseren u daarom om de eerste 6 weken geen auto te rijden of te fietsen.

Dagelijkse activiteiten: wat mag u de eerste 6 weken NIET? Zelf autorijden of fietsen (dit is vanwege de verminderde concentratie en omdat er bij het remmen druk via uw armen op uw borstbeen komt);

Verkeer. Als gevolg van de ingreep en de narcose kunt u zich de eerste tijd na uw operatie minder concentreren. Daarom adviseren wij u de eerste 6 weken geen auto te rijden of te fietsen.

7041

Autorijden/fietsen: U mag de eerste zes weken niet autorijden of fietsen.

7096

Fietsen en autorijden . Het duurt ongeveer zes weken voordat de wonden als genezen worden beschouwd. Het is dan ook belangrijk dat u in die periode voorzichtig bent met activiteiten als bijvoorbeeld fietsen. Als u in deze periode namelijk ten val komt, kan dat de wondgenezing verstoren. Hetzelfde geldt bij autorijden; in het geval dat er onverwacht een grote kracht op het borstbeen gaat werken, loopt u kans op beschadiging van uw borstbeen. Heeft u last van hartritmestoornissen, dan is het verstandig eerst met uw cardioloog te overleggen voordat u weer gaat fietsen dan wel autorijden

8022

Verkeer. Door de aandoening en de behandeling kan het zijn dat uw concentratievermogen de eerste zes weken minder is. Het is beter om in deze periode geen auto of fiets te besturen.

8935

Buiten fietsen: na 6 weken toegestaan.

Autorijden: na 6 weken toegestaan.

9042

Vliegen is vanaf 12 weken na de operatie toegestaan. Doe dit altijd in overleg met uw behandelend arts, Hij of zij weet het beste hoe de conditie van uw hart is.

9627

Fietsen. U mag fietsen op een hometrainer zonder weerstand. Buiten fietsen is de eerste 6 weken niet toegestaan in verband met het risico op vallen met een nog niet volledig genezen borstbeen.

Autorijden. Autorijden is de eerste 6 weken niet toegestaan.

9636

In de eerste vier weken: U mag niet fietsen, autorijden of andere voertuigen besturen; U mag wel op de hometrainer fietsen.

Sedentaire Leefstijl en Lichamelijke Activiteit – Vakantie

2312

Vakantie: Een week of vier na de operatie kunt u rustig op vakantie, mits u zich goed voelt. Vliegreizen of een lange inspannende reis kunt u voorlopig beter nog mee wachten. Neem voldoende medicijnen mee en vraag uw apotheek om een geneesmiddelenpaspoort waarop uw actuele medicijnen staan vermeld. Informeer van tevoren of de medische voorzieningen in het land van bestemming goed genoeg zijn. Indien u bloedverdunners via de trombosedienst gebruikt is het verstandig om met de trombosedienst te bespreken waar u terecht kunt voor controle op het gebruik van antistollingsmiddelen en vraag om een speciale "vakantiebrief" die u mee kunt nemen.

Vakantie. Na vier weken mag u in principe op vakantie gaan. Zolang het een rustige vakantie is. Naar de bergen of een andere inspannende reis kunt u het beste eerst even navragen, want dat is per persoon verschillend. Voor mogelijke calamiteiten is het handig als u uw ziektegeschiedenis, een geneesmiddelenoverzicht en een ECG bij u heeft. Dit kunt u bij uw huisarts opvragen. Indien u onder controle van de trombosedienst bent, kunt u daar bespreken waar u terecht kunt voor controle op het gebruik van antistollingsmiddelen.

5949

Vakantie. Als uw herstel naar wens verloopt, kunt u zonder bezwaar op vakantie. Bespreek dit wel eerst met uw cardioloog als u na 3 weken voor uw eerste controle komt. Bent u geopereerd? Houd dan rekening met de volgende leefregels: • u mag na 3 weken weer vliegen,

Vakantie. Als u vlot en zonder problemen van uw hartoperatie herstelt, is een vakantie heel goed mogelijk. Als u plannen heeft om op vakantie te gaan, bespreek deze dan eerst met uw cardioloog. Hij/zij bepaalt of u lichamelijk goed genoeg bent om uw reis te maken. Wacht dus eerst het advies van uw cardioloog af, voordat u een vakantie boekt!

Vakantie. Wanneer uw herstel naar wens verloopt, kunt u zonder bezwaar op vakantie. Bespreek uw plannen tijdens uw polibezoek met uw specialist. Na 3 weken mag u weer vliegen. Houd er rekening mee dat u de eerste 6 weken nog niet mag zwemmen, omdat de wond dan te week zou worden. U mag met uw operatiewond een jaar lang niet in de zon liggen: de nieuwe huid verbrandt snel. Dek daarom gedurende een jaar tijdens het zonnebaden de wond af met een pleister.

7041
Vakantie: Overleg dit met uw cardioloog.

7096
Vakantie. Als u vlot en zonder problemen herstelt, dan is ongeveer zes weken na de operatie met vakantie gaan goed mogelijk. Beperkingen zijn er in principe niet. Wel is het verstandig dit van tevoren met uw huisarts of cardioloog en, indien van toepassing, de trombosedienst te overleggen.

8022
Vakantie. Als uw herstel goed verloopt, dan kunt u gewoon op vakantie. Twijfelt u, bespreek dit dan tijdens de controleafspraak met uw specialist.

Sedentaire Leefstijl en Lichamelijke Activiteit – Werken

0535
Werkhervatting. Ook het hervatten van uw werk kunt u bespreekbaar maken bij uw cardioloog.

Beginnen met werken. Wanneer u kunt beginnen met werken is afhankelijk van het soort werk dat u doet. Als u zwaar lichamelijk werk doet, is er mogelijk meer tijd nodig voordat u kunt terugkeren. Over het algemeen is het verstandig om ongeveer 6 weken tot 3 maanden na de operatie stap voor stap uw werk te hervatten. Bespreek uw individuele omstandigheden met de maatschappelijk werker, huisarts en werkgever. Een voorspoedig herstel gewenst

Beginnen met werken Wanneer u kunt beginnen met werken is afhankelijk van het soort werk dat u doet. Als u zwaar lichamelijk werk doet, is er mogelijk meer tijd nodig voordat u kunt terugkeren. Over het algemeen is het verstandig om ongeveer 6 weken tot 3 maanden na de operatie stap voor stap uw werk te hervatten. Bespreek uw individuele omstandigheden met de maatschappelijk werker, huisarts en werkgever. Een voorspoedig herstel gewenst

2312
Werkhervatting: Meestal kunt u na drie tot zes maanden weer uw werk hervatten, afhankelijk van de aard van uw werkzaamheden. U voelt over het algemeen zelf het beste wanneer u hier weer in staat toe bent. Bespreek de werkhervatting met uw eigen cardioloog, uw huisarts en de bedrijfsarts.

Werk. Of en wanneer u weer kunt gaan werken hangt af van uw persoonlijke situatie. Wat is er precies gebeurd, wat is de behandeling geweest en wat is uw werksituatie. Over het algemeen wordt geadviseerd uw eerste polikliniekcontrole bij de cardioloog of verpleegkundige af te wachten. Voor operatiepatiënten geldt dat u na 3 tot 6 maanden uw werk weer kunt hervatten. Met uw bedrijfsarts kunt u bespreken hoe het gaat en een plan maken om weer aan het werk te gaan, of naar andere oplossingen te zoeken. Ook als en waarom werken nog niet lukt kunt u met uw bedrijfsarts bespreken. De bedrijfsarts mag medische gegevens opvragen, maar krijgt deze alleen indien u daar schriftelijk toestemming voor heeft gegeven.

4078
Werken. Het is van belang dat u zo snel mogelijk weer aan het werk gaat. Dit kan al tijdens uw hartrevalidatie. Vanuit de richtlijnen van de bedrijfsgezondheidsdienst wordt erna gestreefd dat u drie weken na uw infarct weer mag starten met uw werkzaamheden. Na een hartoperatie wordt zes weken gehanteerd. Belangrijk is dat uw eerste contact met de werkgever en bedrijfsarts ongeveer drie weken na het ontslag plaatsvindt, zodat er een plan van aanpak gemaakt kan worden voor werkhervatting. Dit in het kader van de Wet Verbetering Poortwachter.

Werk. Het is belangrijk om zo snel mogelijk weer aan het werk te gaan. Daarom raden wij u aan om tijdens het hartrevalidatietraject weer langzaam te beginnen met uw werkzaamheden. Belangrijk is dat u uw eerste contact met uw werkgever en bedrijfsarts ongeveer drie weken na het ontslag uit het ziekenhuis heeft, zodat u samen een plan kunt maken wanneer u weer aan het werk gaat. Dit in het kader van de Wet Verbetering Poortwachter.

5949
Werk. De verwachting is dat u na een halfjaar tot een jaar weer kunt werken. Dit is afhankelijk van de aard van uw werk en uw conditie. U voelt over het algemeen zelf het beste wanneer u weer aan werken toe bent. Maar bespreek ook altijd eerst met uw specialist wanneer u weer kunt werken en wat u wel en niet mag doen. Vaak is het verstandig met halve dagen te beginnen en dit langzaam op te voeren.

Werkhervatting. De meeste mensen kunnen 3 tot 6 maanden na de operatie weer gaan werken. Dit hangt onder meer af van de aard van uw werk. Wanneer u weer kunt gaan werken en wat u dan wel en niet mag doen, bespreekt u met uw eigen cardioloog en de bedrijfsarts. U voelt over het algemeen zelf het beste wanneer u weer aan werken toe bent. Vaak is het verstandig te beginnen met halve dagen en het langzaam op te bouwen.

Werken. De verwachting is dat u na een half jaar tot een jaar na de operatie weer kunt gaan werken, afhankelijk van de aard van uw werk en uw conditie. Bespreek met uw specialist wanneer u weer kunt gaan werken en wat u dan wel en niet mag doen. U voelt over het algemeen zelf het beste wanneer u weer aan werken toe bent. Vaak is het verstandig te beginnen met halve dagen werken en dit langzaam op te voeren.

 © 2024. The Author(s).

Werken: Overleg met de cardioloog, of en wanneer, u uw werkzaamheden weer kunt hervatten.

7096
Werkhervatting. Meestal kunt u uw werk hervatten tussen drie en zes maanden na de operatie, afhankelijk van de aard van de werkzaamheden. U voelt over het algemeen zelf het best wanneer u weer aan werken toe bent. Bespreek werkhervatting in een vroeg stadium met uw werkgever, bedrijfsarts, huisarts of cardioloog. Vaak is het verstandig te beginnen met halve dagen en dit langzaam uit te breiden

8022
Werken. De meeste mensen hebben een aantal maanden tot een jaar nodig voor ze weer aan het werk gaan. Dit hangt af van het werk dat ze doen en van de mate van herstel. Vaak voelt u zelf wanneer u weer aan werken toe bent. Begin liever met halve werkdagen en bouw dit langzaam op. Twijfelt u of u weer aan het werk kunt en of u alles al weer kunt doen? Bespreek dit dan met uw specialist tijdens de controleafspraak drie maanden na de opname.

8935
Werken. Werken tijdens en na de behandeling kan goed zijn voor uw welzijn en herstel. Het kan afleiding en houvast bieden. Ook kan het contact met collega's fijn zijn. Of u kunt werken en hoeveel hangt onder meer af van bijwerkingen en uw soort werk. Bespreek uw situatie met uw behandelaar.

Sedentaire Leefstijl en Lichamelijke Activiteit – Seks

0535
Seksualiteit. De belasting die seksuele activiteit van uw lichaam vraagt, vormt geen belasting. U kunt uw seksuele activiteiten weer inpassen in uw normale levenspatroon

Kan ik seks hebben na de operatie? Waarschijnlijk heeft u de eerste weken na de ingreep minder zin in seks. In verband met de bloeddruk en een nog niet helemaal genezen borstbeen is het verstandig dat u in het begin voorzichtig aan doet bij het vrijen. Langere tijd na de ingreep luistert u gewoon naar uw lichaam. Bepaalde medicijnen zoals bètablokkers (bijvoorbeeld metoprolol) kunnen impotentie als bijwerking geven. Vraag eventueel uw cardioloog naar een alternatief.

2312
Vrijen is geen activiteit die het hart extra belast. De inspanning hierbij is te vergelijken met het oplopen van een tot twee trappen.

Seks. Veel mensen hebben de eerste tijd na de ziekenhuisopname niet zoveel belangstelling voor seks. Misschien vraagt u zich af of vrijen wel verantwoord is. Vrijen is ongeveer even belastend als twee trappen lopen. Als u dat zonder echte klachten kunt doen, is er geen bezwaar tegen. Vernauwingen in de bloedvaten van de geslachtsdelen of sommige medicijnen kunnen het vrijen moeilijker of onmogelijk maken. U kunt dit met uw behandelaar of huisarts bespreken, zodat naar een oplossing gezocht kan worden.

4078
Vrijen. Wanneer u en uw partner daar aan toe zijn, kunt u weer vrijen. Dit is niet gevaarlijk voor uw hart. Wel adviseren wij u om de eerste weken voorzichtig te zijn, omdat uw borstbeen nog moet herstellen. Gevoelens rond seksualiteit - angst, schaamte, verdriet, boosheid - kunt u beter niet tegenhouden. Bespreek het in ieder geval met uw partner en eventueel met de huisarts of cardioloog of cardiovasculair verpleegkundige. Misschien zit het probleem bij de medicijnen die u slikt.

5892
Seksualiteit. Vrijen is geen activiteit die het hart extra belast, de inspanning hierbij is te vergelijken met het oplopen van twee trappen. Als u zonder problemen kunt traplopen is vrijen geen probleem.

5949
Seksualiteit. U kunt in principe zonder bezwaar vrijen en gemeenschap hebben. Dit is niet gevaarlijk voor uw aorta. Bent u geopereerd? Wees dan de eerste weken wel voorzichtig met de kwetsbare wond. Met vragen over seksualiteit na uw operatie kunt u altijd terecht bij uw specialist.

Vrijen. Wanneer u en uw partner daar aan toe zijn, kunt u weer vrijen. Dit is niet gevaarlijk voor uw hart. Wel adviseren wij u om de eerste weken voorzichtig te zijn, omdat uw wond (borstbeen) nog moet herstellen. Met vragen over seksualiteit na uw operatie kunt u altijd bij uw behandelend arts, de verpleegkundig specialist of bij de verpleging terecht.

Seksualiteit. Als u en uw partner daar aan toe zijn, kunt u zonder bezwaar vrijen en gemeenschap hebben. Dit is niet gevaarlijk voor uw aorta. Wel adviseren wij u om de eerste weken voorzichtig te zijn, omdat de wond nog kwetsbaar is. Met vragen over seksualiteit na uw operatie kunt u altijd terecht bij uw specialist.

7037
Praten over seks is lastig, zowel voor de patiënt als voor de arts en de verpleegkundige. Het is moeilijk om algemene richtlijnen te geven hoe hier mee om te gaan na (of met) een hartaandoening. Daarbij is het beleven van de seksualiteit en intimiteit zeer persoonlijk en heeft een ieder hier zijn eigen patroon in ontwikkeld. Toch willen wij een paar opmerkingen maken.

Het is heel normaal als u in de herstelfase geen zin in seks heeft. Het verwerkings- proces van het hebben van een hartaandoening en opbouwen van conditie heeft tijd nodig. Voor de eerste keer weer vrijen na de ziekenhuisopname is spannend en soms valt het tegen. Wees niet te snel teleurgesteld als het niet verloopt zoals u wenst. Zowel u als uw partner zullen weer vertrouwen moeten krijgen, de angst

 © 2024. The Author(s).

overwinnen en opnieuw vertrouwd moeten raken met elkaar. Dit kost tijd. Laat u niet weerhouden om gewoon het op een ander moment opnieuw te proberen.

7041
Seksualiteit: In principe is dit geen bezwaar. Dit is niet schadelijk voor uw aorta. Wees wel voorzichtig met de wond, deze is nog kwetsbaar.

7096
Seksualiteit. U hoeft niet bang te zijn om te vrijen. Vrijen betekent geen extra risico voor uw hart. De inspanning hierbij is te vergelijken met het oplopen van twee trappen. Ook de operatiewond in de borstkas is na ontslag voldoende genezen. Het is na een grote operatie echter niet ongewoon dat het vrijen niet meteen is als voorheen. Soms kan uw partner zich schamen, omdat hij of zij zin heeft in seks en bang is u daarmee lichamelijk of emotioneel teveel te belasten. Het is belangrijk om ook hierover met elkaar proberen te praten. Het gebruik van bepaalde medicijnen (de zogenoemde bètablokkers) kan erectieproblemen geven bij mannen en bij vrouwen ervoor zorgen dat de vagina minder vochtig wordt.

8022
Vrijen. Er is geen bezwaar tegen vrijen, als u en uw partner daaraan toe zijn. Als u het rustig aan doet, is er geen risico voor uw aorta.

Mentaal Welzijn

0535
Het is bekend dat mensen na zo'n grote operatie angst en onzekerheid voelen bij het hervatten van activiteiten. U bent het vertrouwen in uw lichaam kwijt en u weet niet zeker of u deze activiteiten wel weer zonder risico's kunt uitvoeren. Als u geleidelijk aan meer gaat doen, zal de onzekerheid afnemen en het zelfvertrouwen toenemen.

Door vermoeidheid kan uw concentratievermogen sneller verminderen. Dit merkt u bijvoorbeeld bij het lezen of tijdens een gesprek.

Het is mogelijk dat u in de periode na de operatie naar droomt. Dit kan worden veroorzaakt door de ervaringen tijdens de operatie of het verblijf op de intensive care. In het algemeen nemen deze dromen twee tot drie weken na de operatie geleidelijk af . Als dit niet zo is, neem dan contact op met uw huisarts of bespreek het met de revalidatiearts.

2312
Angst is een natuurlijke, gezonde reactie van ons lichaam. Deze reactie zorgt ervoor dat we alert kunnen reageren op gevaarlijke situaties. Ook een hartaandoening brengt vaak angst met zich mee. Hoe groot deze angst is, en hoelang deze duurt, hangt onder andere af van uw persoonlijkheid. Als u bijvoorbeeld snel nerveus bent, onzeker bij onverwachte zaken, neigt naar doemdenken, of eindeloos kunt piekeren, dan kan angst uw dagelijks leven sterk beïnvloeden. Ook ingrijpende gebeurtenissen die u eerder in uw leven al meemaakte kunnen invloed hebben op de angst die u nu ervaart.

Angst heeft invloed op zowel ons lichaam als ons gedrag, onze emoties en op ons denken. Het is van belang om bij uzelf en/of uw partner te herkennen of u angstig bent en hóé angstig u bent. Mogelijk past de mate van angst wel bij wat u meemaakte en/of hebt u zelf manieren gevonden om met uw angst om te gaan.

Als uw angsten niet meer "normaal" te noemen zijn, dan kan er sprake zijn van een angststoornis of een piekerstoornis. Bij een angst- of piekerstoornis lukt het u niet meer om te stoppen met piekeren over uw hartaandoening en de gevolgen ervan. U kunt daardoor niet meer ontspannen en dat kan leiden tot slaapproblemen. Ook gaat u misschien bepaalde plekken of activiteiten vermijden, waardoor problemen op sociaal gebied of op het werk kunnen ontstaan.

Deze vorm van vermijding en té alert zijn op lichamelijke reacties, zoals pijn in de borststreek, kan een negatief effect hebben op uw gezondheid. Het is daarom van belang om aandacht te besteden aan het leren omgaan met uw angst.

Het kost tijd om van een hartaandoening te herstellen. Zowel lichamelijk als geestelijk. Het herstel verloopt bij iedereen anders. Lichamelijke ziekten zoals een hartaandoening, kunnen bij sommige mensen zelfs een angststoornis uitlokken. De ernst en de duur van zo'n angststoornis verschillen per persoon en laten zich niet voorspellen.

Als u en/of uw partner last hebben van angstklachten, dan is het verstandig om daarvoor hulp te zoeken. Veel mensen volgen hiervoor een hartrevalidatieprogramma. Zo'n programma bestaat uit verschillende modules.

Het volgen van meerdere modules zorgt dat u zich bewuster wordt van de gewenste en noodzakelijke aanpassingen in uw gedrag en leefstijl. Hierdoor vermindert u de kans op nieuwe hartproblemen en leert u bovendien beter met uw aandoening omgaan.

Intense angst heeft invloed op zowel ons lichaam als ons gedrag, onze emoties en ons denken. Het is van belang om bij uzelf en/of uw partner te herkennen of er sprake is van een paniekaanval. Mogelijk past de mate van paniek wel bij wat u meemaakte en/of hebt u zelf manieren gevonden om met de intense angst om te gaan.

Als de paniekaanvallen herhaaldelijk terugkeren en u voortdurend bang bent voor nieuwe paniekaanvallen, kan er sprake zijn van een paniekstoornis. Bij een paniekstoornis lukt het u niet meer om te vertrouwen op uw lichaam en/of te ontspannen. U zoekt bijvoorbeeld steeds geruststelling bij een arts, u controleert uw bloeddruk vaker of u beperkt uw lichamelijke inspanning fors. Soms kunt tegen de nacht opzien en dat kan leiden tot slaapproblemen. Ook gaat u misschien bepaalde plekken of activiteiten vermijden, waardoor problemen op sociaal gebied of op het werk kunnen ontstaan. Deze vorm van vermijding en het té alert zijn op lichamelijke verschijnselen, kan een negatief effect hebben op uw gezondheid. Het is daarom van belang om aandacht te besteden aan het omgaan met een paniekaanval

 © 2024. The Author(s).

Als u en/of uw partner last hebben van paniekklachten, dan is het verstandig om daarvoor hulp te zoeken. Veel mensen volgen hiervoor een hartrevalidatieprogramma. Zo'n programma bestaat uit verschillende modules. Het volgen van meerdere modules zorgt dat u zich bewuster wordt van de gewenste en noodzakelijke aanpassingen in uw gedrag en leefstijl. Hierdoor vermindert u de kans op nieuwe hartproblemen en leert u bovendien beter met uw aandoening omgaan. Elk hartrevalidatieprogramma start met een uitgebreid intakegesprek met de hartverpleegkundige. Afhankelijk van uw wensen kunt u vervolgens nog uitgenodigd worden voor een gesprek bij bijvoorbeeld medische psychologie, fysiotherapie, medisch maatschappelijk werk of psychiatrie.

Somberheid en verdriet horen bij het leven. Het zijn normale reacties op nare gebeurtenissen. Ook een hartaandoening brengt soms somberheid met zich mee. Deze somberheid is niet bij iedereen in dezelfde mate en duur aanwezig. Dit hangt onder andere af van uw persoonlijkheid. Als u bijvoorbeeld snel bezorgd en/of prikkelbaar bent, snel moe, neigt naar doemdenken, of eindeloos kunt piekeren, dan kan een hartaandoening bij u eerder leiden tot somberheid.

Ook lichamelijke klachten en ingrijpende gebeurtenissen die u eerder in uw leven meemaakte, kunnen nu nog tot somberheid leiden. Soms speelt een erfelijke aanleg een rol. Somberheid heeft invloed op zowel ons lichaam als ons gedrag, onze emoties en op ons denken. Het is van belang om bij uzelf en/of uw partner te herkennen of u somber bent en hóé somber u bent. Mogelijk past de mate van somberheid wel bij wat u meemaakte en/of hebt u zelf manieren gevonden om met uw somberheid om te gaan.

Als uw sombere gevoelens niet meer "normaal" te noemen zijn omdat u eronder lijdt, dan kan er sprake zijn van een depressie. Bij een depressie lukt het u niet meer om te stoppen met piekeren en komt in een neerwaartse spiraal terecht. U kunt daardoor niet meer ontspannen en u verliest uw interesse en energie om dingen te ondernemen. Ook kunt u sneller uit uw slof schieten dan voorheen. Of u gaat zich juist terugtrekken uit het contact met andere mensen, waardoor problemen op sociaal gebied of op het werk kunnen ontstaan.

Somberheidsklachten hebben veel impact op u en uw naasten. Bovendien zijn ze van invloed op het functioneren van uw hart en hebben dus een negatief effect op uw gezondheid. Het is daarom van belang om aandacht te besteden aan het leren omgaan met somberheid.

Het kost tijd om van een hartaandoening te herstellen. Zowel lichamelijk als geestelijk. Het herstel verloopt bij iedereen anders. Lichamelijke ziekten zoals een hartaandoening, kunnen somberheid en soms een depressie uitlokken. De ernst en de duur daarvan verschillen per persoon en laten zich niet voorspellen.

Als u en/of uw partner last hebben van sombere gevoelens, dan is het verstandig om daarvoor hulp te zoeken. Veel mensen volgen hiervoor een hartrevalidatieprogramma. Zo'n programma bestaat uit verschillende modules. Het volgen van meerdere modules zorgt dat u zich bewuster wordt van de gewenste en noodzakelijke aanpassingen in uw gedrag en leefstijl. Hierdoor vermindert u de kans op nieuwe hartproblemen en leert u bovendien beter met uw aandoening omgaan.

Stress of spanning is een natuurlijke, gezonde reactie van ons lichaam. Deze reactie zorgt ervoor dat we goed kunnen presteren en alert kunnen reageren op gevaarlijke situaties. Ook een hartaandoening brengt vaak stress met zich mee. Hoe groot deze spanningen zijn en hoelang ze duren, hangt onder ander af van uw persoonlijkheid. Als u bijvoorbeeld snel nerveus bent, te veel wilt, moeilijk uw grenzen kunt aangeven, zaken opkropt of eindeloos kunt piekeren, dan kunnen de spanningen uw dagelijks leven sterk beïnvloeden.

Ook ingrijpende gebeurtenissen die u eerder in uw leven al meemaakte kunnen invloed hebben op de stress die u nu ervaart. Soms speelt erfelijke aanleg een rol, mogelijk in combinatie met angst en/of een depressie. Stress heeft invloed op zowel ons lichaam als ons gedrag, onze emoties en ons denken. Het is van belang om bij uzelf en/of uw partner te herkennen of u gestrest en hóé gestrest u bent. Mogelijk past de mate van stress wel bij wat u meemaakte en/of hebt u zelf manieren gevonden om met uw stress om te gaan.

Als uw stress niet meer "normaal" te noemen is, omdat u eronder lijdt, dan kan er sprake zijn van ongezonde stress. Bij ongezonde stress zijn de eisen die aan u worden gesteld en/of die u aan uzelf stelt, groter dan u aankunt. U kunt daardoor bijvoorbeeld niet meer ontspannen en dat kan weer leiden tot slaapproblemen. Ook gaat u misschien minder gezond leven, rookt, drinkt meer alcohol, eet minder gezond of beweegt nauwelijks. Daardoor vergroot u de kans op hogere bloeddruk of cholesterolwaarden, ontstekingsreacties en/of de vorming van bloedstolsels. Bovendien kan stress u een opgejaagd gevoel geven en snel irritaties teweegbrengen, waardoor problemen op sociaal gebied of op het werk kunnen ontstaan.

Deze vorm van leefstijl en gespannenheid kunnen een negatief effect hebben op uw gezondheid. Het is daarom van belang om aandacht te besteden aan het leren omgaan met uw stress.

Het kost tijd om van een hartaandoening te herstellen. Zowel lichamelijk als geestelijk. Het herstel verloopt bij iedereen anders. Lichamelijke ziekten zoals een hartaandoening, kunnen soms ongezonde stress uitlokken. De ernst en de duur daarvan verschillen per persoon en laten zich niet voorspellen. Als u en/of uw partner last hebben van stressklachten, dan is het verstandig om daarvoor hulp te zoeken. Veel mensen volgen hiervoor een hartrevalidatieprogramma. Zo'n programma bestaat uit verschillende modules. Het volgen van meerdere modules zorgt dat u zich bewuster wordt van de gewenste en noodzakelijke aanpassingen in uw gedrag en leefstijl. Hierdoor vermindert u de kans op nieuwe hartproblemen en leert u bovendien beter met uw aandoening omgaan. Elk hartrevalidatieprogramma start met een uitgebreid intakegesprek met de hartverpleegkundige. Afhankelijk van uw wensen kunt u vervolgens nog

 © 2024. The Author(s).

uitgenodigd worden voor een gesprek bij medische psychologie, fysiotherapie, medisch maatschappelijk werk of psychiatrie

Sommige mensen voelen zich onzeker en/of angstig, nadat ze last van hun hart hebben gekregen. Van de “veilige” omgeving op de verpleegafdeling, moet u het thuis zien te stellen zonder dokter of verpleegkundige in de buurt. Uw arts is van mening dat u zo ver hersteld bent dat u deze “veilige” omgeving niet meer nodig heeft. Probeer rustig aan thuis weer de draad op te pakken. Indien u angstig blijft, zodat het bijvoorbeeld niet lukt om te stoppen met piekeren, of u plekken of activiteiten blijft vermijden, neem dan contact op met uw huisarts of hartrevalidatieverpleegkundige.

Depressie bij hartklachten komt vaker voor dan u denkt. Het is niet altijd makkelijk om verder te leven met het idee dat “je iets aan je hart hebt gehad”, de motor van het lichaam. Het kan tijd kosten het vertrouwen in uw lichaam weer terug te krijgen. U kunt zich de eerste tijd somber of bang voelen en misschien moeilijk slapen. Praten over uw gevoelens is dan belangrijk. Het hoort bij de verwerking. Praat met uw huisarts, cardioloog of hartrevalidatieverpleegkundige over wat u voelt. Mochten de klachten niet verbeteren, dan kunt u eventueel ook doorverwezen worden voor begeleiding en hulp.

Stress op zichzelf is niet schadelijk, het zorgt er juist voor dat mensen goede prestaties kunnen leveren. Het wordt een probleem als de stress te lang aanhoudt en er geen mogelijkheden voor het lichaam zijn om te ontspannen. Stress verhoogt dan de kans op een hartziekte. Vaak is de oorzaak van de stress moeilijk aan te pakken. Wel kunt u proberen er zo goed mogelijk mee om te gaan. Regelmatige en intensieve beweging vermindert de gevoeligheid voor stress. Voor meer informatie kunt u contact opnemen met de hartrevalidatieverpleegkundige of uw huisarts.

Ieder van ons kent wel enige vorm van stress. Dat is ook heel normaal. Teveel stress is echter niet goed. Het is belangrijk om de juiste balans tussen stress en ontspanning te vinden. Teveel stress kan verschillende klachten veroorzaken, waaronder duizeligheid, hoofdpijn, hartkloppingen, pijn in nek of rug, hoge bloeddruk en maag- of darmklachten. Bij beginnende stress is het belangrijk ervoor te zorgen dat de stress niet tot ernstige klachten leidt. Een aantal nuttige tips kunnen u hierbij helpen: Pieker niet over het verleden of de toekomst, u leeft nu!

Praat over uw problemen. Samen ziet u meer oplossingen dan alleen. Lijken uw problemen het gevolg te zijn van uw werk, praat er dan over met uw leidinggevende. Probeer samen de oorzaak te vinden en kijk naar mogelijke oplossingen. Daarnaast is het belangrijk om voldoende te slapen.

3748

Na een hartprobleem of om andere redenen kunt u last hebben van spanning (stress). Voorbeelden hiervan zijn: slecht slapen, concentratieverlies, prikkelbaarheid, pijn in de spieren, gespannen gevoel, vermoeidheid, onvoldoende resultaat van de training, hartkloppingen of hoge bloeddruk. In deze cursus leert u hoe hier mee om te gaan.

4078

Ontspanning is goed voor het hart. Ontspanning heeft een positieve invloed op de hartslag en de bloeddruk. Doel van deze cursus is het bewust worden en loslaten van spanning door middel van ademhalingsoefeningen en lichaamsbewustzijn. U neemt eerst deel aan een introductie les onder begeleiding van de fysiotherapeut. Daarna kunt u gedurende vijf weken een keer per week deelnemen aan deze module.

Een hartoperatie is een ingrijpende gebeurtenis die naast lichamelijke vaak ook emotionele gevolgen heeft. U kunt bijvoorbeeld sneller geëmotioneerd raken of sneller gaan huilen. Ook boosheid, driftbuien en snel geïrriteerd raken, zijn verschijnselen die bij de verwerking van een hartoperatie kunnen optreden. Dit kan voor spanning in de relatie met uw partner zorgen.

Daarnaast kunt u zich onzeker voelen als u weer begint met activiteiten. Dit komt doordat u niet zeker weet of u deze activiteiten wel weer zonder risico’s kunt doen. Als u langzaam meer gaat doen, neemt de onzekerheid af en het zelfvertrouwen toe.

Door de narcose en/of vermoeidheid kan uw concentratievermogen minder worden. Dit merkt u bijvoorbeeld bij lezen of tijdens een gesprek.

5949

Bij thuiskomst zult u zich nog niet helemaal optimaal voelen. Waarschijnlijk is uw concentratievermogen minder dan normaal en bent u emotioneler dan anders. U kunt bijvoorbeeld zomaar huilen of geïrriteerd raken, terwijl u de volgende dag nergens last van heeft. Dit is normaal na een hartoperatie. Het komt door het gebruik van de hart-longmachine, de narcose en alles wat u in het ziekenhuis heeft meegemaakt. Door het oppakken van uw normale leven merkt u dat het vanzelf beter gaat. Gun uzelf de tijd om weer op krachten te komen.

Na een TAAA-operatie kunt u zich emotioneel onzeker voelen. U kunt bijvoorbeeld zomaar huilen of geïrriteerd raken, terwijl u de volgende dag nergens meer last van hebt. Deze stemmingswisselingen worden veroorzaakt door het gebruik van de hartlongmachine tijdens de operatie. De klachten verdwijnen na verloop van tijd, wanneer u verder op krachten komt

PROTOCOL Angst en onzekerheid kunnen het leven van de patiënt en van de familie nog beheersen. Het is een spannende ervaring te merken of de operatie wel of (nog) geen resultaat heeft gehad. De pijn van de wond is soms moeilijk te onderscheiden met de pijn op de borst van voor de operatie. Het vertrouwen in het eigen lichaam moet opnieuw opgebouwd worden.

PROTOCOL Angst en onzekerheid kunnen het leven van de patiënt en van de familie nog beheersen. Een TAA operatie is een ingrijpende, zware en risicovolle operatie. De patiënt heeft dan ook vaak enkele maanden tot een jaar nodig om aan te sterken.

7037

Vrijen is op zich een ontspannende activiteit, maar door een aandoening aan het hart kunt u het gevoel krijgen dat het een

 © 2024. The Author(s).

te inspannende bezigheid is. Deze inspanning kan zowel bij u als bij de partner angst en onzekerheid geven. Als de partner bang is voor het opnieuw ontstaan van hartklachten bij u, zal hij/zij misschien afzien van seksualiteit. Een open gesprek over angst, bezorgdheid en de onzekerheid die beiden voelen, kan die spanning voorkomen. Het heeft geen zin om elkaar te ontzien of te forceren. Belangrijk is om met elkaar te vrijen wanneer beiden daar aan toe zijn.

Het is heel normaal dat u zich moe of labiel voelt na de operatie. Veel mensen krijgen huilbuien. Ook andere emotionele uitbarstingen komen voor. Het zijn ontladingen van alle spanningen voor de operatie. Misschien voelt u zich er ongemakkelijk bij, maar geef er vooral aan toe. Want het is goed voor uw herstel. Sommige mensen raken na de operatie gedesoriënteerd. Ze zijn in de war, zien dingen die er niet zijn en zijn hun dag- en nachtritme kwijt. Dit gaat meestal na een paar dagen over. Duurt het langer en heeft u misschien het gevoel dat u "een stukje uit uw leven mist"? Maakt u zich niet teveel zorgen. Het hoort erbij en het gaat over.

Als alles voorbij is, voelt u naast opluchting misschien ook ontlading, omdat u een spannende tijd meemaakte. En u moet misschien weer vertrouwen in het leven en uw lichaam krijgen. Het is verstandig om over uw gevoelens, angsten en gedachten met anderen te praten, bijvoorbeeld met uw partner, familieleden, vrienden en collega's. Via de patiëntenvereniging De Hart&Vaatgroep kunt u contact leggen met een lotgenoot bij u in de regio. Kijk hiervoor op www.hartenvaatgroep.nl. Ook het hartrevalidatieprogramma kan u veel steun bieden. Bent u lusteloos, heeft u weinig eetlust en lukt het u maar niet om goed te slapen? Dat zijn signalen om goed in de gaten te houden. Ze kunnen namelijk wijzen op neerslachtigheid. Neem in dat geval contact op met uw huisarts.

Stress kan het risico op hart- en vaatziekten vergroten. Er is een verschil tussen acute stress en chronische stress. In situaties van acute stress, zoals bijvoorbeeld een ongeluk, oorlogssituaties of andere sterke emoties neemt het risico op hart- en vaatziekten toe. Ook chronische stress, zoals financiële of relationele problemen, werkstress, ziekte of sterfte in de familie, angst of depressie, kan zorgen voor een verhoogd risico op hart- en vaatziekten. Psychologische reacties op het hart moeten niet onderschat worden, het is daarom van belang om stress zo veel mogelijk te vermijden

Het kan zijn dat u na de operatie sneller geëmotioneerd bent. Hierdoor huilt u makkelijker, ook wanneer hiervoor geen aanleiding lijkt te bestaan. Daarnaast kunt u concentratieproblemen hebben of vergeetachtig zijn. Deze verschijnselen zijn normaal bij het verwerken van een ingrijpende gebeurtenis zoals een hartoperatie. Zij verdwijnen allemaal in de loop van een paar weken tot maanden. Uw gezinsleden hebben ook een spannende tijd achter de rug. Van hen wordt vaak verwacht dat ze sterk zijn en dat zij u opvangen. Of dat ze niet laten zien dat zij het ook moeilijk hebben. Het heeft geen zin om emoties te onderdrukken. Het advies is om samen de operatie te verwerken en de gebeurtenis langzaam een plaats te geven in het leven. Vertrouw erop dat de reacties normaal en in de meeste gevallen tijdelijk van aard zijn. Ook al zijn ze pijnlijk of lastig.

Met anderen praten over de operatie kan prettig zijn. Bedenk daarbij wel dat het herstel bij iedereen anders verloopt. Hoe snel u herstelt, hangt onder meer af van het soort hartoperatie dat u hebt ondergaan. Ook uw leeftijd en uw conditie voor de operatie spelen een belangrijke rol.

7041
Als u weer thuis bent zult u zich mogelijk onzeker en emotioneel voelen. U kunt last hebben van stemmingswisselingen. Dit wordt veroorzaakt door alles wat u hebt meegemaakt. De klachten verdwijnen na verloop van tijd wanneer u weer op krachten komt.

8022
Sommige mensen voelen zich wat emotioneel en onzeker na de aortadissectie. Ze moeten onverwachts huilen, of ze zijn opeens snel geïrriteerd en het volgende moment weer rustig. Deze stemmingswisselingen komen door de behandeling en de ziekenhuisopname en zijn niet ongewoon. Bij een verder herstel verdwijnen ze.

Familie en Naasten

0535
De eerste week moet er 's nachts en een groot deel van de dag iemand bij u thuis zijn. Het ontvangen van bezoek is leuk, maar vermoeiend. Probeer het bezoek daarom goed verspreid te plannen en geef duidelijk aan wanneer u moe bent.

2312
Zorg dat u in de eerste weken thuis kunt terugvallen op iemand in gevallen van nood, zoals naaste familie, vrienden of thuiszorg.

Als u na thuiskomst niet terug kunt vallen op uw partner of gezin, is het wenselijk dat u iets regelt met iemand waar u in geval van nood een beroep op kunt doen. Vooral de eerste weken thuis is het prettig als iemand regelmatig contact met u houdt.

5949
Zorg dat u de eerste 10 dagen overdag (grootste deel) en 's nachts niet alleen thuis bent. Als u zich dan niet lekker voelt, kan degene die bij u is voor u zorgen en/of zo nodig contact opnemen met uw huisarts of specialist. Na 10 dagen is dit niet meer nodig.

We adviseren u om na een hartoperatie de eerste 7 dagen na de operatie 's nachts en grote delen van de dag iemand bij u te hebben. De dagen na uw operatie in het ziekenhuis mag u hierin meerekenen. Uw operatiedag geldt daarbij als dag 0.

We adviseren u om na een TAAA operatie de eerste 14 dagen na ontslag 's nachts en grote delen van de dag iemand bij u te hebben. Dit kunnen uw partner, vrienden en/of kennissen zijn. Zij kunnen u bij u thuis opvangen, maar dit kan ook bij iemand anders in huis zijn. De persoon die u opvangt en ondersteunt hoeft niet steeds dezelfde persoon te zijn.

We raden u aan om vóór uw opname in het ziekenhuis te bedenken of u na de operatie thuis voldoende opvang hebt

 © 2024. The Author(s).

7037
Neem uw partner of andere naaste mee naar de afspraken met het ziekenhuis.

Het kost tijd om van een hartklepoperatie te herstellen, zowel lichamelijk als geestelijk. U heeft de eerste periode nog een aantal klachten. En misschien bent u niet in staat om goed voor uzelf te zorgen en uw huishouden te doen. Daarom is het goed als er in ieder geval de eerste week iemand bij u is. Misschien willen uw partner, familieleden of vrienden u helpen. En misschien had u bij ontslag uit het ziekenhuis (aan de transferverpleegkundige) al aangegeven dat u thuiszorg nodig heeft. Dan zal er snel iemand van de thuiszorgorganisatie bij u langs komen om uw situatie te bespreken.

Het is aan te raden om vóór de opname na te denken of u na uw operatie voldoende opvang heeft. Hoewel u na de operatie alweer vrij snel een aantal dingen zelf kunt doen, zijn er ook dagelijkse handelingen die u in het begin nog niet zelf kunt uitvoeren. Denk aan huishoudelijk werk en het tillen van zware boodschappen. Het geeft u meer rust tijdens de opname als u weet dat dit soort zaken geregeld zijn.

8022
De eerste zeven dagen is het beter om 's nachts en het grootste deel van de dag niet alleen thuis te zijn. Als u zich niet lekker voelt, kan iemand voor u zorgen en zo nodig de huisarts bellen. Na zeven dagen is constante begeleiding minder noodzakelijk.

9636
Vraag iemand anders om met u mee te gaan, bijvoorbeeld een familielid, een vriend of collega. Of kijk op www.beweegmaatje.nl

 © 2024. The Author(s).
